# Comparing organic and metallo-organic hydrazone molecular cages as potential carriers for doxorubicin delivery†

**DOI:** 10.1039/d4sc02294g

**Published:** 2024-06-07

**Authors:** Giovanni Montà-González, David Bastante-Rodríguez, Alba García-Fernández, Paul J. Lusby, Ramón Martínez-Máñez, Vicente Martí-Centelles

**Affiliations:** a Instituto Interuniversitario de Investigación de Reconocimiento Molecular y Desarrollo Tecnológico (IDM), Universitat Politècnica de València, Universitat de València Camino de Vera, s/n 46022 Valencia Spain vimarce1@upv.es; b CIBER de Bioingeniería Biomateriales y Nanomedicina (CIBER-BBN), Instituto de Salud Carlos III 28029 Madrid Spain; c Unidad Mixta UPV-CIPF de Investigación en Mecanismos de Enfermedades y Nanomedicina, Valencia, Universitat Politècnica de València, Centro de Investigación Príncipe Felipe Avenida Eduardo Primo Yúfera, 3 46012 Valencia Spain; d Unidad Mixta de Investigación en Nanomedicina y Sensores, Universitat Politècnica de València, Instituto de Investigación Sanitaria La Fe (IISLAFE) Avenida Fernando Abril Martorell, 106 46026 Valencia Spain; e Departamento de Química, Universitat Politècnica de València Camí de Vera s/n 46022 Valencia Spain rmaez@qim.upv.es; f EaStCHEM, School of Chemistry Joseph Black Building, David Brewster Road EH93FJ Edinburgh UK

## Abstract

Molecular cages are three-dimensional supramolecular structures that completely wrap guest molecules by encapsulation. We describe a rare comparative study between a metallo-organic cage and a fully organic analogous system, obtained by hydrazone bond formation self-assembly. Both cages are able to encapsulate the anticancer drug doxorubicin, with the organic cage forming a 1 : 1 inclusion complex with μM affinity, whereas the metallo-organic host experiences disassembly by interaction with the drug. Stability experiments reveal that the ligands of the metallo-organic cage are displaced in buffer at neutral, acidic, and basic pH, while the organic cage only disassembles under acidic conditions. Notably, the organic cage also shows minimal cell toxicity, even at high doses, whilst the doxorubicin-cage complex shows *in vitro* anti-cancer activity. Collectively, these results show that the attributes of the pure organic molecular cage are suitable for the future challenges of *in vivo* drug delivery using molecular cages.

## Abstract 2

A comparative study between a metallo-organic cage and a fully organic analogous system shows that the fully organic molecular cage has a suitable stability and doxorubicin affinity for *in vitro* anticancer drug delivery.

## Graphical abstract

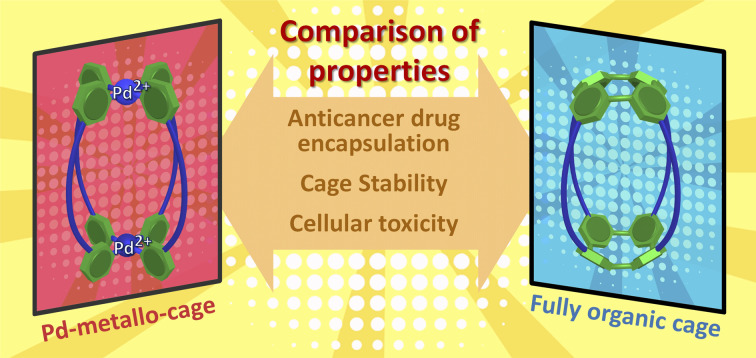

## Introduction

Supramolecular chemistry has been largely driven by the design and study of host molecules that use non-covalent interactions for efficient guest binding.^1,2^ For over 60 years, the structures of synthetic hosts have changed enormously, evolving from acyclic, macrocyclic, to fully three-dimensional cage structures that completely wrap guest molecules.^3,4^ Despite the challenges of synthesis, the development of efficient self-assembly methods has enabled the synthesis of cages with almost any possible geometry.^5–7^ Self-assembly allowed preparing molecular cages by the design of ligands with a specific shape to self-assemble into metallo-cages and purely organic cages.^5,6^ For this, it is possible to use either reversible metal–ligand interactions, to give coordination cages, or dynamic covalent chemistry to yield purely organic cages.^8,9^

Cage synthesis is mainly based on the self-assembly of building blocks with complementary connectivity and geometry under thermodynamic control.^10^ Many studies on molecular cages have focused on establishing efficient synthetic protocols, allowing multigram-scale production of cages in some cases, facilitating large-scale synthesis for different applications.^11,12^ Moreover, the careful design of the building blocks allows a precise control of the cavity size, shape, and inward-facing functionalisation for selective and efficient encapsulation.^13–15^ Main uses of molecular cages include catalysis,^16–19^ stabilisation of species,^20,21^ molecular recognition,^5,6^ release of encapsulated guests,^5,6,22^ sensing,^23^ separation processes,^13,24,25^ among many others.^26,27^ However, the exploration of molecular cages in biological and biomedical contexts remains nascent.^5,6,28–32^

Preparing cages for bio-medical applications presents a unique set of challenges, most obviously developing systems that are compatible with biological conditions, such as the pre-requisite for water solubility.^5,6,33^ In general, fully organic cages have low solubility in water due to their lack of charge and hydrophobic nature, requiring water solubilizing groups in some instances or the use of organic cosolvents.^5^ In contrast, metallo-cages are typically charged species that can result in water solubility without water solubilizing groups or cosolvents.^6^ Another challenge is the dilution effects and the presence of biological analytes that can also produce significant issues to the integrity of systems that are often inherently dynamic (despite cooperative chelate effects).^5,6,56^

Despite the challenges, the research developed in recent years shows the feasibility of using molecular cages for *in vitro* applications, with some examples of *in vivo* treatments.^28,29,34,35^ The versatility of cage assemblies holds the promise to overcome common drawbacks of conventional anticancer drugs, such as poor solubility and stability in physiological environments,^36,37^ overcome drug resistance,^38^ and reduce side effects on healthy tissues.^39^ Some relevant examples include metallo-organic cages for anticancer drug delivery (*e.g.*, cisplatin, oxaliplatin, 5-fluorouracil, (+)-camptothecin, (acac)_2_Pd, (acac)_2_Pt, caffeine, *etc.*),^40–48^ metallo-organic cages for intracellular release of photosensitizers,^49,50^ crystalline nanoparticles formed by organic cages for removing doxorubicin and irinotecan by complexation,^51^ and Pd_2_L_4_ and Pt_2_L_4_ cages with native toxicity against cells.^52,53^ Despite the work carried out in the field, research focuses on only one type of cage, particularly on metallo-organic cages, and the comparison between fully organic cages and metallo-organic cages remains unexplored.

**Fig. 1:**
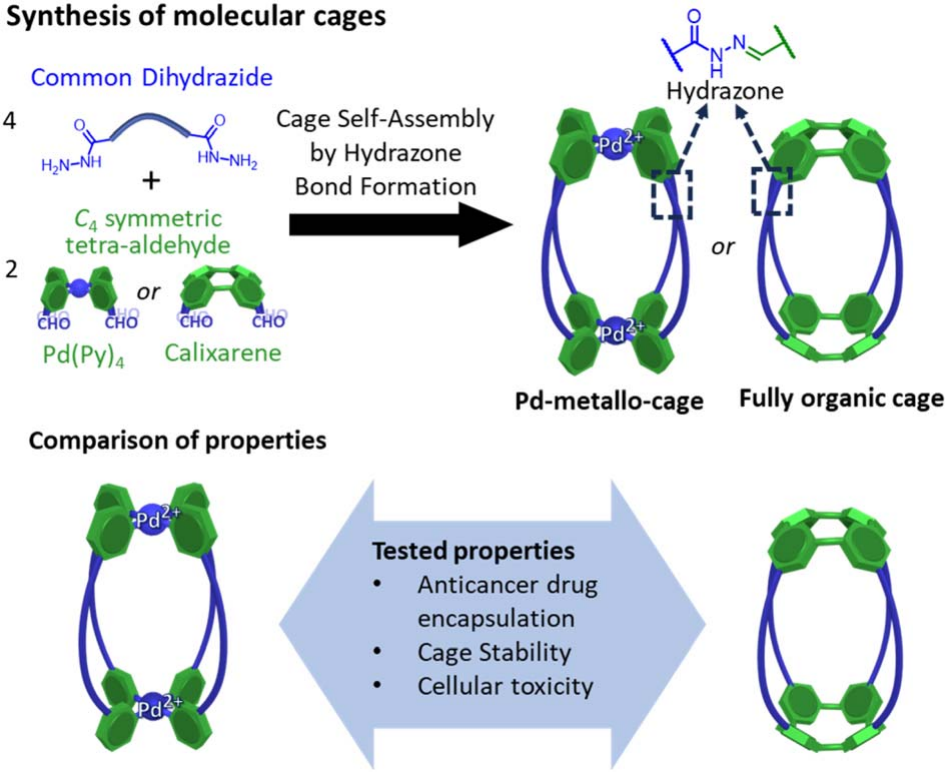
Synthesis of molecular cages containing hydrazone bonds showing the main objective of the work focused on the comparison of properties of a metallo-cage with a fully organic cage.

Here we report a comparative study of a fully organic cage and a metallo-organic cage, focusing on their host–guest properties, cage stability, and cellular toxicity (Fig. 1). Both systems are based on four dihydrazine “struts”, yet they differ in the *C*_4_ symmetric building block that serve to cap these units; the metal–organic cage exploits a square planar tetrapyridyl Pd(ii) motif whereas the organic cage uses a calixarene. In this work we have tested the water-solubility of the cages, the affinity to encapsulate the anticancer drug doxorubicin, the cellular toxicity of the unloaded cages and the therapeutic activity of the fully organic cage loaded with doxorubicin. To the best of our knowledge this is the first comparative report of similarly shaped organic and metallo-organic cages, focusing on doxorubicin encapsulation and cell toxicity.

## Results and discussion

### Synthesis of cages

In the design of the cages, we were interested in selecting a water-stable chemical group obtainable through a reversible reaction, to self-assemble the target structure from the corresponding building blocks. A structure containing hydrazone groups appeared an ideal choice, as these bonds are several order of magnitude more hydrolytically stable compared to groups such as imines at neutral pH,^54,55^ yet are still dynamic under acidic conditions (pH 5 or below).^8,54,56–59^ Cages containing hydrazone groups will remain stable in healthy tissues where the pH is in the range of 7.3–7.4, whereas cage breakdown will take place inside cells in the lysosomes (pH of *ca.* 4.5). The design might also be useful in targeting some human tumors with pH values as low as 5.6, although most frequent values are in the range of 6.4–7.^60^

**Fig. 2:**
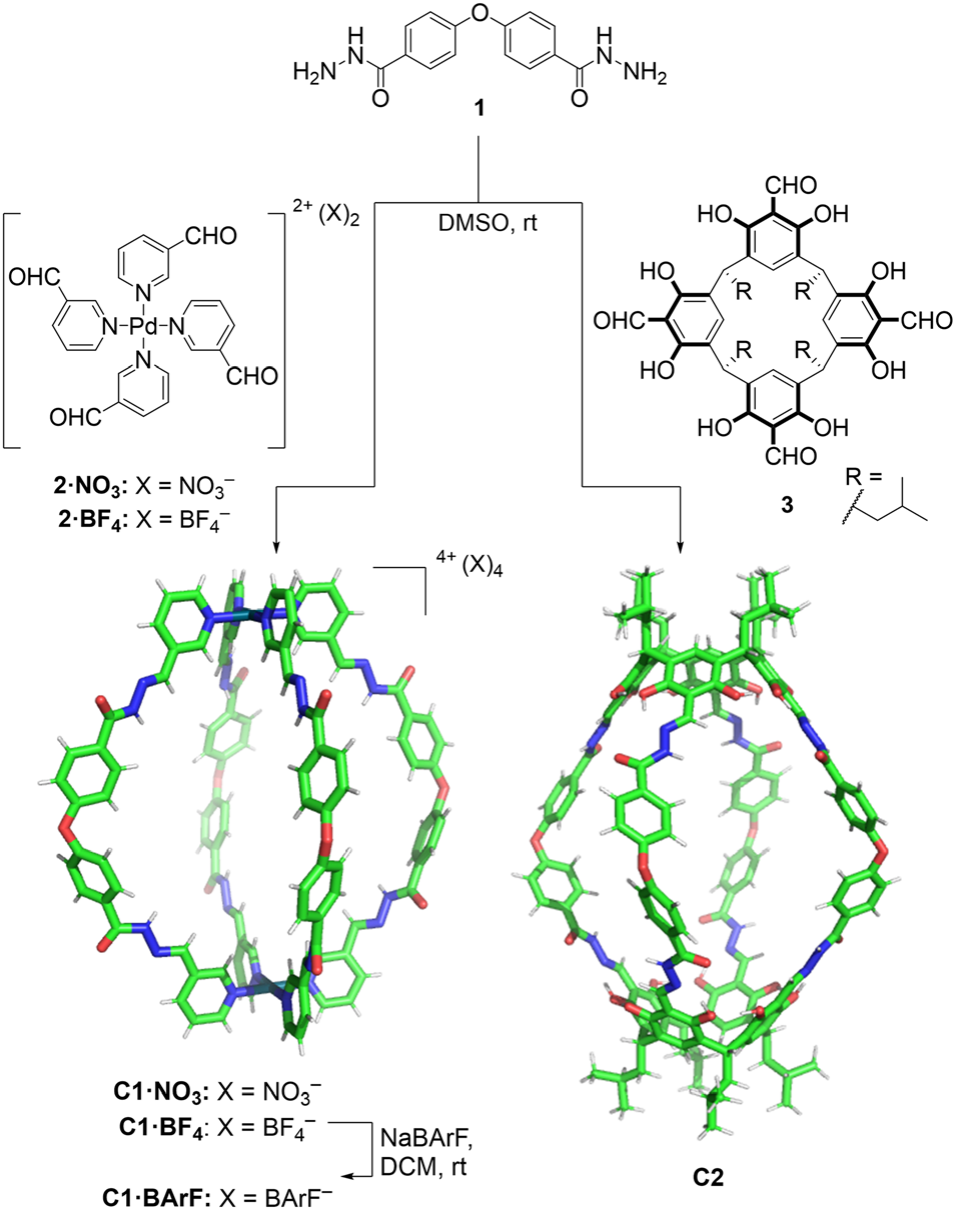
Self-assembly of molecular cages C1 and C2 by hydrazone bond formation. The figure shows X-ray crystal structure of C1·NO_3_ cage (counteranions removed for clarity) while the C2 cage structure was obtained from Spartan MMFF molecular modelling (see ESI† for X-ray and molecular modelling details).

Based on this idea, we designed two similarly shaped molecular cages (C1 and C2) by the self-assembly of 4 di-hydrazide ligands (1) with 2 tetra-aldehyde derivatives (2 or 3) (Fig. 2). Both cage forming reactions occur overnight at room temperature in DMSO. When ligand 1 is reacted with nicotinaldehyde–Pd complex 2, then Pd_2_L_4_ assembly C1 is obtained. The solubility of C1 can be controlled using different counteranions; the BArF cage C1·BArF, prepared *via* anion metathesis of C1·BF_4_, is soluble in organic solvents,^16^ whereas the nitrate cage C1·NO_3_, obtained directly from the reaction of 1 and 2·NO_3_, can dissolve in aqueous mixtures containing DMSO. Organic cage C2 was obtained by the reaction of 1 and calixarene starting material 3. This cage is soluble in both organic solvents and in aqueous mixtures containing DMSO. For both cages it is required typically 1–5% DMSO in water to achieve μM concentration. All cages were fully characterised by ^1^H, ^13^C, DOSY NMR, and HRMS (see ESI†).

Additionally, the formation of the molecular cage C1·NO_3_ was unambiguously confirmed by X-ray crystallography (Fig. 2, see ESI† for details). The crystal structure of the C1·NO_3_ cage shows a quasi-spherical geometry, with a Pd–Pd distance of 18.6 Å and a *trans* ether O–O separation of 19.9 Å. In this structure, all the hydrazone NH bonds are pointing into the cavity of the cage, providing potential sites for polar interactions with an encapsulated guest. While we have been unable to grow XRD quality crystals of C2, a molecular model (X-ray structure shown in Fig. 2, additional details are available in the ESI†) indicates that the size and shape of the organic cage is similar to C1; the distance between the centroids of the two calixarene motifs is 18.8 Å while the *trans* ether O atoms are separated by 19.7 Å.

### Host–guest chemistry

The affinity of the cages towards DOXO was assessed by means of titration experiments.^61^ The photophysical properties of DOXO, which has a fluorescence emission maximum at 590 nm (*λ*_exc_ = 470 nm), enabled the use of emission change to quantify encapsulation of the guest, which was also confirmed by ^1^H NMR studies. Both C1·NO_3_ and C2 are soluble in the range 20–100 μM in phosphate buffer (buffer concentration 100 μM) with 1–5% v/v DMSO. As the absorption spectra of the molecular cages and DOXO do not overlap (Fig. S32 and S33†), quantitative fluorescence titrations of DOXO with increasing amounts of molecular cages were performed. The titrations with both C1·NO_3_ and C2 show a DOXO fluorescence emission decrease (Fig. S28 and S29†). This is attributed to the presence of electron-poor Pd[(pyridine)_4_]^2+^ moieties in cage C1·NO_3_ and electron-rich resorcinol rings in cage C2, that quench the DOXO emission. The quenching can take place by intermolecular photo-induced electron transfer (PET) process as we estimated using the Rehm–Weller equation (see ESI†).^62,63^

**Fig. 3:**
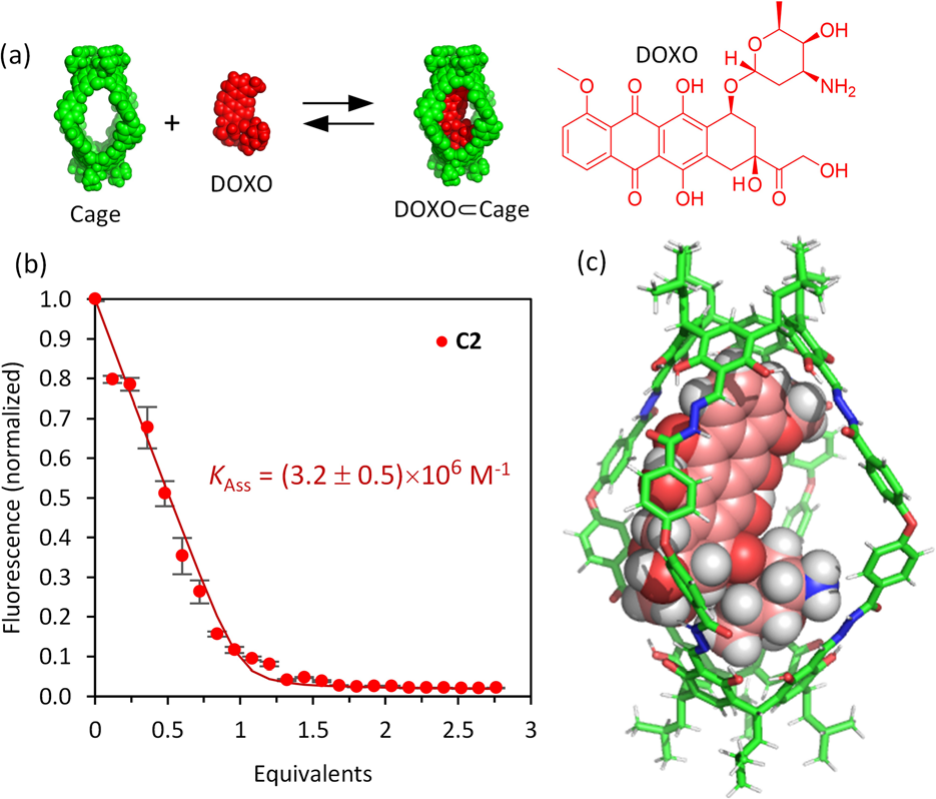
Fluorescence titration experiments. (a) Schematic representation of drug encapsulation; (b) binding data for the encapsulation of DOXO by cage C2 obtained from the fluorescence spectra for the addition of C2 to DOXO (50 μM) in 100 μM phosphate buffer, pH 7.2. The solid points are experimental data, and the continuous lines is the fitted binding isotherm for a 1 : 1 host–guest model, obtained as the average of 4 independent titrations (vertical bars show the standard error for each point). Fluorescence measured at 590 nm with *λ*_exc_ = 470 nm (phosphate buffer 100 μM/DMSO 0.25–2.0%, pH 7.2); (c) MMFF molecular model of the supramolecular complex [DOXO⊂C2] obtained with the Spartan'20 software.

The fluorescence quenching experiments reveal a significant difference between cages C1·NO_3_ and C2. The decrease in fluorescence emission was much more noticeable for C2 compared to C1·NO_3_, showing more efficient fluorescence quenching. Fitting the emission intensity *versus* the concentration of added cage to a 1 : 1 binding isotherm^64^ using R and RStudio,^65,66^ we determined an association constant of 3.2 × 10^6^ M^−1^ for the organic cage C2 (Fig. 3). For cage C1·NO_3_, we estimated an association constant in the order of magnitude of 10^4^ M^−1^ with low reproducibility, likely associated to cage disassembly by complexation of Pd^2+^ by the NH_2_ moiety of DOXO as observed by NMR titrations (Fig. S24 and S28†). The increase of affinity is likely associated with the more hydrophobic nature of uncharged cage C2 in contrast to the tetracationic C1·NO_3_.^67^ The inward facing NH groups of C1·NO_3_, as determined from the X-ray crystal structure, could also lead to an energetically more favourable hydration of the cavity, leading to poorer DOXO binding. The high association constant of DOXO with C2 permitted a Job's plot analysis to confirm the 1 : 1 stoichiometry of [DOXO⊂C2] (see ESI Fig. S30†), which is also supported by molecular modelling, suggesting that DOXO fits into the cavity of cage C2 (Fig. 3c, see ESI† for molecular modelling details).

To check cavity occupancy by the guest, the cavity volume of both cages was determined using the CageCavityCalc Python script. The calculations gave 1350 Å^3^ for cage C1 and 1500 Å^3^ for cage C2, showing that both cages have a similar cavity space.^68^ As the size of DOXO is 498 Å^3^, the single guest occupancy for C1 and C2 would be 37% and 33%, respectively, significantly less than the optimal 55% predicted by Rebek for closed-shell organic hosts.^69^ Therefore, the binding observed does not follow the 55%-rule as cages C1 and C2 have very large portals, differing from the closed-shell hosts used to develop the 55%-rule.^15^

**Fig. 4:**
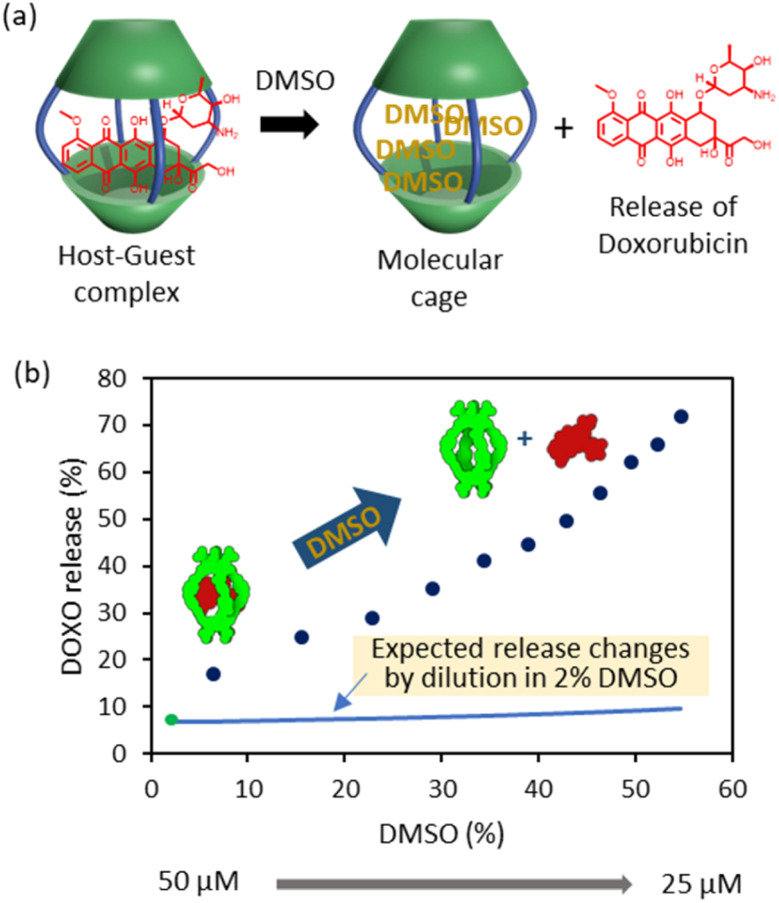
DOXO release experiments. (a) Schematic representation of DOXO release by competition with DMSO molecules. (b) Plot of recovery of non-bound DOXO *versus* percentage of added DMSO (6–55%) to the solution containing C2 (*C*_initial_ = 50 μM, *C*_final_ = 25 μM) and DOXO (*C*_initial_ = 50 μM, *C*_final_ = 25 μM) in phosphate buffer (1 mM, pH 7.2). The green dot indicates the release at 2% DMSO as determined in the binding experiments of Fig. 3. The blue line indicates the expected release changes by dilution from 50 μM to 25 μM in a solution containing 2% DMSO.

It is also possible to trigger the decomplexation of [DOXO⊂C2] by adding increasing amounts of DMSO (Fig. 4a). This causes the fluorescence intensity to increase, presumably because the exclusion of DOXO reduces quenching. From this data, we estimated that *ca.* 70% DOXO is released when the solution reaches 55% v/v DMSO (Fig. 4b). The decrease in affinity is likely attributed to the weaking of the hydrophobic effect and competition of DMSO molecules with DOXO for the cavity of the cage.

^1^H NMR titration experiments have also been undertaken to further corroborate the formation of the host–guest [DOXO⊂cage] complexes. At the cage concentration needed for NMR experiments (≈0.5–1 mM), it was found that both C1 and C2 are not soluble enough in D_2_O to make a meaningful comparison to the fluorescence titrations. Also considering that the use of organic solvents is much less relevant to binding under biological conditions, no attempt was made to quantify encapsulation by NMR. Nonetheless, the changes in individual chemical shifts provide localised information that is not provided from emission data. From these experiments we observed small chemical shift changes consistent with binding (see ESI Section 4 and Fig. S24–S26†). Remarkably, it was observed an upfield shift of the inward-facing protons of the cages evidencing the encapsulation of DOXO inside the cavity of both C1 and C2 cages. Similar observations of small chemical shift changes and large fluorescence changes upon anticancer drug encapsulation have been described in similar works.^51^

### Cage disassembly experiments in mixed aqueous solution

**Fig. 5:**
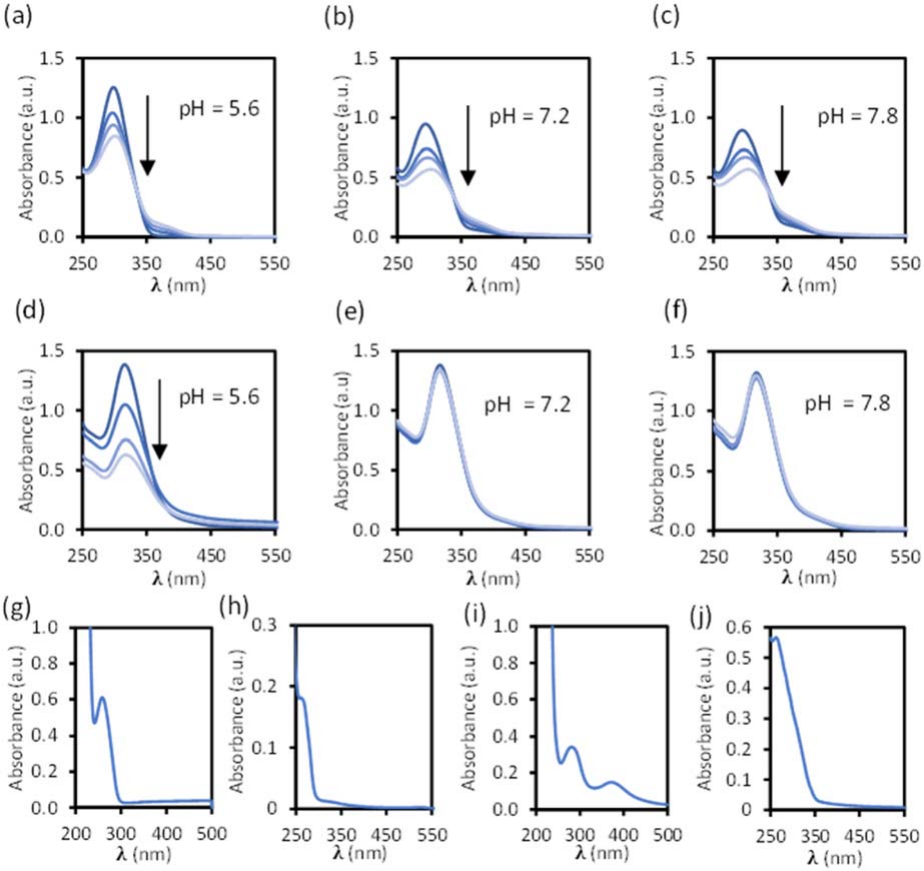
Stability of cages C1·NO_3_ (10 μM, (a–c) 0, 1, 2, 4 days) and C2 (10 μM, (d–f) 0, 1, 2, and 3 days) at different pH values over time in a buffered solution with phosphate (100 μM) and 1% DMSO. Absorbance spectra of building block components (pH 7.1 buffered solution with phosphate (100 μM) with 5% DMSO): (g) linker 1 (25 μM). (h) Pd(ii) complex 2·NO_3_ (50 μM). (i) Cavitand 3 (25 μM). (j) Ligand S2 (20 μM).

**Fig. 6:**
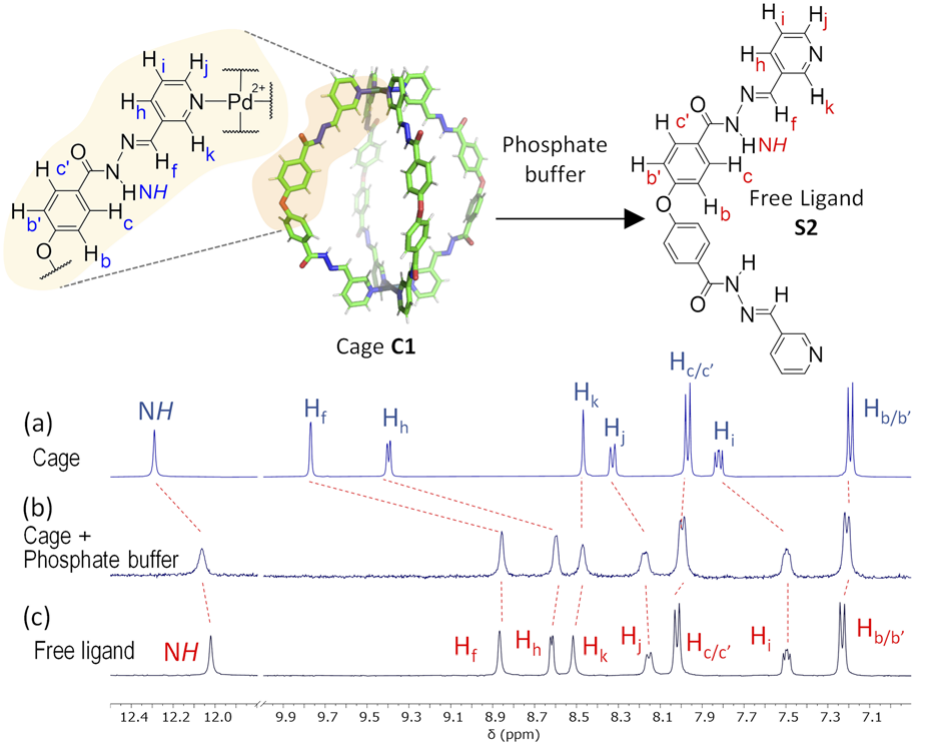
^1^H NMR disassembly experiment of C1·NO_3_ in DMSO-*d*_6_ (0.5 mM, 550 μL) with phosphate buffer (pH 7.2, 100 mM, 50 μL). (a) ^1^H NMR spectra of cage C1·NO_3_. (b) ^1^H NMR spectrum after the addition of phosphate buffer (*ca.* 5 min), causing cage disassembly. (c) ^1^H NMR spectra of free ligand (see structure S2 in ESI†).

Disassembly experiments of cages C1 and C2 were performed at different pH values (pH 5.6, 7.2, and 7.8) in DMSO/phosphate buffer. This process was initially studied by UV-visible spectroscopy by monitoring the decrease in the absorption band centred at 300–320 nm characteristic of each cage, and not present in the building block components (see ESI†) as a function of time (Fig. 6). The UV-visible spectra of molecular cage C1·NO_3_ shows a decrease in absorbance over several days at all of the three different pH values 5.6, 7.2, and 7.8 (Fig. 5a–c). In contrast, the UV-visible spectra of molecular cage C2 remained unchanged over time (0–3 days) under neutral and basic conditions (Fig. 5e and f) but showed a decrease in absorbance at pH 5.6 (Fig. 5d). These results would suggest that C1·NO_3_ and C2 disassemble *via* different mechanisms; the metallo-organic cage is likely degraded by coordination of the phosphate buffer anion to the palladium (see ^1^H NMR experiments below), resulting in ligand displacement, while the pure organic cage dissociates through acid-catalysed hydrolysis of the hydrazone bonds. The time scale of the hydrolysis is relevant in a biological context as the sustained delivery of drugs over several days is a desirable property in anticancer drug delivery.^70^

Further evidence for a ligand displacement process was obtained by recording the ^1^H NMR spectra of C1·NO_3_ in DMSO-*d*_6_ before and after the addition of phosphate buffer after *ca.* 5 min (Fig. 6). Addition of buffer results in upfield shifting of the pyridyl moiety protons (H_f_, H_i_, H_j_, H_k_), which is in agreement with the loss of the inductive effect associated with coordination to Pd(ii). Furthermore, a comparison to the ^1^H NMR spectra of free ligand clearly indicates that this species is released intact without hydrolysis of the hydrazone bond. The fast disassembly observed in the NMR experiments (in *ca.* 5 min) is associated to the larger concentration of buffer with respect to cage, as we observed a slower kinetics reducing the buffer concentration (see ESI†). Unfortunately, the low solubility of cage C2 at the required concentrations for NMR, as well as the broad features of these spectra in phosphate buffer/DMSO have hindered a similar study.

Overall, these stability experiments highlight some of the challenges in using metallo-organic cages for bio-medical applications.^6^ They also show that pure organic cages may be better suited to this purpose. Moreover, the selective hydrolysis of the hydrazone bonds in C2 at slightly acidic pH presents an ideal scenario for stimuli responsive drug delivery at the acidic microenvironment of a tumor.

### Biological studies

**Fig. 7:**
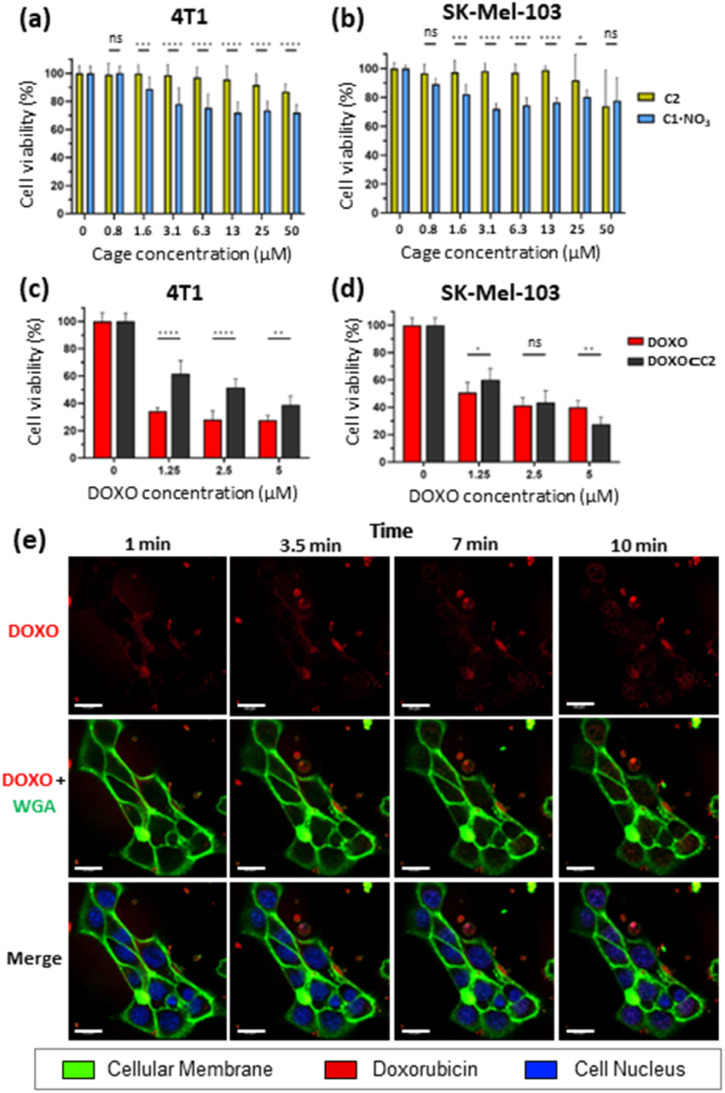
Cell viability measured by WST-1. Data represented as mean ± SEM (*n* = 3). Toxicity assay with organic cage C2 (yellow) and palladium metallo-organic cage C1·NO_3_ (blue) on: (a) 4T1 cells and (b) SK-Mel-103 cells. Treatment with DOXO⊂C2 complex (black) and free doxorubicin (red) on: (c) 4T1 cells and (d) SK-Mel-103 cells. Increasing concentrations of DOXO and a fixed concentration of organic cage C2 (25 μM) was used to obtain 95% encapsulation of DOXO. (e) Time-lapse confocal images of SK-Mel-103 cells incubated with Hoechst (blue nuclei marker), WGA (green membrane marker) and treated with the DOXO⊂C2 complex (fluorescent red) at 5 μM up to 10 min. Scale bar represents 20 μm.

Toxicity studies were first performed to determine the biocompatibility of the empty cages. Tumoral 4T1 (murine triple-negative breast cancer) and human melanoma SK-Mel-103 cell lines were treated with increasing concentrations of C1·NO_3_ and C2 and cell viability was measured at 48 h (Fig. 7). The C1·NO_3_ palladium cage produced toxicity at low concentrations (viability lower than 80% was found at concentration of the cage of 3.12 μM in agreement with previous works on metallo-organic cages),^40,71^ whereas the organic cage C2 was shown to be non-toxic at concentrations as high as 25–50 μM. We also examined that the building blocks components of cage C2 (ligand 1 and calixarene 3) are non-toxic (Fig. S37a in ESI†). Selecting the less toxic organic cage C2 for encapsulation studies, when cancer cells were treated with the [DOXO⊂C2] cage, an antitumoral effect was achieved in 4T1 and SK-Mel-103 cells attributed to cage internalisation and cage disassembly in the lysosomes. This is supported by comparing cells treated with the building blocks components of cage C2 (Ligand 1 and calixarene 3) and DOXO (1 + 3 + DOXO) with cells treated with [DOXO⊂C2]. In both cases, [DOXO⊂C2], and 1 + 3 + DOXO, a comparable antitumoral effect is achieved indicating that the efficacy of DOXO remains unaffected by the C2 framework or [DOXO⊂C2] formation (Fig. 7c, d and S37b in ESI†). Additionally, the treatment with free DOXO resulted in a slightly enhanced antitumoral effect. This can be attributed to the efficient entrapment of the free drug, leading to differential internalisation and processing of [DOXO⊂C2] by cancer cells (*vide infra* and Fig. 7c and d). Nevertheless, the results indicate that the activity of DOXO is fully recovered upon its release from [DOXO⊂C2]. Overall, these results highlights the suitability of the organic cage for drug delivery and the low toxicity of the unloaded organic cage compared to the metallo-organic cage.

To assess the internalisation of the molecular cages, cellular uptake studies of the doxorubicin-encapsulated organic cage were focused on SK-Mel-103 cells. Time-lapse confocal images revealed an increase in DOXO fluorescence (in red) within the cytoplasm of cells (limited by membrane marker in green) that finally reaches the nuclei (in blue) of the cells (Fig. 7). The rapid internalisation, within minutes, suggests passive diffusion as a mechanism of cellular uptake. Although, membrane permeability is typically limited to molecules with sizes of 1500 Å^3^,^72–75^ (cage C2 has a molecular volume of 2600 Å^3^ just slightly over the limit) several works have demonstrated that cages of various sizes can cross cell membranes and deliver anticancer drugs in cells.^37,41,42,45,48,53,76,77^ Analogous time-dependent confocal images with free DOXO confirmed the different drug-internalisation ratio when compared with [DOXO⊂C2] (Fig. S38 in ESI†). The images showed a faster diffusion of DOXO from cellular medium to the cells, with a large amount accumulated in the nucleus after 10 minutes compared to [DOXO⊂C2]. Overall, our studies indicate the proper formation of [DOXO⊂C2] cage and ability to enter in cells and deliver DOXO.

## Conclusions

In this work we have compared a similarly shaped metallo-organic cage and a fully organic cage. We found that the organic cage C2 is a promising drug delivery system, with clear advantages over the analogue the palladium cage C1. Both cages are water-soluble requiring only a 1–5% of DMSO to achieve micromolar concentrations, which is compatible with *in vitro* cell culture experiments. The organic cage C2 remains intact in neutral pH phosphate buffer, unlike the palladium cage C1, which breaks down under similar conditions. Cage C2 has a high binding affinity towards the anticancer drug doxorubicin (DOXO), allowing efficient encapsulation at the micromolar concentration required for biological drug delivery. In contrast, C1·NO_3_ exhibits weaker binding and disassembly by interaction with DOXO, lacking sufficient binding affinity and stability. In addition, cage disassembly takes place when cage C2 is placed in a slightly acidic pH, making the cage an ideal pH-responsive drug delivery system. In addition to the excellent chemical properties, cage C2 exhibits a remarkable cellular compatibility. Cage C2 does not shows any cellular toxicity even at high doses (25–50 μM), in contrast to the significant toxicity observed in C1. Additionally, cage C2 effectively delivers DOXO to cells from the DOXO⊂C2 complex, preserving the cytotoxic activity of this anticancer drug. Overall, the organic cage C2 is a proof-of-concept low toxicity drug delivery system that shows an excellent performance in cells. These results highlight the possibility of using pure organic molecular cages as suitable drug-delivery systems due to their absence of toxicity compared to metalloorganic cages.

## Data availability

Data associated to the manuscript is available as ESI† in PDF format and the crystal structure data in CIF format.

## Author contributions

The manuscript was written through contributions of all authors. All authors have given approval to the final version of the manuscript.

## Conflicts of interest

There are no conflicts to declare.

## Supplementary Material

SC-015-D4SC02294G-s001

SC-015-D4SC02294G-s002
