# Supplementary material for: Comparing organic and metallo-organic hydrazone molecular cages as potential carriers for doxorubicin delivery
Source: Chem Sci. 2024 Jun 7;15(26):10010–7. doi: 10.1039/d4sc02294g (PMC11220577; doi:10.1039/d4sc02294g)
Supplement: SC-015-D4SC02294G-s001 [file SC-015-D4SC02294G-s001.pdf]

Electronic Supplementary Material (ESI) for Chemical Science.
This journal is © The Royal Society of Chemistry 2024

SUPPORTING INFORMATION

Contents of the Supporting Information

1. General equipment S3

2. Synthesis of ligands and cages S4

3. X-Ray crystallographic data for **C1**·NO_3 .... S25

4. NMR spectrometric host-guest experiments and cage disassembly S27

5. Spectrophotometric experiments S31

6. Rehm–Weller calculations S35

7. Molecular Modelling S36

8. Cell viability studies S40

1. General equipment

NMR Experiments. ^{1}H, ^{13}C and ^{19}F NMR spectra were recorded on a Bruker FT-NMR Avance 400 (Ettlingen, Germany) spectrometer at 300K, using TMS as an internal standard or a Bruker Neo500 spectrometer, with a 500 MHz Bruker (11.7 T) standard mouth (54mm) shielded magnet and two radio frequency channels, a magnetic field gradients unit equipped with BBOF Plus ATM.1H/BB/19F direct multinuclear probe (5mm) with field gradients on the z-axis and automatic frequency control, 1H channel, wideband channel (5N to 31P and direct observation of 19F), BBI. 1H/BB/ inverse probe (5mm) with field gradients on the z-axis. Wideband channel frequency interval of 5N to 31P. BCUII, accessory for air temperature control up to -40ºC. Liquid nitrogen evaporator for experiments at lower temperatures. Chemical shifts (δ) are reported in parts per million (ppm) and referenced to residual solvent. Coupling constants (*J*) are reported in hertz (Hz). Standard abbreviations indicating multiplicity are used as follows: *s* = singlet, *d* = doublet, *t* = triplet, *q* = quartet, *m* = multiplet.

High resolution Mass Spectrometry. HR-ESI-MS experiments were performed using a SYNAPT XS high-definition mass spectrometer (Waters Corporation, Manchester, UK) equipped with an electrospray ionization (ESI) source. Capillary voltage was set to 1.5 kV operated in the positive ionization mode and in the resolution mode. Source settings were adjusted to keep intact the molecular cages. Typical values were cone voltage 20−40 V and source offset 4 V; source and desolvation temperatures were set to 110 and 350 °C, respectively. Cone and desolvation gas flows were 150 and 500 (L/h), respectively. Characterisation of molecular cages were performed by MALDI-TOF/TOF mass spectrometry experiments using a TIMS-TOFF Flex (Bruker) in MALDI operation, in reflector positive mode at 1000-5800 m/z rang and a laser intensity of 60 %. The analyses were performed in the mass spectrometry and proteomics facilities of SCSIE University of Valencia.

Absorption and emission Spectrometric measurements. Optical extinction spectra were recorded using a JASCO V-650 UV/vis spectrophotometer with a Single monochromator, 1200 lines/mm plane grating, Czerny-Turner mount, Double-beam equipped with a deuterium lamp (190 to 350 nm) and a Halogen lamp (330 to 900 nm), and wavelength range of 190 to 900 nm. Fluorescence spectroscopy was carried out on a JASCO FP- 8300 spectrofluorometer (Hitachi High Technologies) with high resolution of 1.0 nm and a wavelength range of 200 to 750 nm. Titration experiments have been carried out with a Perkin Elmer EnSpire 2300 Multimode Plate Reader equipped with fluorescence (monochromator wavelength range of 230 to 850 nm with an excitation and emission bandwidth of 5 nm), absorbance (monochromator wavelength range of 230 to 1000 nm with monochromator bandwidth of 5 nm) and luminescence detection.

2. Synthesis of ligands and cages

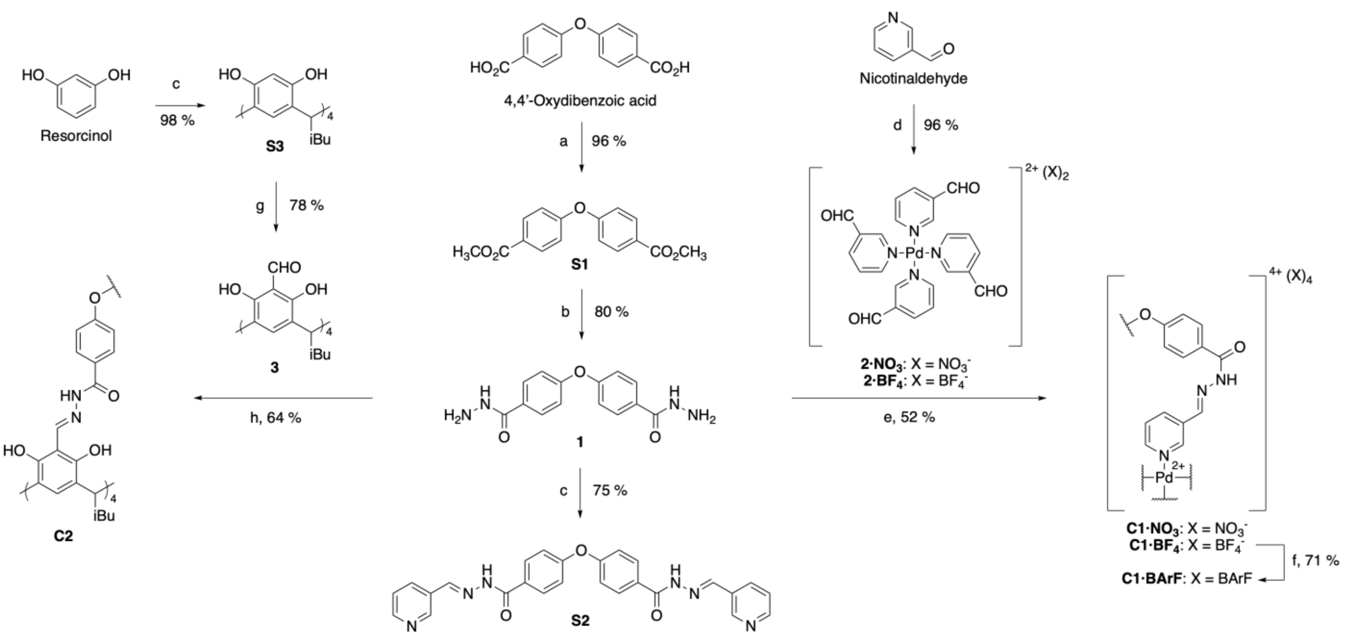

Figure S1. Synthesis of molecular cages **C1** and **C2**. Reagents and conditions: (a) Catalytic H_2SO_4, methanol, 70 ºC, overnight; (b) $N_2H_4 \cdot H_2O$, methanol/toluene (1:1), 70 ºC, 24 h; (c) Nicotinaldehyde, molecular sieves of 3 Å, DMSO, rt, 18 h; (d) $Pd(NO_3)_2$ or $Pd(CH_3CN)_4(BF_4)_2$, acetonitrile, room temperature, overnight; (e) 4,4'-oxydi(benzohydrazide) (**1**), molecular sieves of 3 Å, DMSO, room temperature, overnight; (f) NaBArF, DCM, sonication, 30 min; (g) Isovaleraldehyde, catalytic HCl, ethanol, 0 ºC to 85 ºC, 24 h; (h) i. Hexamethylenetetramine, TFA, 85 ºC, 24 h; ii. Aqueous solution of HCl 1M, room temperature, 24 h.

Compounds **1**,[S1] **2·NO3**,[S2] **2·BF4**,[S3] **3**,[S4] **S3**[S5] were prepared according to literature procedures.

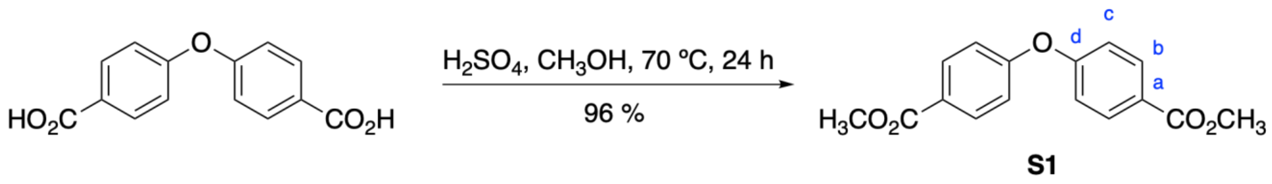

Synthesis of dimethyl 4,4'-oxydibenzoate (S1): S1 was prepared by a modification of reported synthetic procedures.[S6,S7,S8] 4,4'-oxydibenzoic acid (4.0 g, 15,50 mmol) was dissolved in anhydrous methanol (70 mL) under N_2 atmosphere, and 1.0 mL of concentrated H_2SO_4 was added. The resulting solution was heated at 70 ºC over 24 h to obtain abundant white precipitate. The mixture was cooled down, vacuum filtered and washed with cold methanol to obtain **S1** as a pure white solid (4.3 g, 96%).

^{1}H NMR (400 MHz, Acetone-d$_6$) δ 3.88 (6H, *s*, CO_2CH_3), 7.18 (4H, *d*, J = 8.9 Hz, b), 8.07 (4H, *d*, J = 8.8 Hz, c).

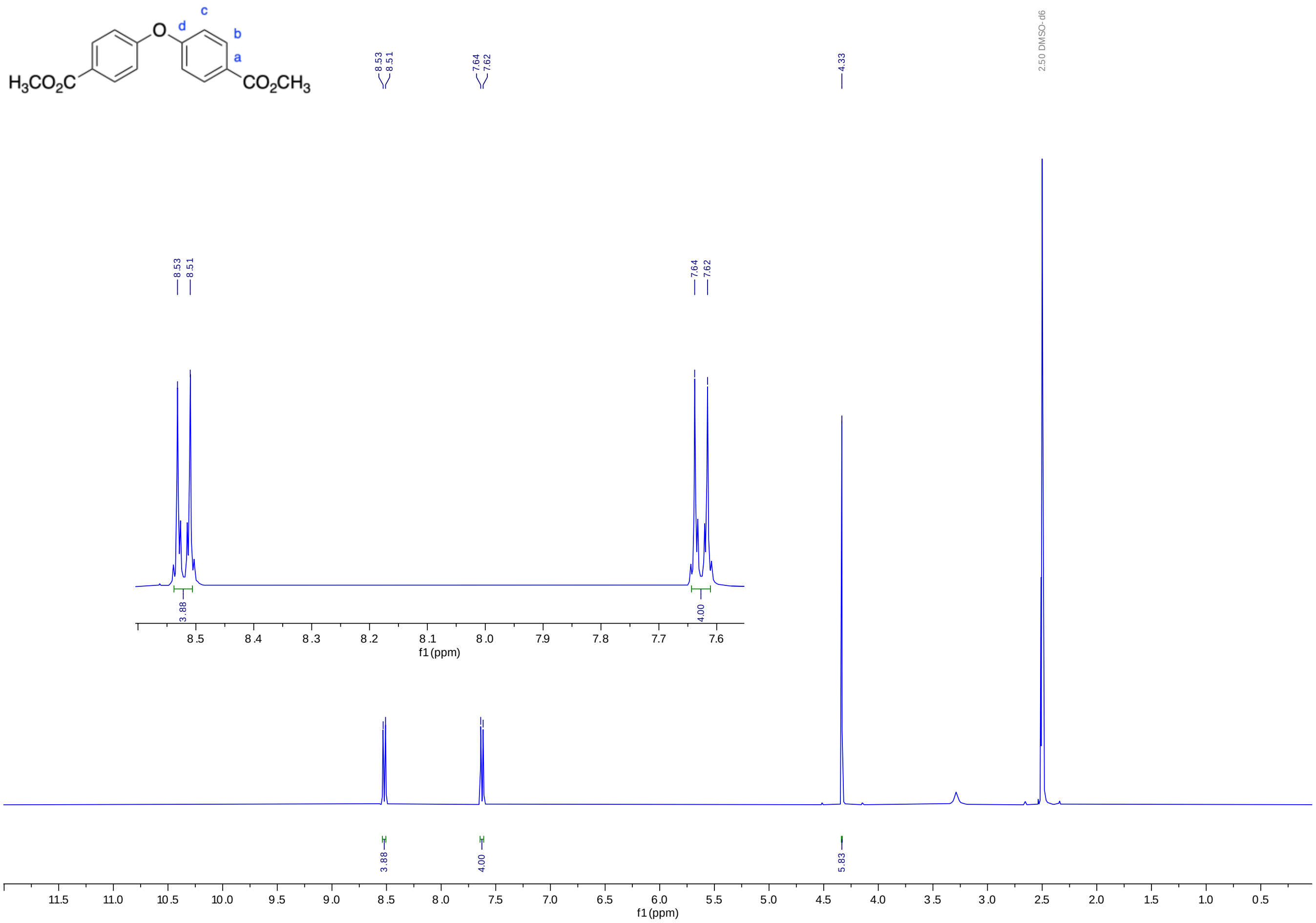

Figure S2. ^{1}H NMR (400MHz, Acetone-d_6) of compound **S1**.

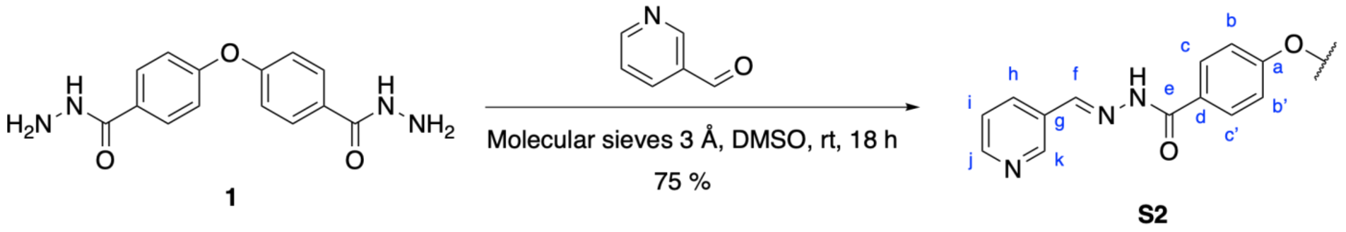

Synthesis of *N'*-((*E*)-pyridin-3-ylmethylene)-4-(4-(2-(((*E*)-pyridin-3-ylmethylene)amino)acetylphenoxy)benzohydrazide (S2). To a solution of **1** (50.0 mg, 174.6 µmol) in 1.0 mL of DMSO with molecular sieves of 3 Å were added 50 µL of nicotinaldehyde (532.6 µmol). The resulting solution was stirred at room temperature over 18 h. To the reaction mixture were added 10 mL of ethyl acetate, the resulting precipitate was isolated by centrifugation at 7,000 rpm over 2 minutes and filtered to obtain **S2** as a white powder (37,5 mg, 75%).

^{1}H NMR (600 MHz, DMSO-d$_6$) δ 7.23 (2H, *d*, *J* = 8.2 Hz, H-b and H-b'), 7.50 (1H, *dd*, *J* = 4.8, 8.0 Hz H-i), 8.02 (2H, *d*, *J* = 8.3 Hz, H-c and H-c'), 8.16 (1H, *d*, *J* = 7.5 Hz, H-j), 8.52 (1H, *s*, H-k), 8.57-8.80 (1H, *m*, H-h), 8.87 (1H, *s*, H-f), 12.02 (1H, *s*, N*H*).

^{13}C NMR (126 MHz, DMSO-d$_6$) δ 119.3 (C-b and C-b'), 124.5 (C-i), 129.2 (C-g), 130.7 (C-c and C-c'), 134.0 (C-j), 145.3 (C-k)148.7 (C-f), 151.2 (C-e).

HRMS (ESI) *m/z* $[M + H]^+$ calculated for $[C_{26}H_{21}N_6O_3]^+$ 465.1670, found 465,1658.

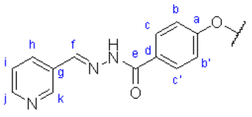

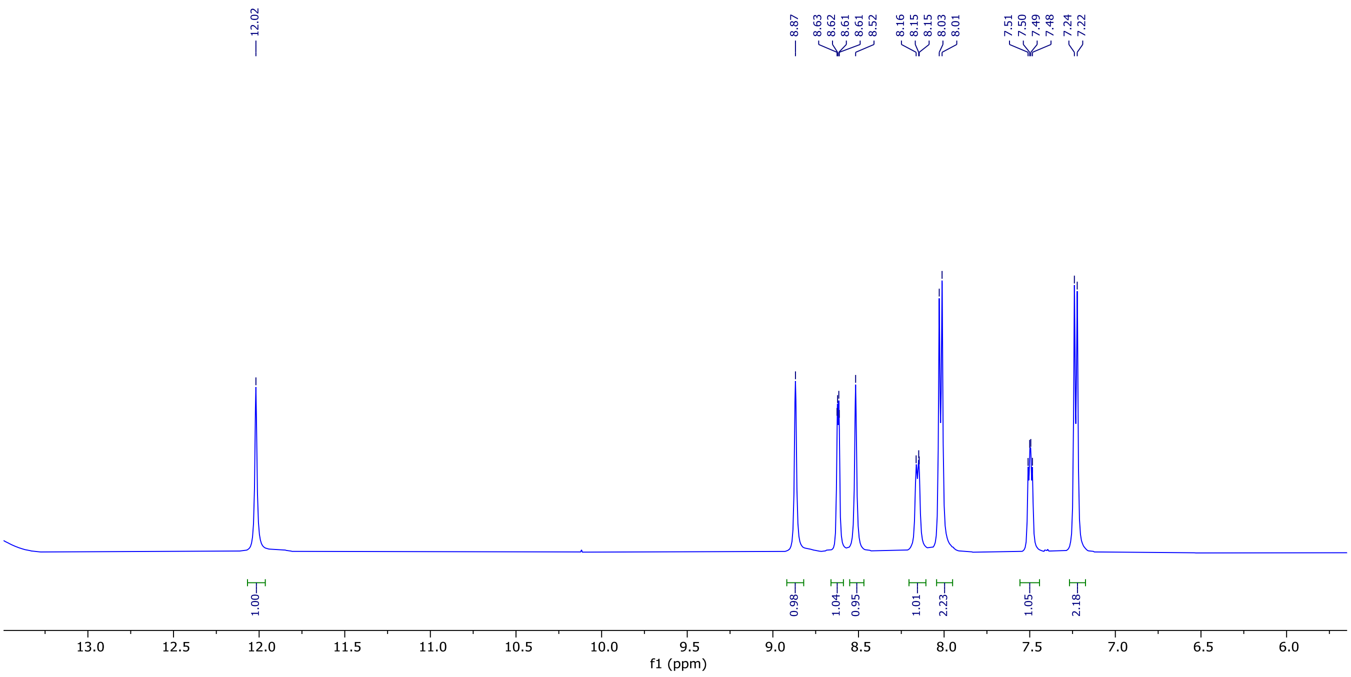

Figure S3. ^{1}H NMR (500 MHz, DMSO-d_6) of compound **S2**.

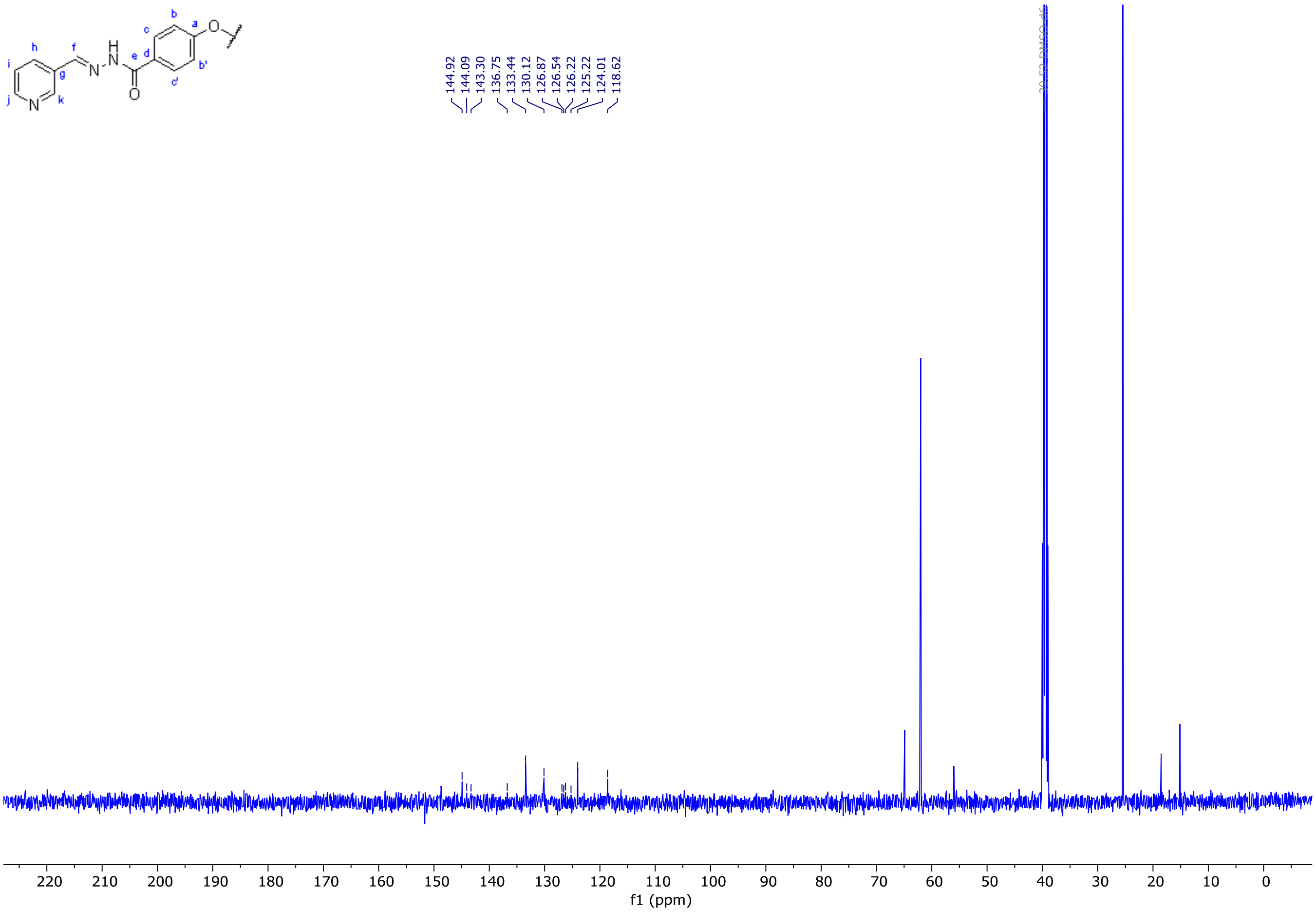

Figure S4. ^{13}C NMR (126MHz, DMSO-d_6) of compound **S2**.

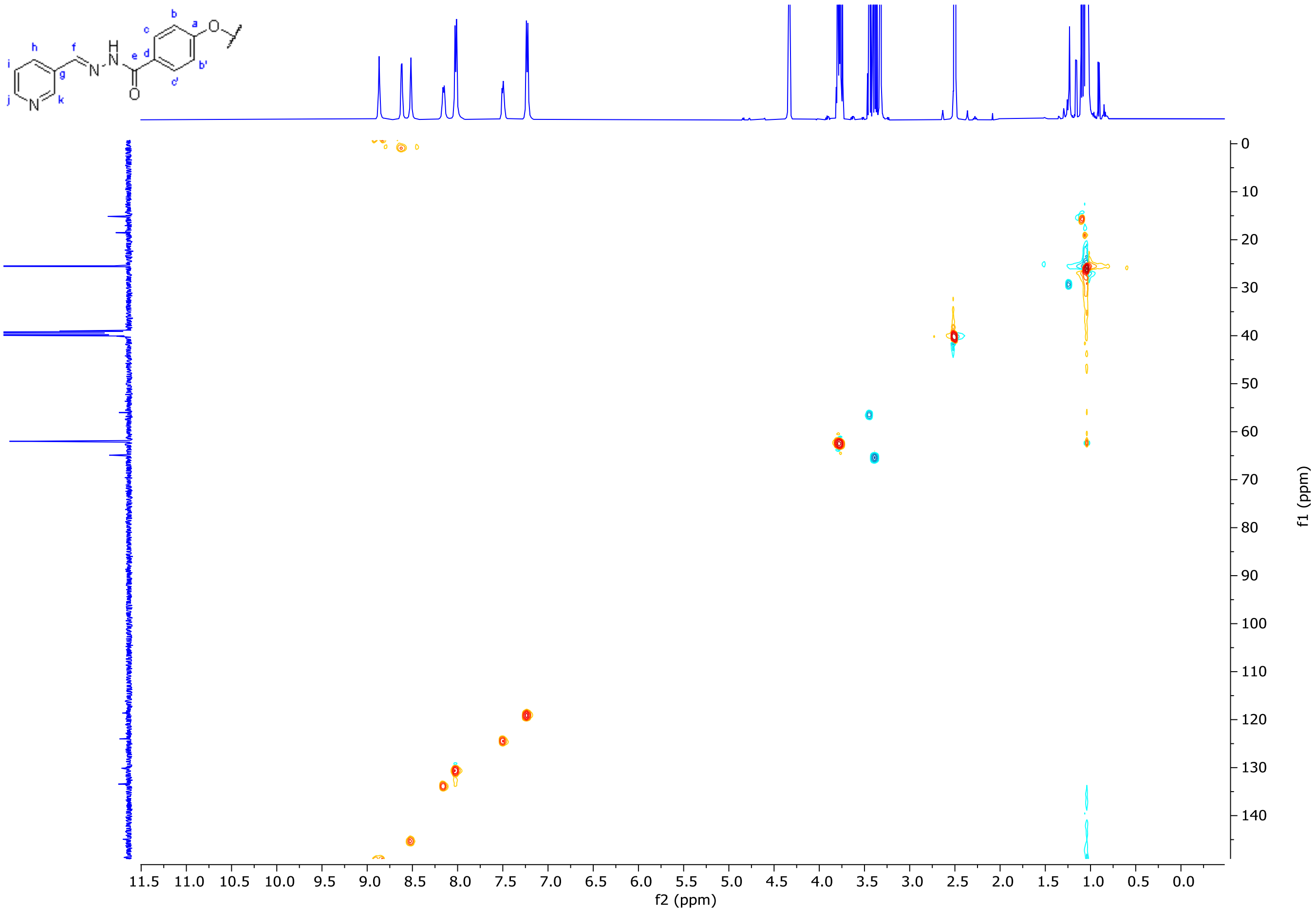

Figure S5. HSQC NMR (500MHz, DMSO-d_6) of compound **S2**.

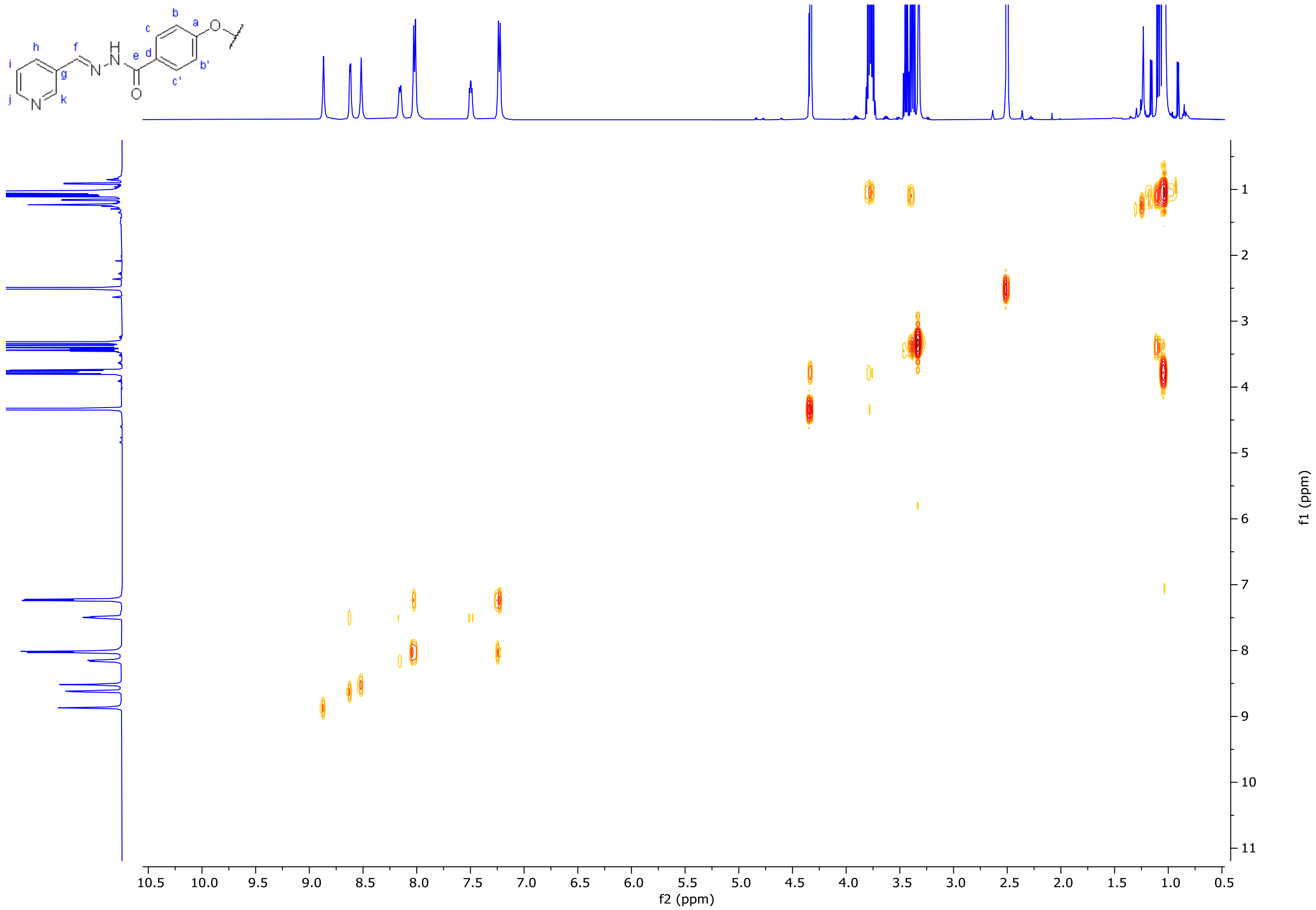

Figure S6. COSY NMR (500MHz, DMSO-d_6) of compound **S2**.

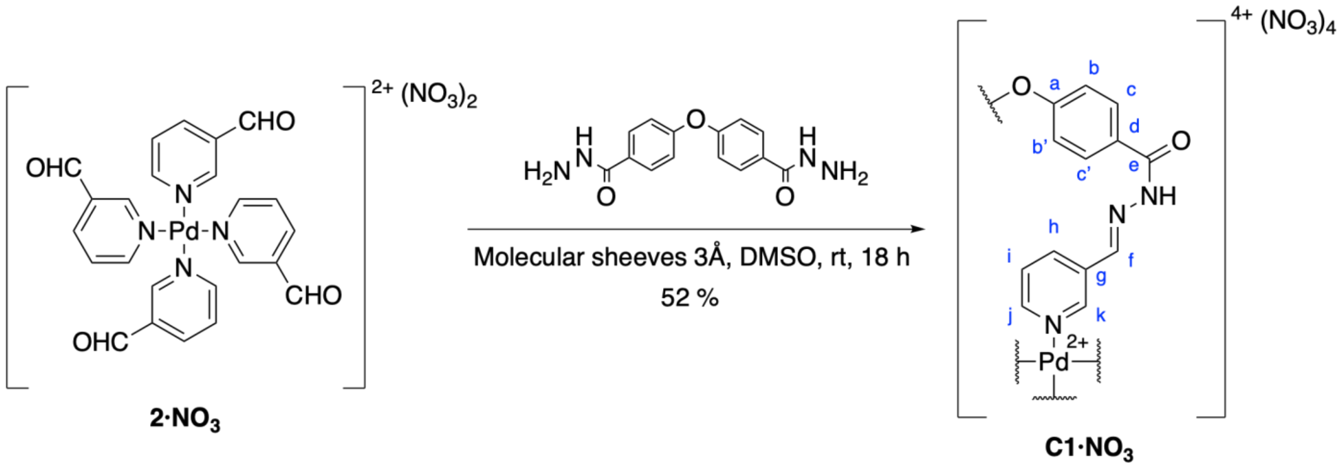

Synthesis of the Pd (II) Molecular Cage with NO_3^- counterion (C1·NO_3). 2·NO_3 (100 mg, 151.8 µmol) and **1** (86.9 mg, 303.7 µmol) were completely dissolved in 30 mL of anhydrous DMSO. The resulting solution was stirred at room temperature for 18 h, and then slowly poured into DCM (70 mL) until the formation of abundant white precipitate. The suspension was centrifuged at 10,000 rpm for 10 minutes,

the supernatant solution was decanted, and the precipitate was dried out to obtain **C1·NO3** pure as a white-grey solid (162 mg, 52 %).

^{1}H NMR (400 MHz, DMSO-d$_6$) δ 7.19 (2H, *d*, *J* = 8.8 Hz, H-b and H-b'), 7.82 (1H, *dd*, *J* = 8.0, 5.8 Hz, H-i), 7.97 (2H, *d*, *J* = 8.8 Hz, H-c and H-c'), 8.33 (1H, *d*, *J* = 8.8 Hz, H-j), 8.46 (1H, *s*, H-k), 9.39 (1H, *d*, *J* = 6.5, H-h), 9.77 (1H, *m*, H-f), 12.29 (1H, *s*, N*H*).

^{13}C NMR (126 MHz, DMSO-d$_6$) δ 118.8 (C-b and C-b'), 127.4 (C-g), 128.6 (C-a), 130.2 (C-c and C-c'), 133.7 (C-i), 138.5 (C-j), 143.1 (C-k), 149.0 (C-f), 151.3 (C-h), 159.2 (C-d), 162.7 (C-e).

HRMS (ESI) *m/z* [**C-1** + NO_3]$^{3+}$ calculated for $C_{104}H_{80}N_{28}O_{24}Pd_2$ 710.14, found 710.84.

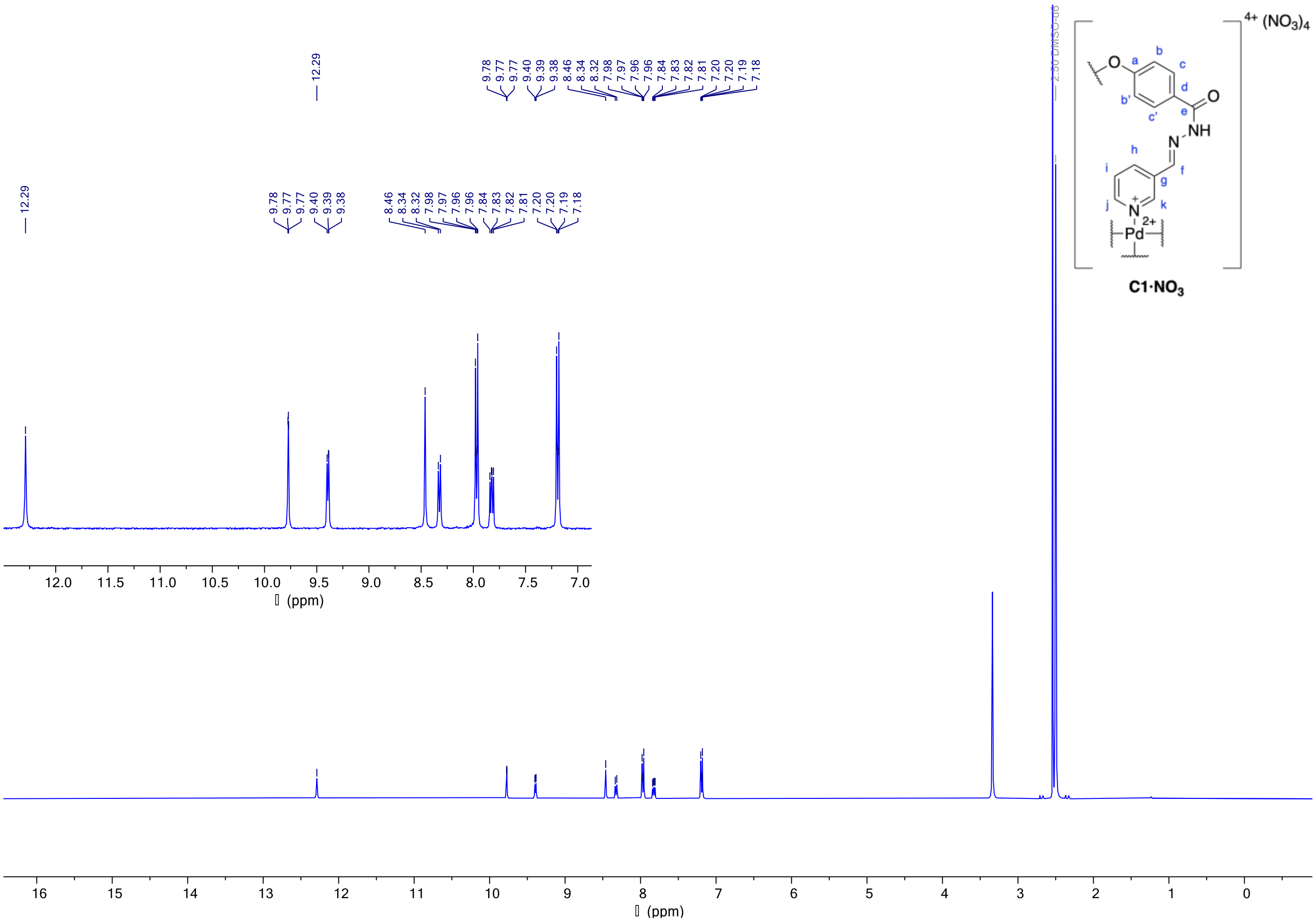

Figure S7. ^{1}H NMR (400MHz, DMSO-d_6) of compound **C1·NO3**.

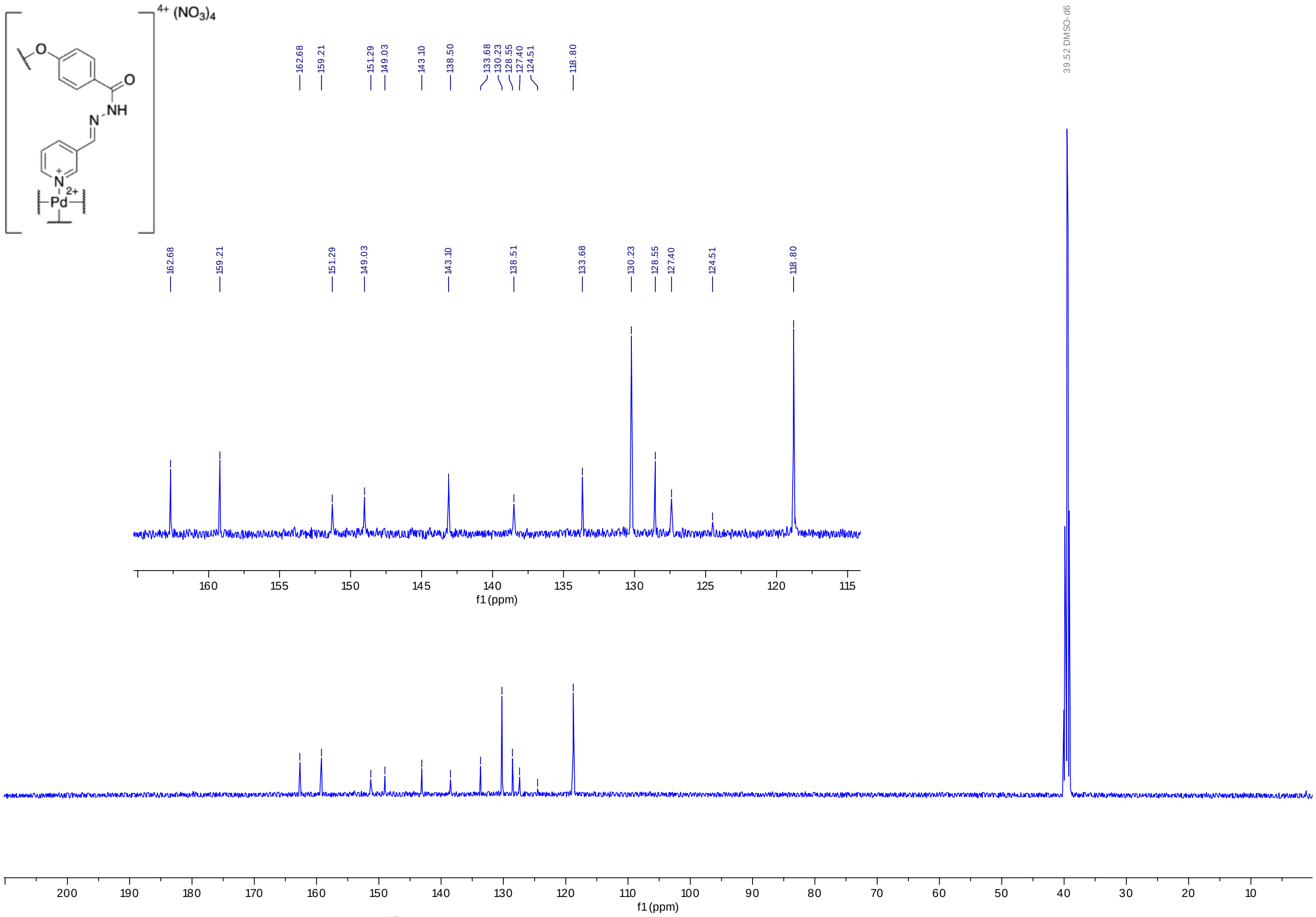

Figure S8. ^{13}C NMR (126MHz, DMSO-d_6) of compound **C1·NO3**.

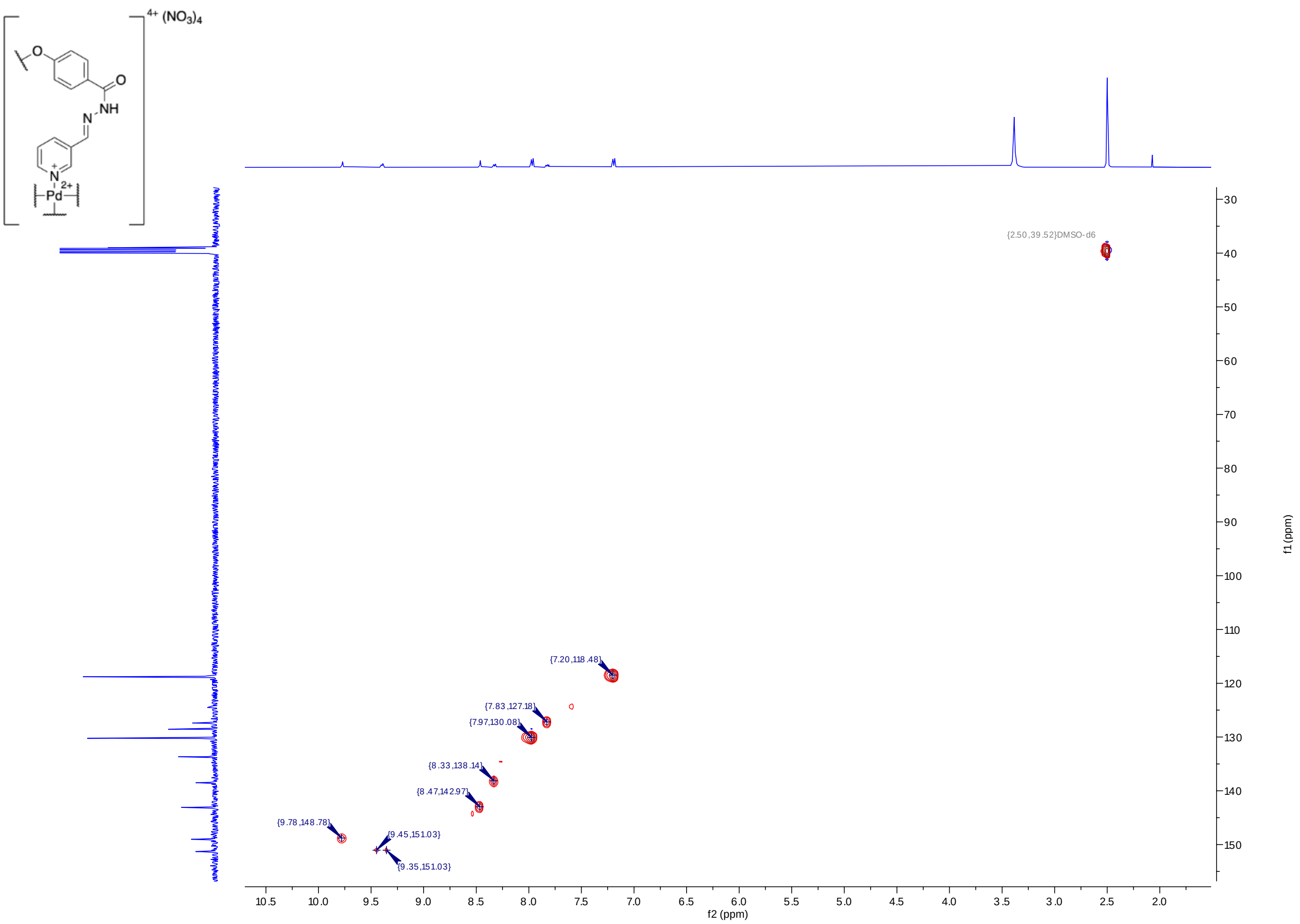

Figure S9. HSQC NMR (500 MHz, DMSO-d_6) of compound **C1·NO$_3$**.

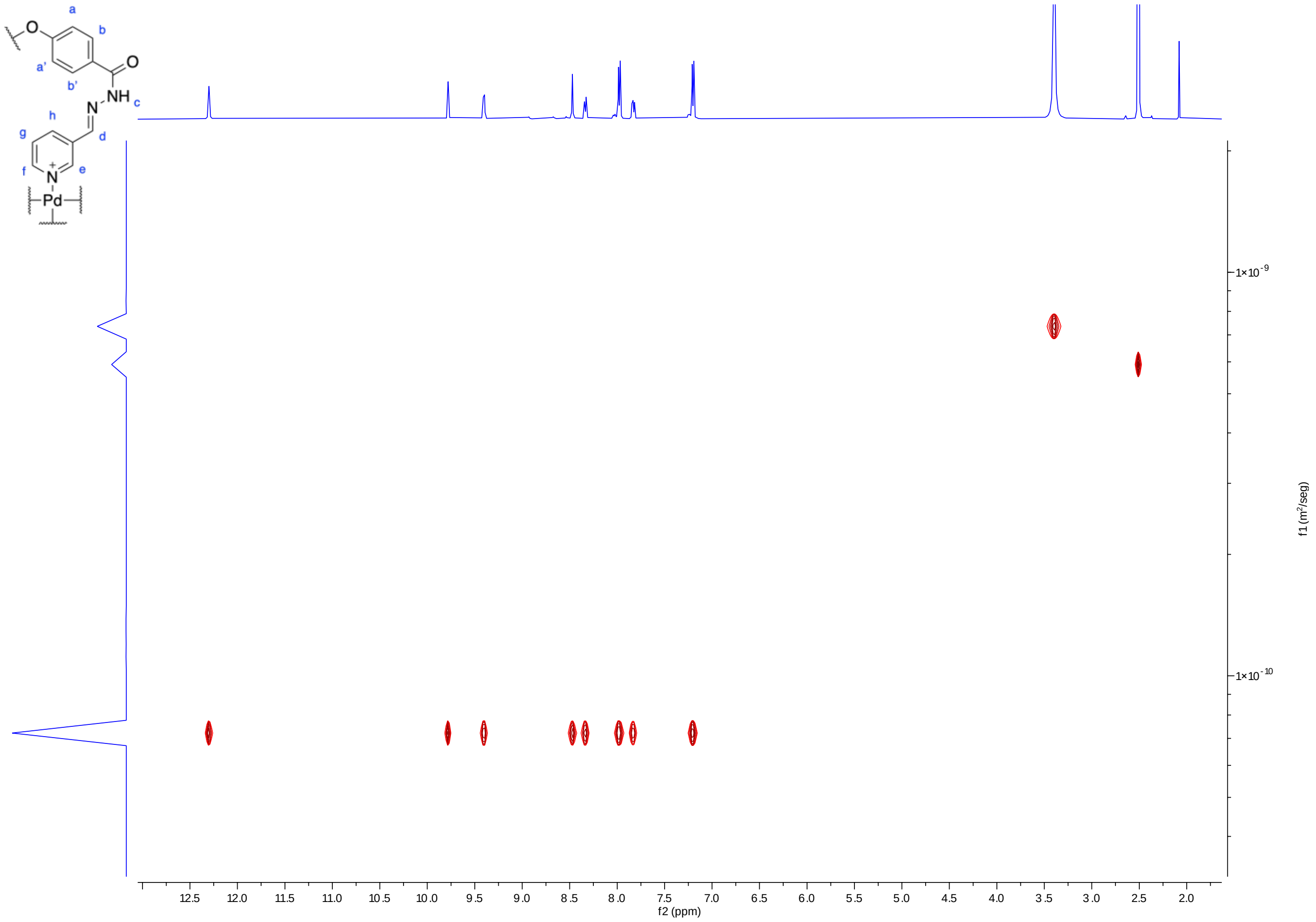

Figure S10. DOSY NMR (500MHz, DMSO-d_6) of compound **C1·NO_3**. D = $4.90\cdot10^{-11}$ $m^2\cdot s^{-1}$, R_h = 22.4 Å.

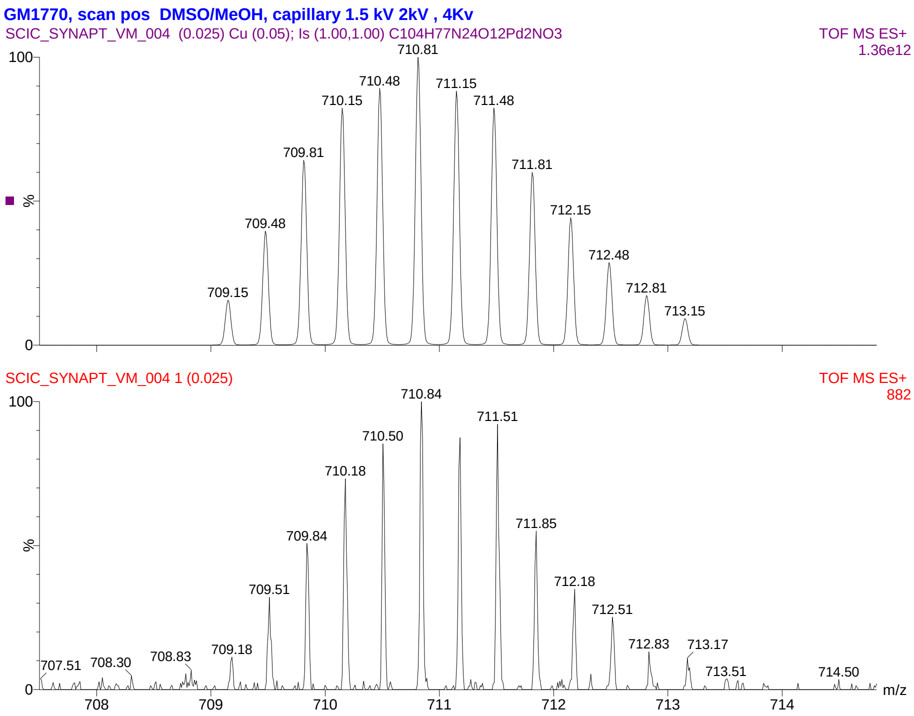

Figure S11. HRMS (SYNAPT XS high-definition mass spectrometer, Waters). Simulated (top) and measured (bottom) peaks of cage [**C1** + NO_3]$^{3+}$.

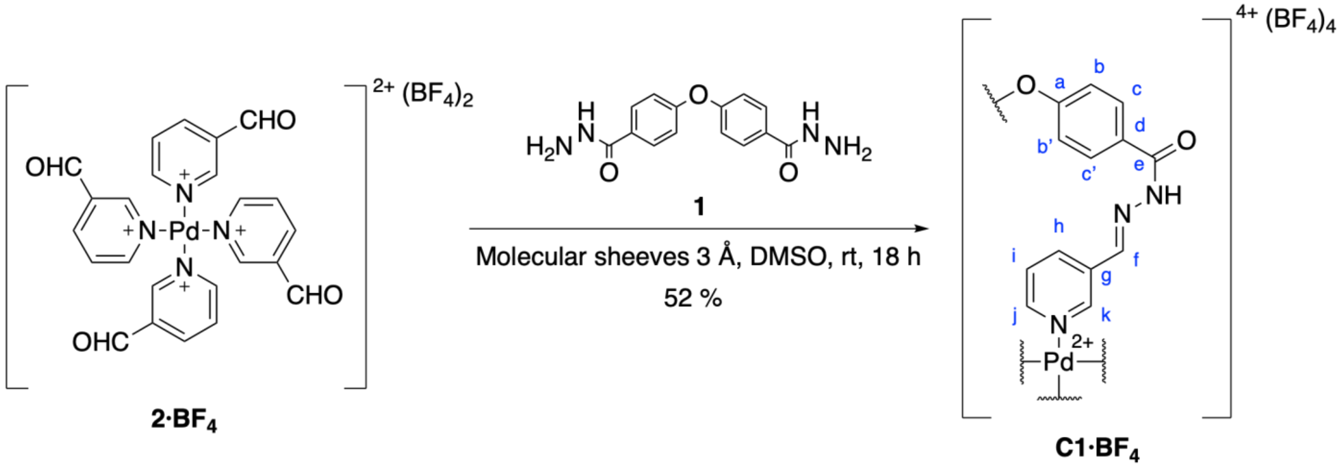

Synthesis of the Pd (II) Molecular Cage with BF_4^- counterion (C1·BF_4). 2·BF_4 (100 mg, 141.2 µmol) and **1** (80.9 mg, 282.5 µmol) were completely dissolved in of anhydrous DMSO (8.0 mL). The resulting solution was stirred at room temperature for 18 h, and then slowly poured into DCM (100 mL) until the formation of abundant white precipitate and left to sediment. The supernatant solution was removed, and the precipitate was dried out to obtain **C1·BF_4** pure as a white-grey solid (52 mg, 52 %).

^{1}H NMR (500 MHz, DMSO-d$_6$) δ 7.19 (1H, *d*, *J* = 8.7 Hz, H-b and H-b'), 7,78–7.87 (1H, *m*, H-i), 7.97 (1H, *d*, *J* = 8.7 Hz, H-c and H-c'), 8.31 (1H, *s*, H-d), 8.46 (1H, *s*, H-k), 9.38 (1H, *d*, *J* = 5.6, H-h), 9.78 (1H, s, H-f), 12.27 (1H, *s*, N*H*).

^{13}C NMR (125 MHz, DMSO-d$_6$) δ 118.8 (C-b and C-b'), 127.4 (C-g), 128.5 (C-a), 130.2 (C-c and C-c'), 133.7 (C-i), 138.6 (C-j), 143.1 (C-k), 148.9 (C-f), 151.3 (C-h), 159.2 (C-d), 162.7 (C-e).

^{19}F NMR (470 MHz, DMSO-d$_6$) δ -148.3 (BF_4^-).

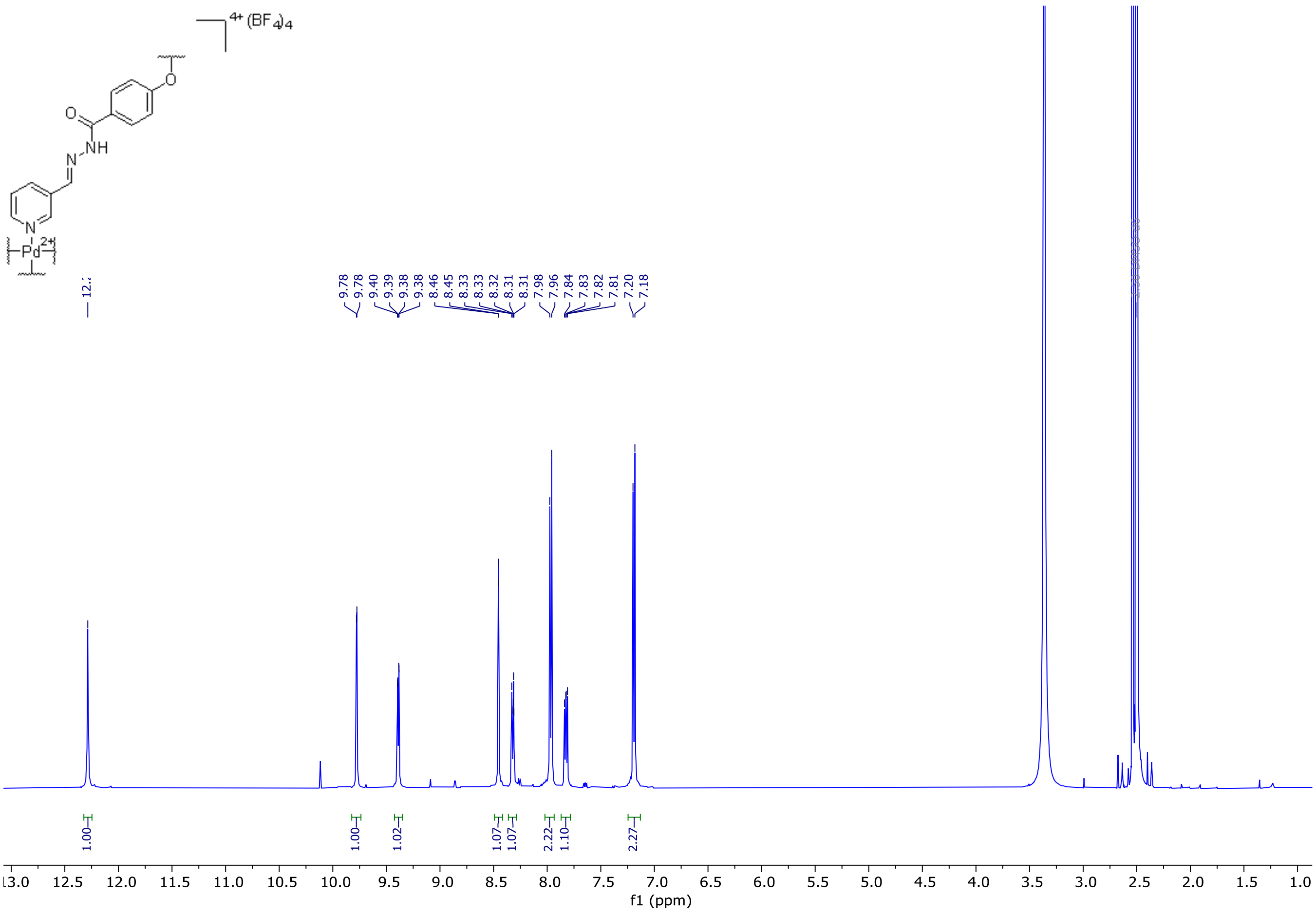

Figure S12. ^{1}H NMR of **C1·BF$_4$** (500 MHz, DMSO-d_6).

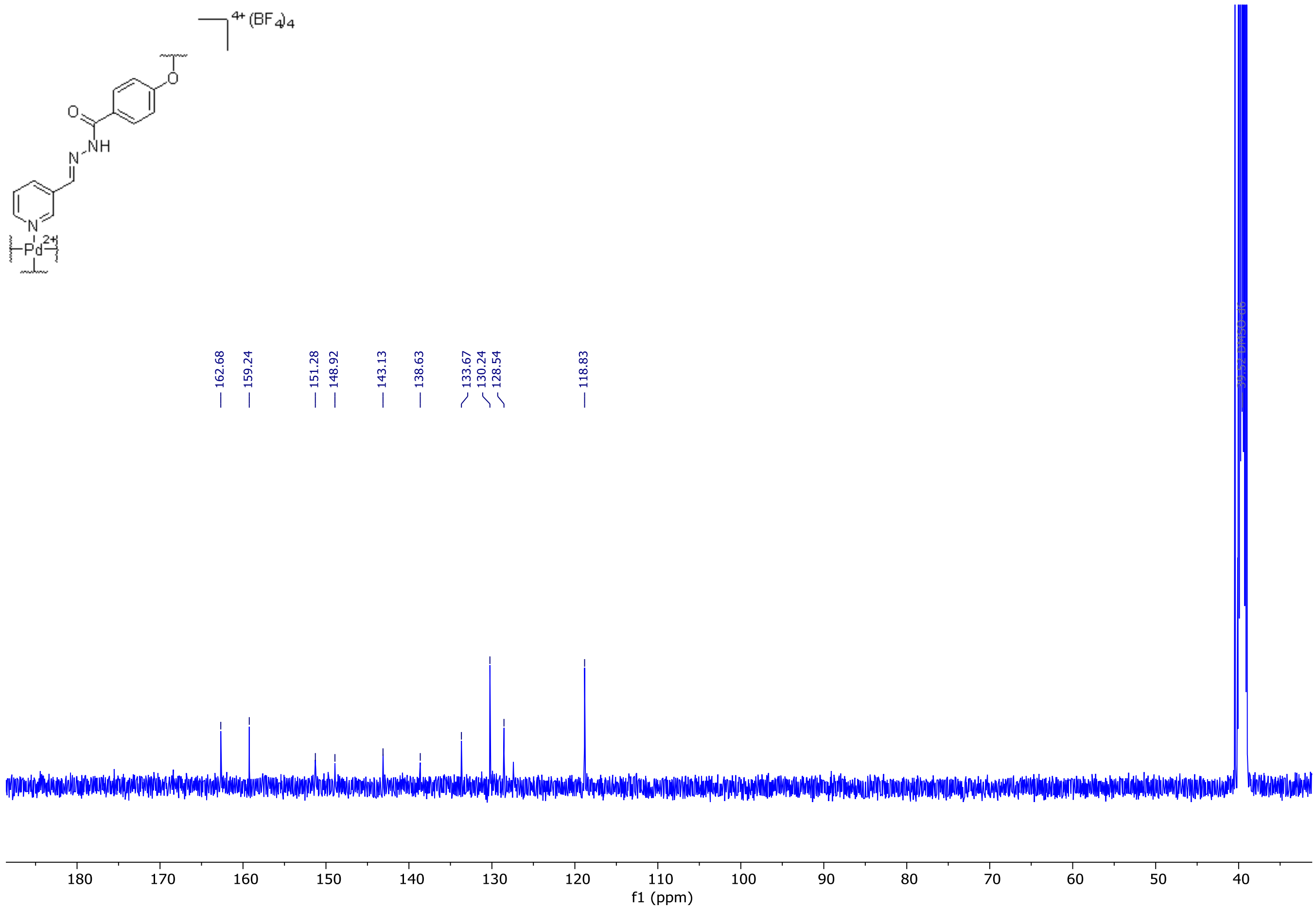

Figure S13. ^{13}C NMR of **C1·BF$_4$** (125 MHz, DMSO-d_6).

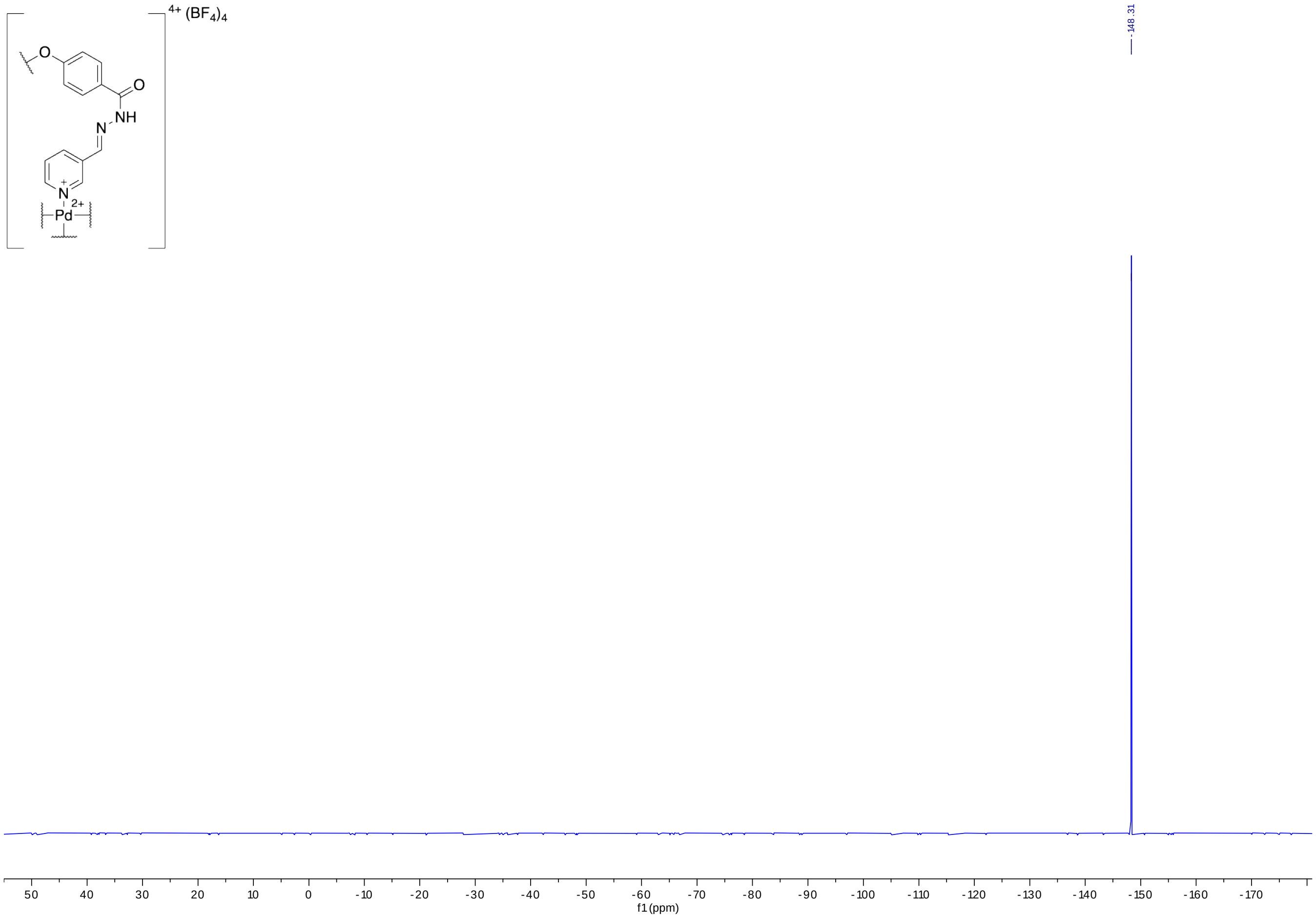

Figure S14. ^{19}F NMR of **C1·BF4** (470 MHz, DMSO-d_6).

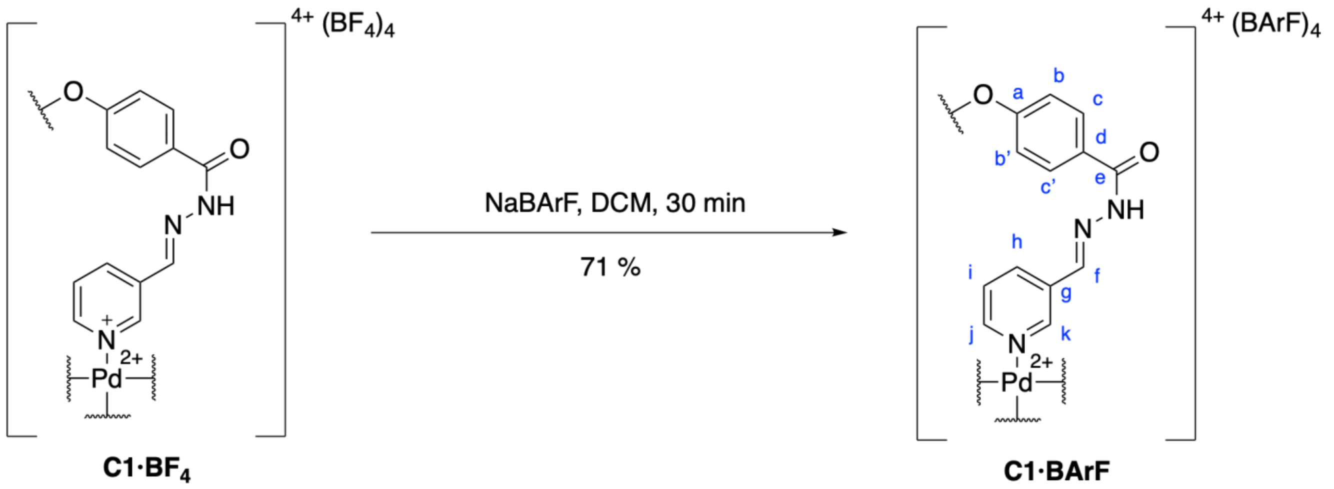

Synthesis of the Pd (II) Molecular Cage with BArF$^-$ counterion (C1·BArF). C1·BF4 (150.0 mg, 66.9 µmol) and NaBArF (281.5 mg, 317.6 mg) were dissolved in DCM (33.5 mL) and sonicated for 30 minutes. The suspension was vacuum filtered, and the filtered solution concentrated under vacuum to obtain a yellowish oil. The residue was redissolved in MeCN (3,4 mL) and water was added (66 mL) to obtain

abundant white precipitate which was vacuum filtered to obtain **C1·BF4** (242.7 mg, 71%) as a pure white powder.

^{1}H NMR (600 MHz, DMSO-d$_6$) δ 7.18 (2H, *d*, *J* = 8.7 Hz, H-b and H-b'), 7.54–7.68 (8H, m, H-BArF), 7.72 (4H, *bs*, H-BArF), 7.82 (1H, *m*, H-i), 7.97 (2H, *d*, *J* = 8.7 Hz, H-c and H-c'), 8.31 (1H, *d*, *J* = 8.4 Hz, H-j), 8.46 (1H, *s*, H-k), 9.38 (1H, *d*, *J* = 5.4 Hz, H-h), 9.79 (1H, *s*, H-f), 12.27 (1H, *s*, N*H*).

^{13}C NMR (150 MHz, DMSO-d$_6$) δ 117.7 (C-BArF), 118.8 (C-b and C-b'), 121.3 (C-BArF), 123.1 (C-BArF), 124.9 (C-g), 128.6 (C-a), 130.2 (C-c and C-c'), 133.7 (C-i), 134.0 (C-BArF), 138.7 (C-j), 143.1 (C-k), 148.8 (C-f), 151.3 (C-h), 159.2 (C-d), 160.4 (C-BArF), 160.8 (C-BArF), 161.1 (C-BArF), 161.4 (C-BArF), 162.6 (C-e).

^{19}F NMR (470 MHz, DMSO-d$_6$) δ -61.6 (BArF$^-$).

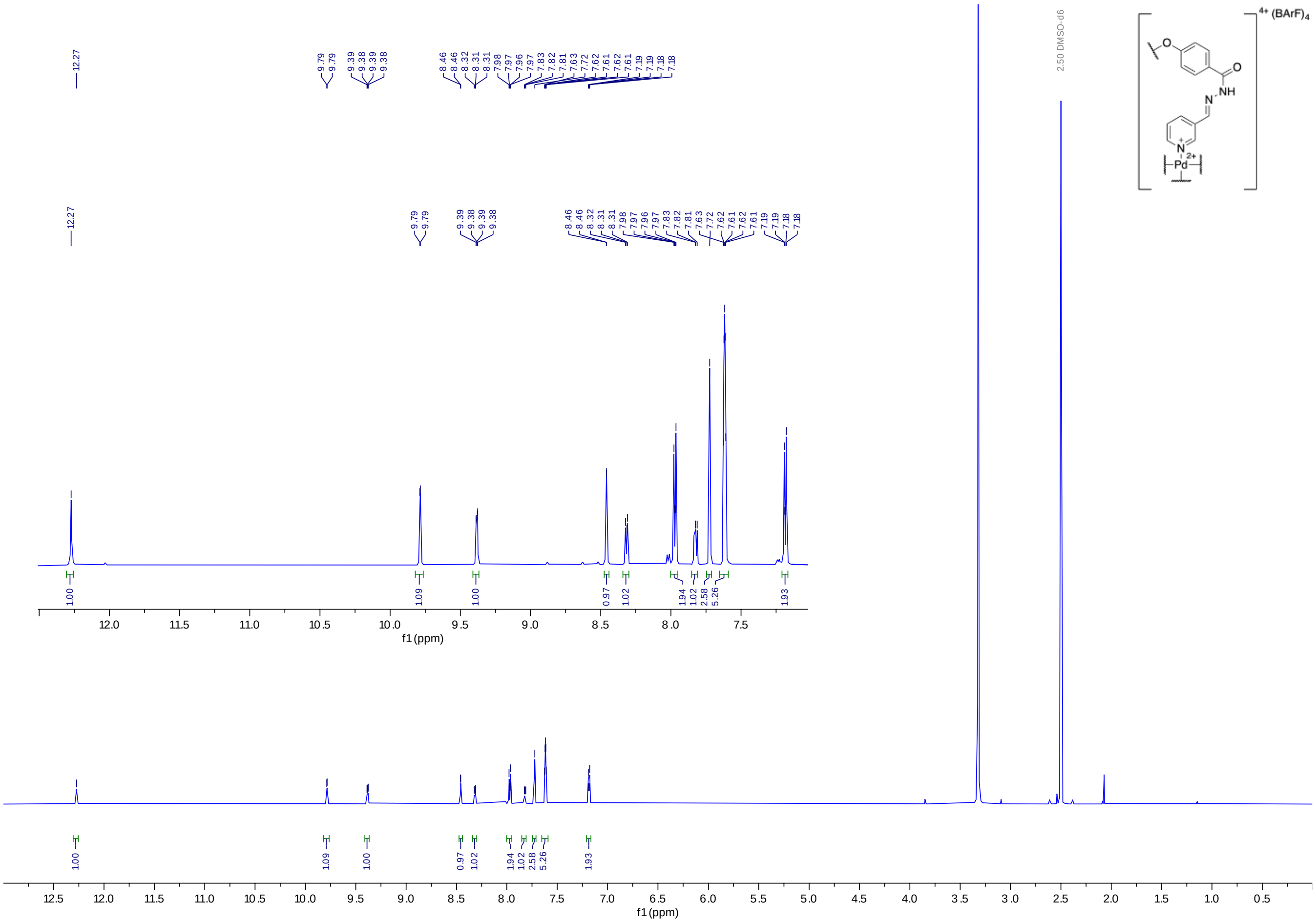

Figure S15. ^{1}H NMR of **C1·BArF** (600 MHz, DMSO-d_6).

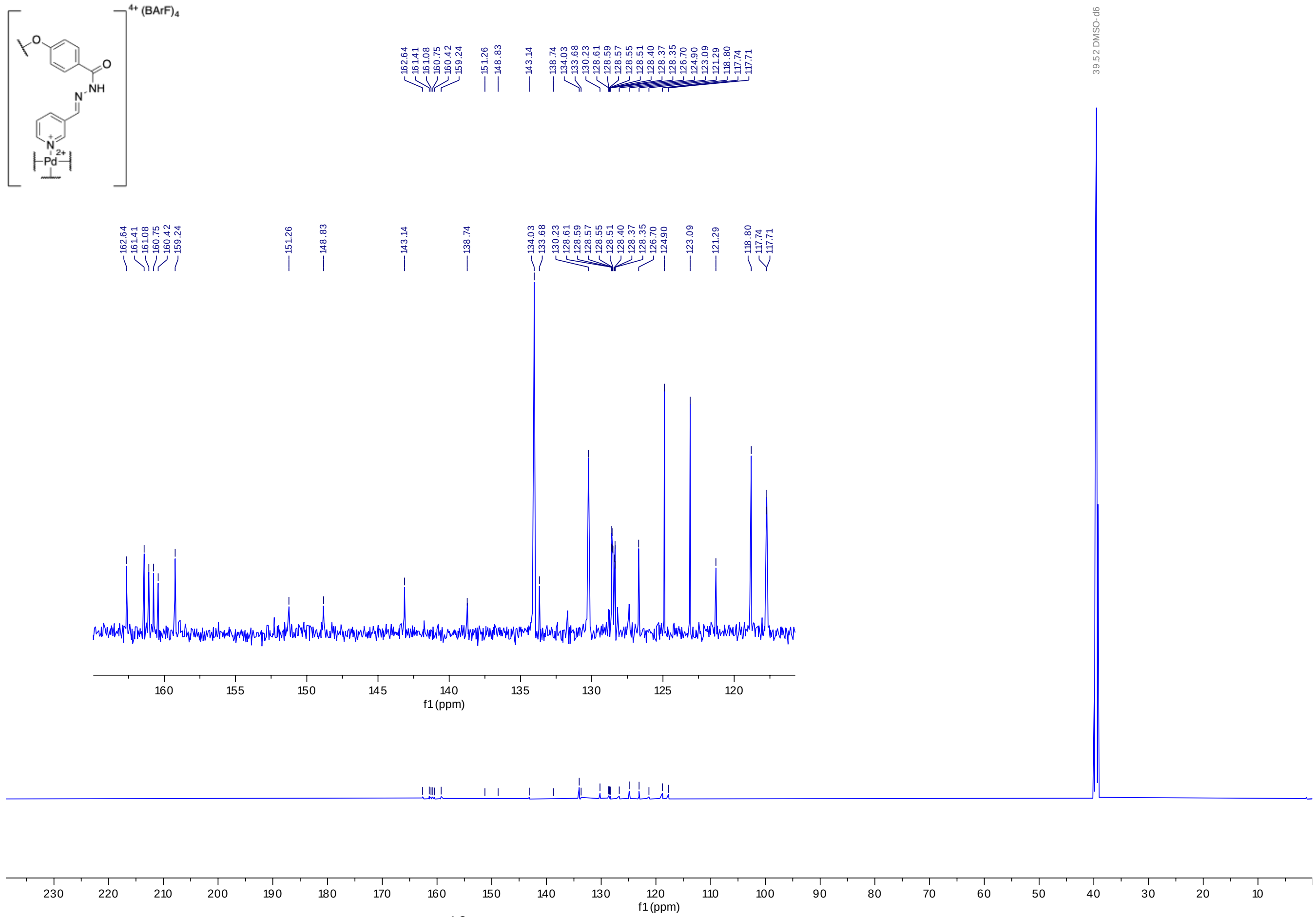

Figure S16. ^{13}C NMR of **C1·BArF** (150 MHz, DMSO-d_6).

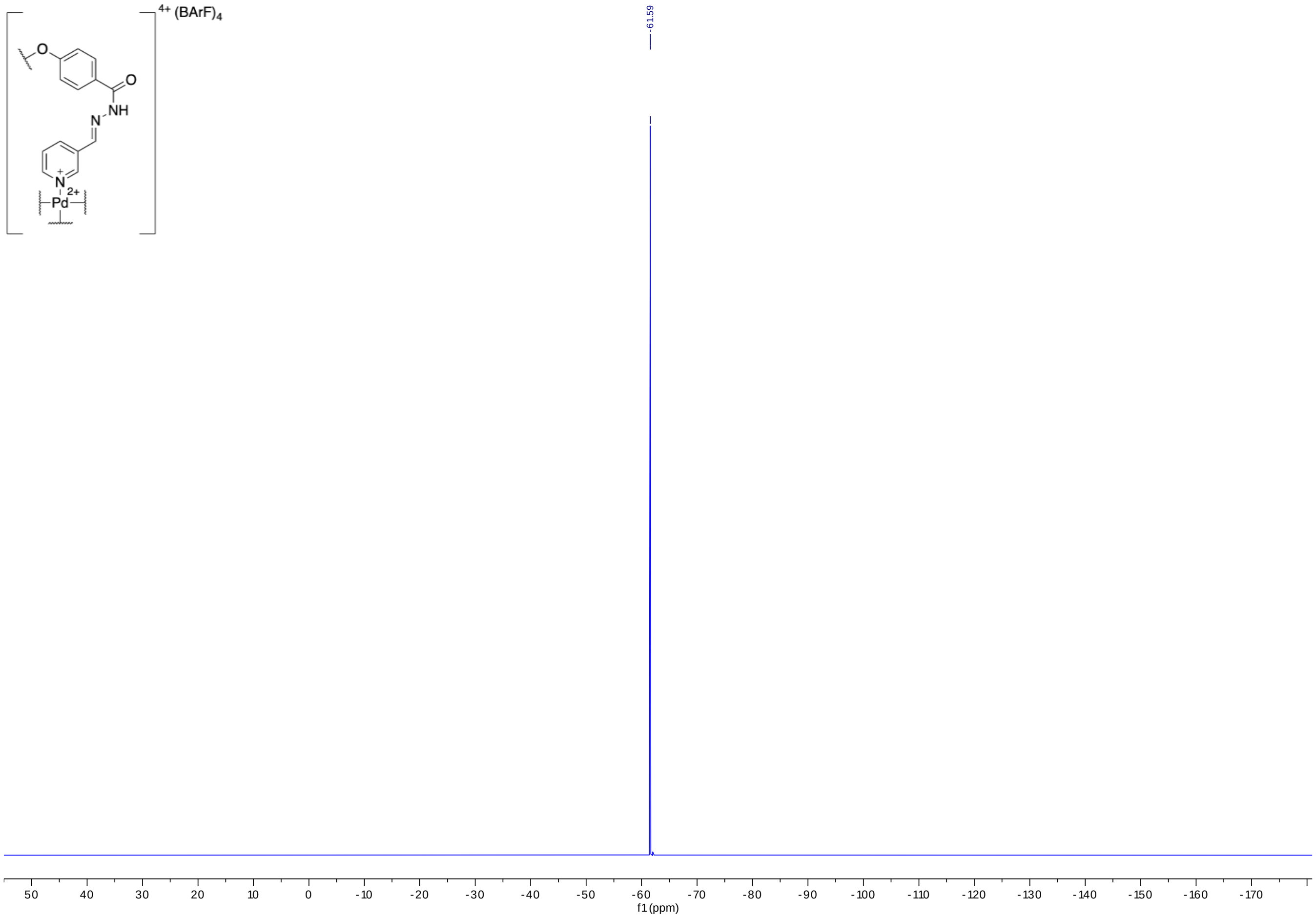

Figure S17. ^{19}F NMR of **C1·BArF** (470 MHz, DMSO-d_6).

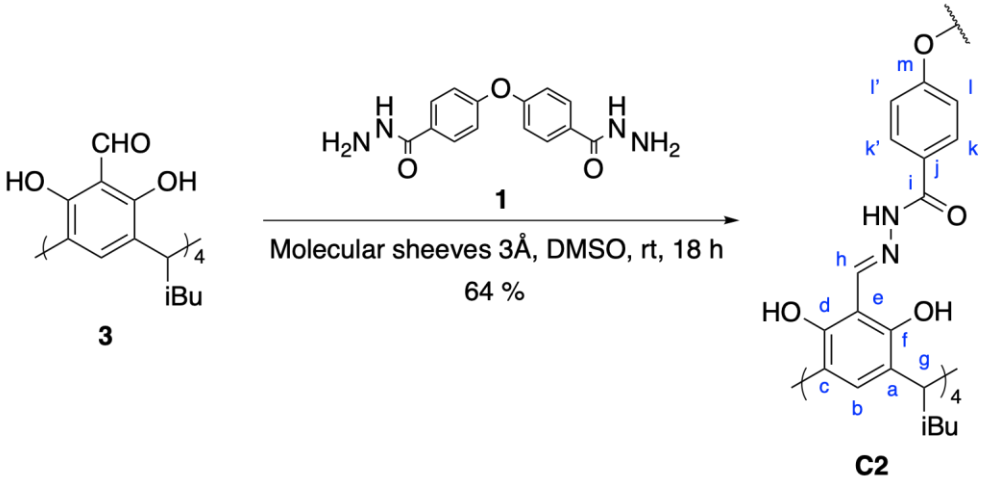

Synthesis of the pure organic molecular cage (C2): Cavitand **3** (50.0 mg, 60.66 µmol) and linker **1** (34.7 mg, 121.3 µmol) were dissolved in anhydrous DMSO (10.0 mL) under N_2 atmosphere with molecular sieves of 3Å to obtain an orange homogeneous solution. The resulting reaction mixture was stirred at room temperature over 18 h and then the solution was poured into AcOEt (40 mL) to obtain **C2** as a yellowish solid that was isolated by centrifugation (10 minutes at 10,000 rpm) (67.1 mg, 82%).

^{1}H NMR (500MHz, DMSO-d$_6$) δ 0.94 (6H, *d*, *J* = 6.5 Hz, CH_3-*i*Bu), 1.40 (1H, *s*, CH-*i*Bu), 1.95 (2H, *s*, CH_2-*i*Bu), 4.60 (1H, *s*, H-g), 7.21 (2H, *s*, H-l and H-l'), 7.41 (1H, *s*, H-b), 8.04 (2H, *s*, H-k and H-k'), 8.97 (1H, *s*, H-h), 12.15 (1H, *s*, N*H*).

^{13}C NMR (125 MHz, DMSO-d$_6$) δ 22.7 (*C*H_3-iBu), 25.1 (*C*H-*i*Bu), 26.0 (C-g), 79.2, 118.4 (C-l and C-l'), 119.3 (C-b), 128.8 (C-m), 129.2 (C-k and C-k'), 129.9 (C-h), 158.3 (C-j), 165.2 (C-i).

HRMS (ESI) *m/z* $[M + H]^{2+}$ calculated for $C_{152}H_{154}N_{16}O_{28}$ 1,326.06, found 1,326.10.

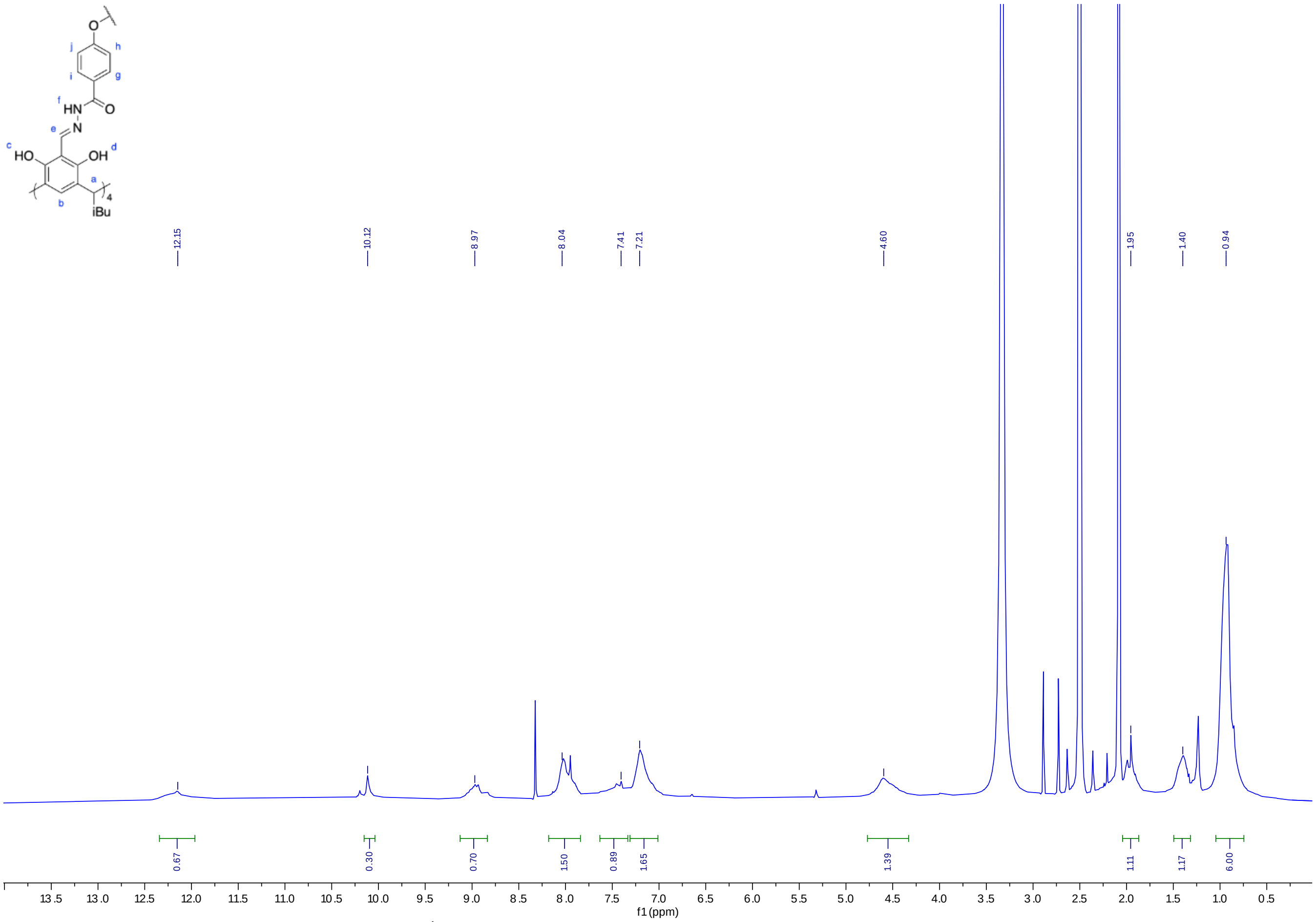

Figure S18. ^{1}H NMR (500MHz, DMSO-d_6) of compound **C2**.

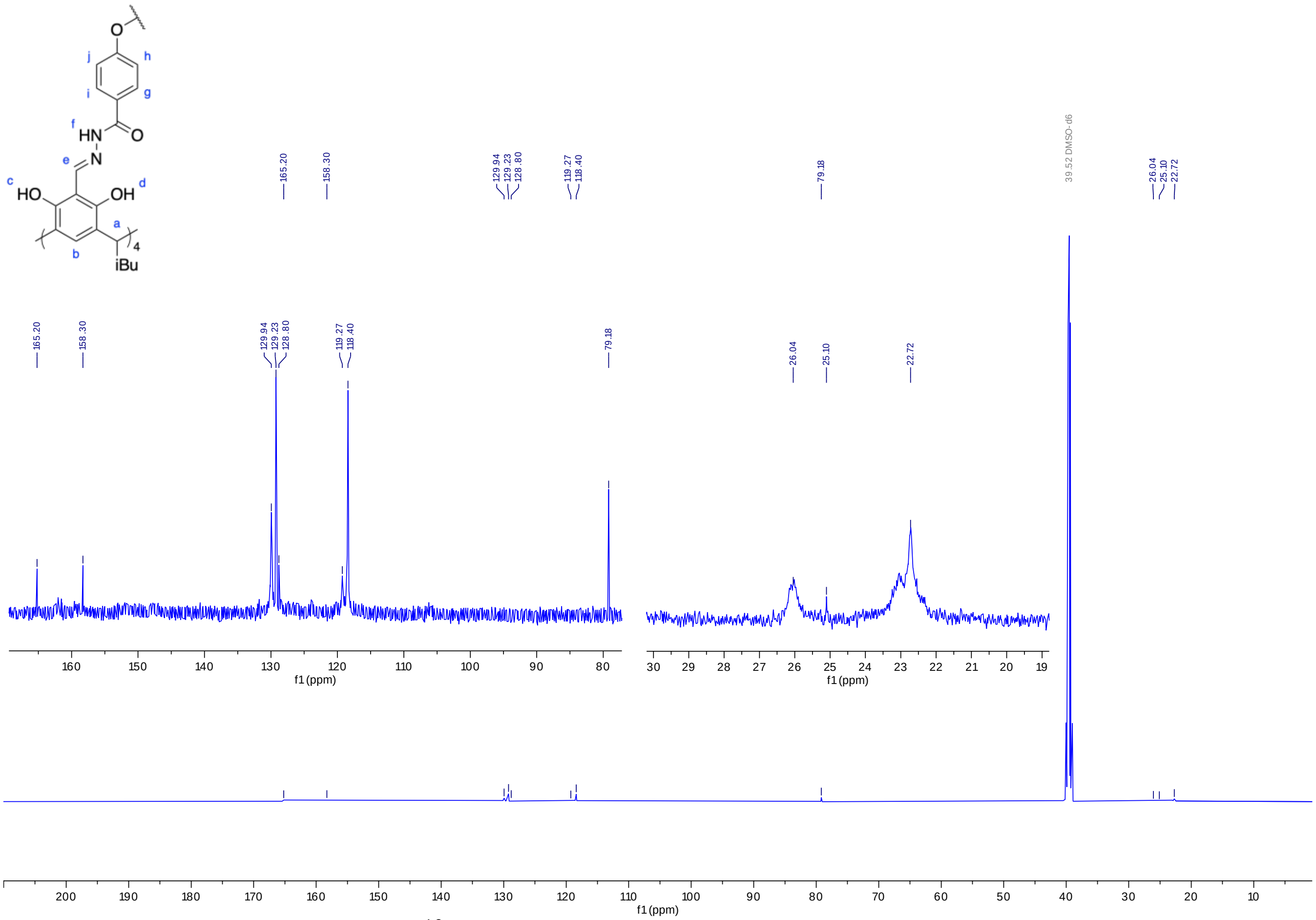

Figure S19. ^{13}C NMR (126MHz, DMSO-d_6) of compound **C2**.

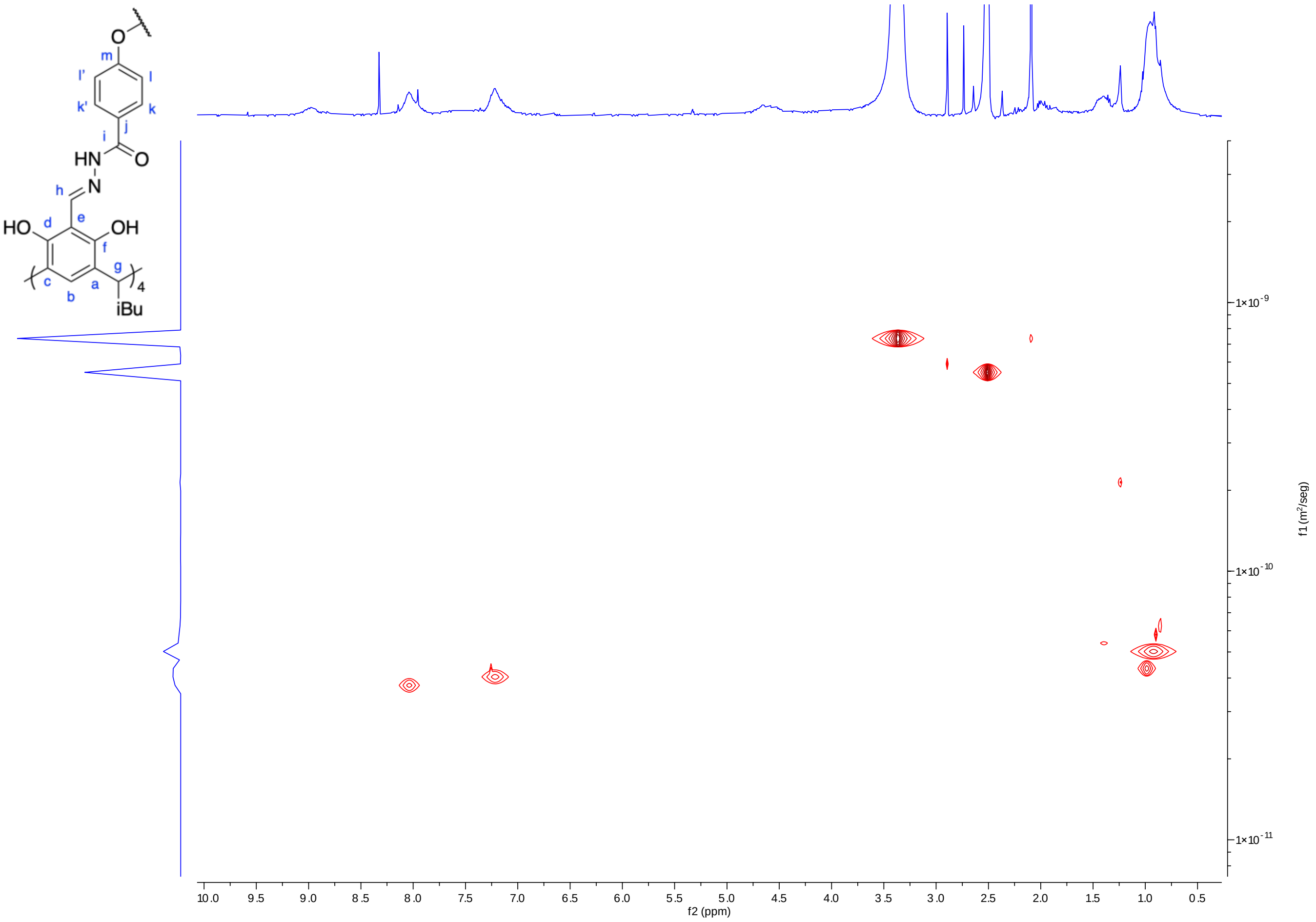

Figure S20. DOSY NMR (500MHz, DMSO-d_6) of compound **C2**. D = $4.44 \cdot 10^{-11}$ $m^2 \cdot s^{-1}$, $R_{hydrodynamic}$ = 24.7 Å.

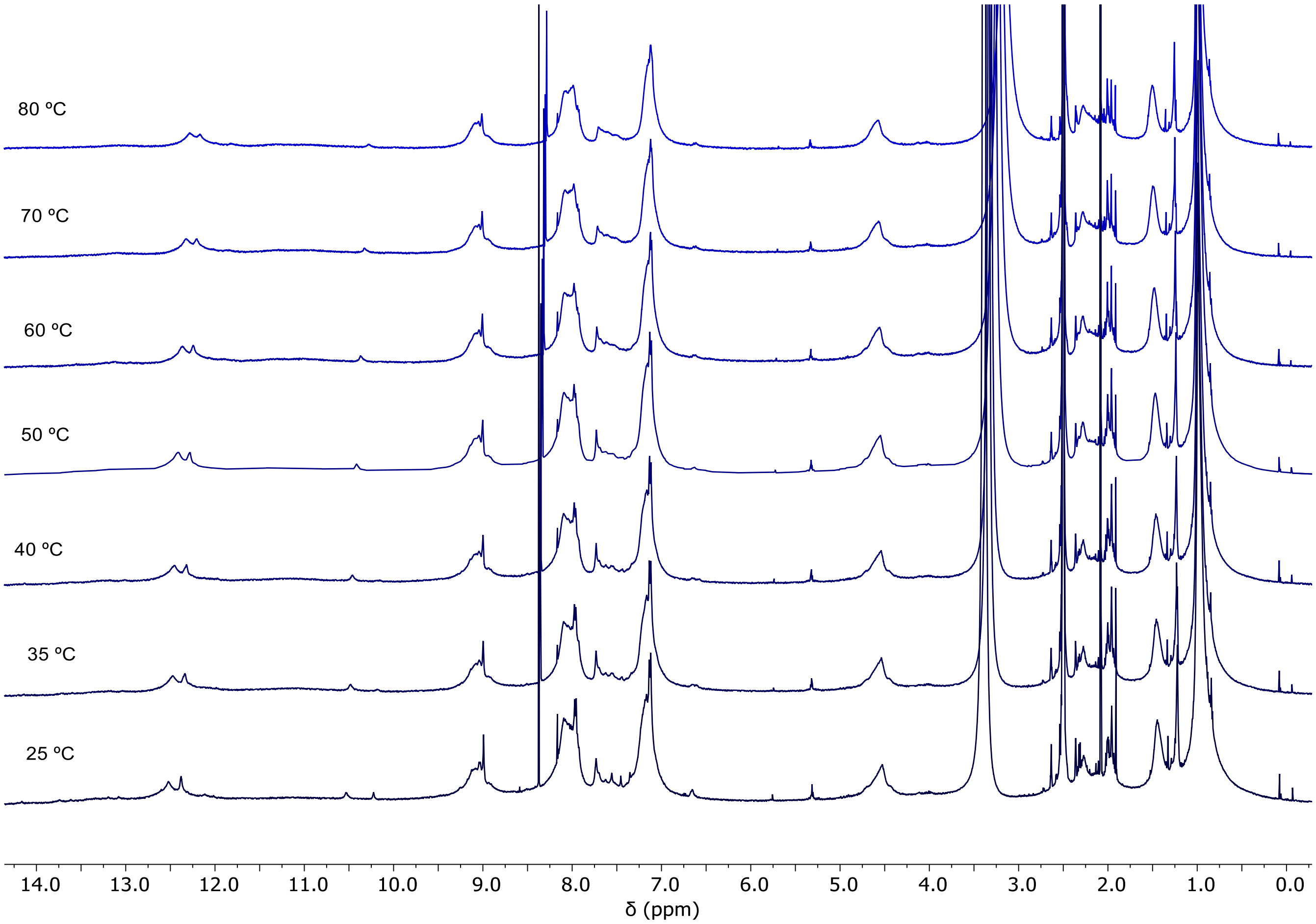

Figure S21. VT NMR (500MHz, DMSO-d_6) of compound **C2**.

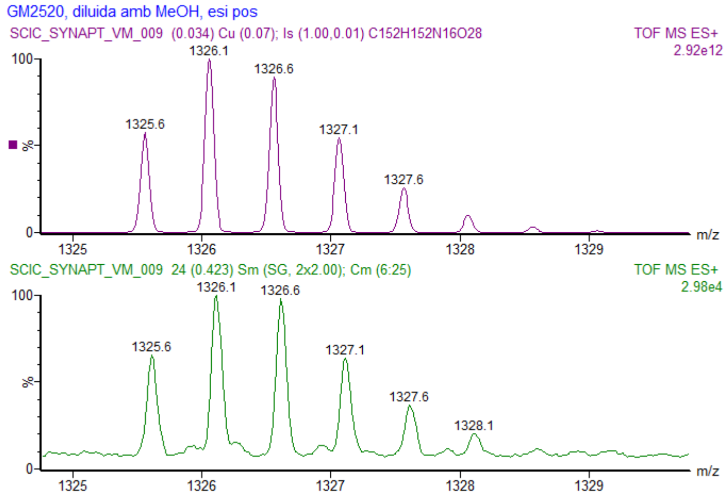

Figure S22. HR-MS (SYNAPT XS high-definition mass spectrometer, Waters). Simulated (top) and measured (bottom) peaks of cage [**C2**+2H]$^{2+}$.

3. X-Ray crystallographic data for C1·NO_3

Single crystals of **C1·NO_3** were grown by vapor diffusion of CH_3OH to a solution of the cage in DMSO over a week. A suitable crystal was selected and placed on a Bruker D8 Venture Diffractometer. The crystal was kept at 120.0 K during data collection. Using Olex2[S9], the structure was solved with the SHELXS[S10] structure solution program using Direct Methods and refined with the SHELXL[S11] refinement package using Least Squares minimisation. The structure contains disordered solvent molecules that could not be modeled, the corresponding contribution of disordered solvent molecules was handled by the solvent mask command in Olex2. The structure has 4 nitrate counter anions, only two of them could be located inside the cavity and the other two nitrate anions are presumably placed outside the cavity with a high disorder making it not possible to find them. The two nitrate anions in the cavity could be located, one of them without disorder, and the second one was modeled with a disorder over 3 positions, being only possible to fully find it in one of these 3 disordered positions. A twin law was used to model the enantiomeric disorder, i.e. the cage has a small helicity at the 50-50 ratio. CCDC deposition number 2295536. **Crystal Data** for **C1·NO_3**: monoclinic, space group $P2_1$ (no. 4), a = 19.6620(13) Å, b = 23.7645(17) Å, c = 19.8456(14) Å, β = 113.508(2)°, V = 8503.4(10) Å^3, Z = 4, T = 120.0 K, μ(MoKα) = 0.315 mm^{-1}, $Dcalc$ = 0.973 g/cm^3, 113633 reflections measured (4.132° ≤ 2Θ ≤ 50.782°), 31019 unique (R_{int} = 0.0595, R_{sigma} = 0.0565) which were used in all calculations. The final R_1 was 0.0449 (I > 2σ(I)) and wR_2 was 0.1162 (all data).

Table S1. Crystal data and structure refinement for **C1·NO_3**.

CCDC number	2295536
Temperature/K	120.0
Crystal system	monoclinic
Space group	$P2_1$
a/Å	19.6620(13)
b/Å	23.7645(17)
c/Å	19.8456(14)
α/°	90
β/°	113.508(2)
γ/°	90
Volume/Å^3	8503.4(10)
Z	4
ρ_{calc}g/cm^3	0.973
μ/mm^{-1}	0.315
F(000)	2566.0
Crystal size/mm^3	0.201 × 0.164 × 0.056
Radiation	MoKα (λ = 0.71073)
2Θ range for data collection/°	4.132 to 50.782
Index ranges	-23 ≤ h ≤ 23, -28 ≤ k ≤ 28, -23 ≤ l ≤ 23
Reflections collected	113633
Independent reflections	31019 [R_{int} = 0.0595, R_{sigma} = 0.0565]
Data/restraints/parameters	31019/346/1522
Goodness-of-fit on F^2	1.037
Final R indexes [I>=2σ (I)]	R_1 = 0.0449, wR_2 = 0.1073
Final R indexes [all data]	R_1 = 0.0628, wR_2 = 0.1162
Largest diff. peak/hole / e Å^{-3}	0.65/-0.33
Flack parameter	0.487(17)

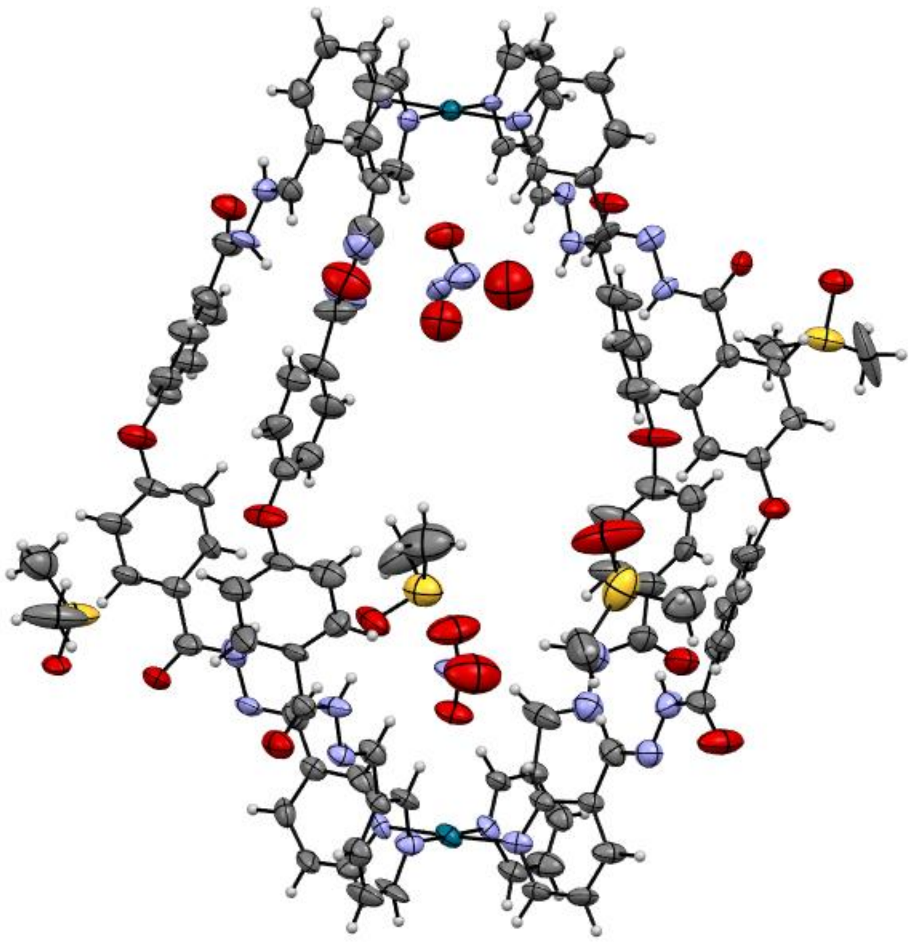

Figure S23. X-Ray crystal structure of cage **C1·NO3**.

4. NMR spectrometric host-guest experiments and cage disassembly

NMR titration experiments were carried out on an AVA400 NMR spectrometer equipped with a BBFO$^+$ room temperature probe featuring two channels: ^{1}H and X/^{19}F (optimised).

The titration experiments were performed using a 10 mM solution of DOXO in CD_3OD and a 500 µM solution of **C1·BArF** in a DCM-d_2/CD_3OD (9:1) mixture in order to achieve the complete dissolution of the molecular cage and DOXO. The spectra stacked in **Figure S24** show chemical shifts after the addition of increasing equivalents of DOXO to a **C1·BArF** solution. In previous experiments, it was observed that the presence of water promotes precipitation of the cage, which makes **C1** not suitable for biological applications. Increasing equivalents of DOXO eventually leads to the disassembly of the molecular cage.

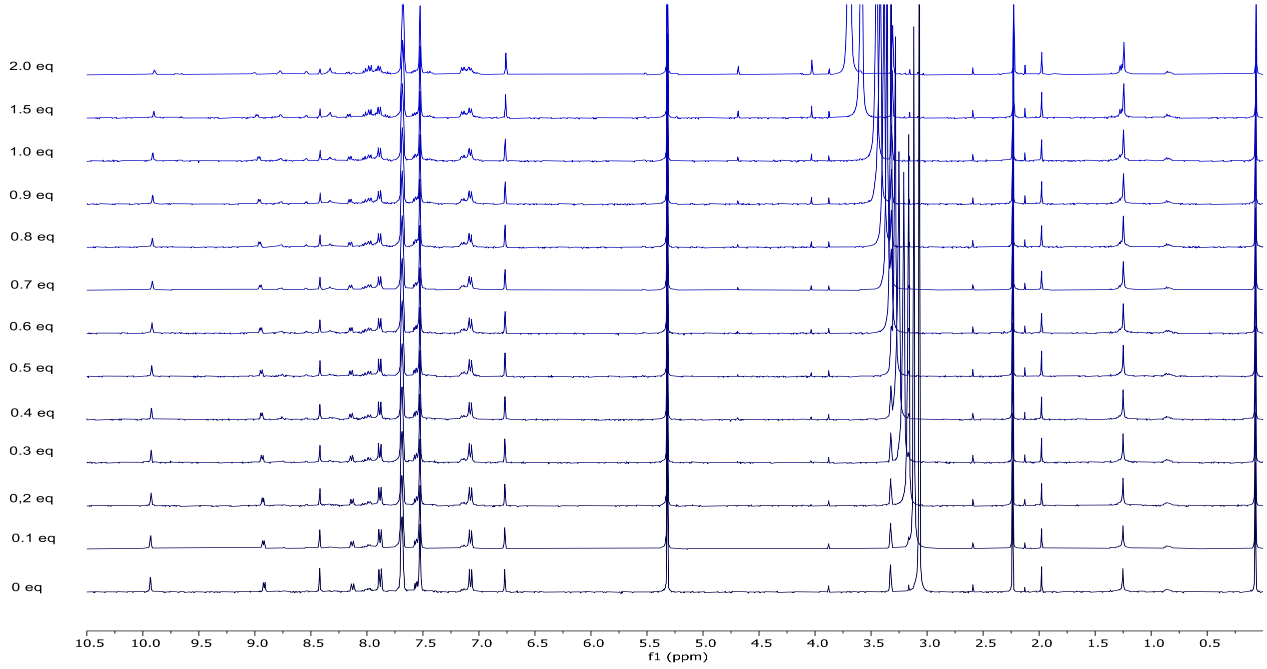

Figure S24. Titration experiments of **C1·BArF** (500 µM) *versus* DOXO (10 mM in CD_3OD) in a DCM-d_2/CD_3OD (9:1) mixture (0−2.0 equivalents).

The low solubility of **C2** in DMSO-d_6/D_2O mixtures for NMR experiments led to carrying out the titration experiments with DOXO in a DMSO-d_6/CD_3OD (8:2) mixture (**Figure S25**). The chemical shifts of **C2** suggest the encapsulation of DOXO. However, the weak interaction observed is likely associated with the high amount of DMSO-d_6 present in the sample.

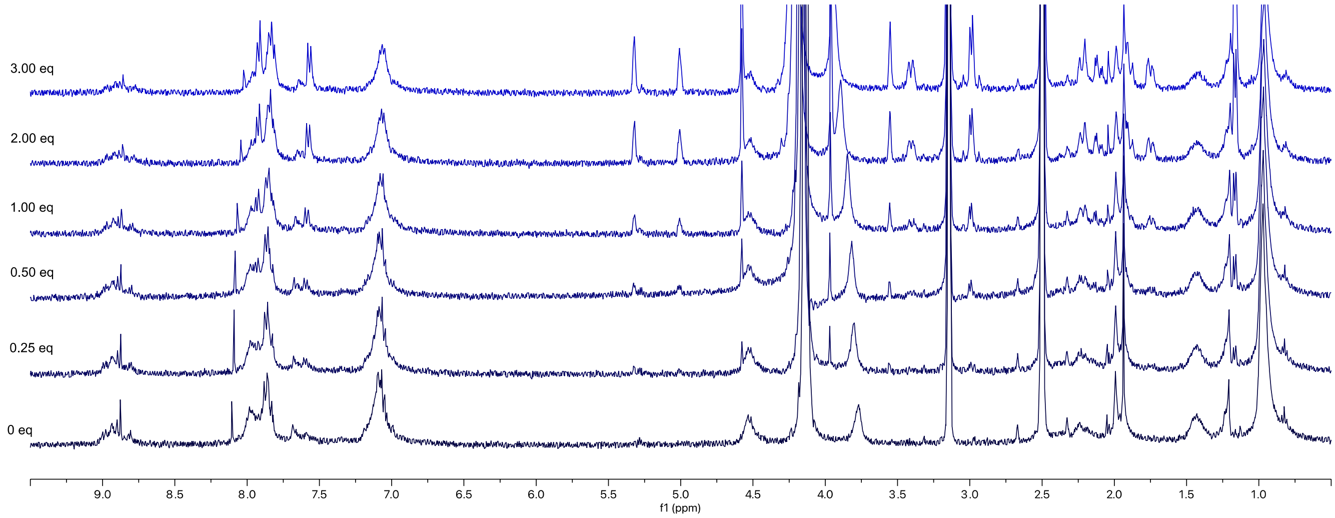

Figure S25. Titration experiments of **C2** (500 µM) *versus* DOXO (10 mM in CD_3OD) in a DMSO-d_6/CD_3OD (8:2) mixture.

Despite that the changes observed in the chemical shifts in the titrations, they show binding between the host at the guest (**Figure S26**).

Investigation with the metallo-organic cage was carried out using a CD_2Cl_2/CD_3OD (9:1) solution of **C1·BArF**, to which DOXO was added in CD_3OD (Figure S26a). These experiments showed noticeable chemical displacements of the protons H_f ($\Delta\delta$ = -0.04 ppm) and H_h and H_j ($\Delta\delta$ = 0.10 ppm) (FigureS26a). The upfield shift of the inward-facing H_f protons evidences the encapsulation of DOXO inside the cavity. While downfield shift of the outer protons H_h and H_j upon DOXO binding are also observed, these could be due to subtle conformational changes (e.g., a twisting of the hydrazone group leading to a change in a N···H_h–C H-bond) or electronic effect–communication through the ring system.[S12,S13] The lack of chemical shift change in H_k suggests that the binding mode is different to what is normally observed in Pd_2L_4 cages, wherein the inward facing *ortho*-pyridyl H atoms create a H-bond donor pocket that that can interact with H-bond acceptor groups.[S12] It should also be noted that increasing amounts of guest (>0.6 eq) produce a decrease of integration of cage signals suggestive of disassembly, possibly by complexation of Pd^{2+} by the NH_2 moiety of DOXO. This again highlights the challenges in using metallo-organic cages in bio-medical applications.

For cage **C2**, it was found that DMSO-d_6/CD_3OD (8:2) was an optimal mixture in terms of cage solubility to probe binding by NMR (Figure S26b). The addition of DOXO revealed upfield changes of the protons inside the cavity H_h ($\Delta\delta$ = -0.020 ppm), $H_{k/k'}$ ($\Delta\delta$ = -0.02 ppm), $H_{l/l'}$ ($\Delta\delta$ = -0.02 ppm) and H_g ($\Delta\delta$ = 0.04 ppm). The upfield shift in three of these signals is consistent with the formation of CH···π interaction between the anthracycline system of DOXO and the inward-facing protons of **C2**. Similar shielding effects have been observed for many different cage systems.[S14,S15,S16]

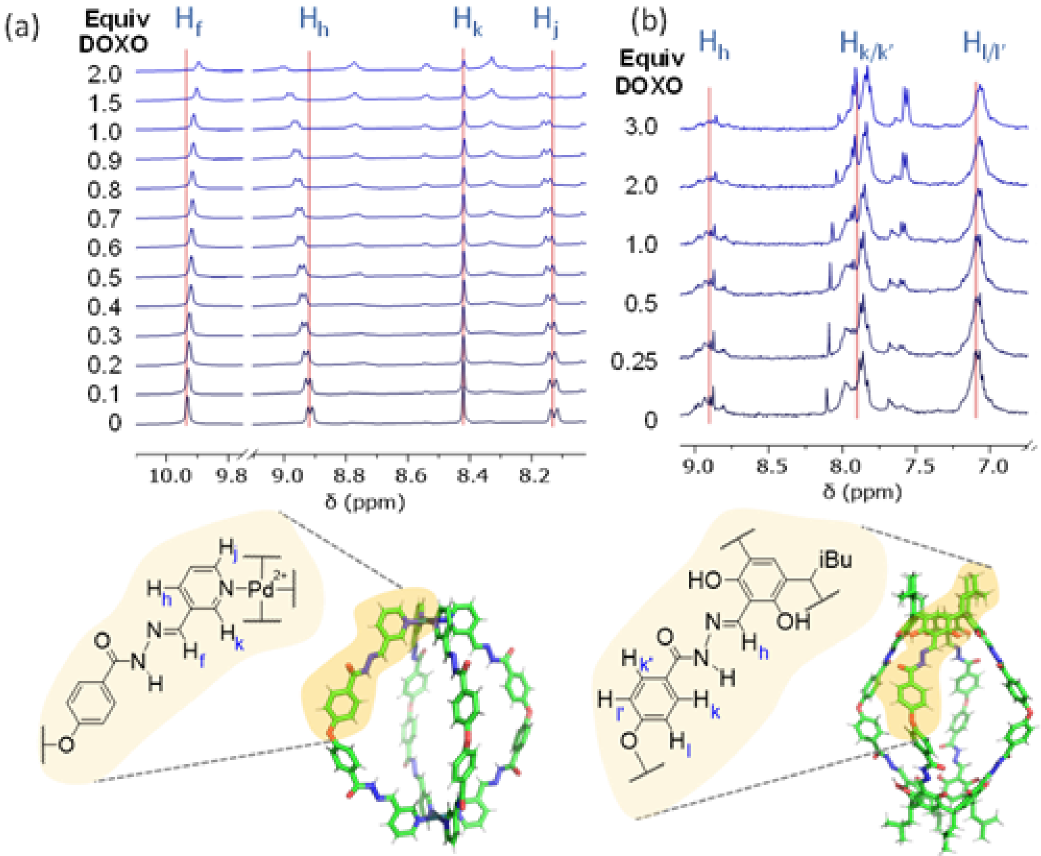

Figure S26. Partial 1H NMR spectra (400 MHz, 25 ºC) for the titration of cages **C1** and **C2** with DOXO. (a) Addition of DOXO (CD_3OD) to **C1**·BArF (0.5 mM, CD_2Cl_2/CD_3OD 9:1); (b) Addition of DOXO (CD_3OD) to **C2** (0.5 mM, DMSO-d_6/CD_3OD 8:2).

Ligand displacement of cage **C1**·**NO_3** was obtained by recording the ^{1}H NMR spectra of **C1**·**NO_3** in DMSO-d_6 (600 µL, 0.5 mM) after the addition of phosphate buffer (1 µL, 150 µM, pH 7.2) over a period of 4 days (**Figure S27**).

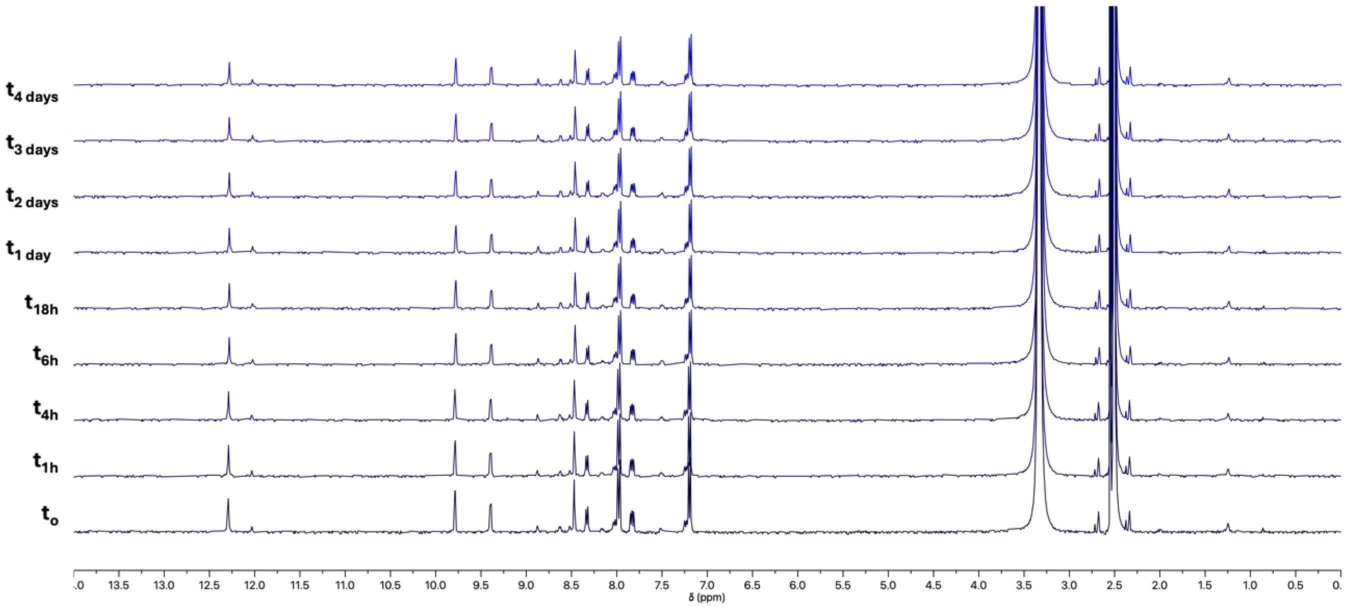

Figure S27. ^{1}H NMR disassembly experiment of cage **C1·NO3** in DMSO-d_6 (0.5 mM) with phosphate buffer (150 μM, pH 7.2) monitored over time (0−4 days), resulting in 0.2% H_2O in DMSO-d_6.

5. Spectrophotometric experiments

Experimental details

All UV-visible and spectrofluorimetric titration experiments were carried out on a Perkin Elmer EnSpire 2300 Multimode Plate Reader. The titration experiments and the Job Plot method were performed at room temperature using a 96-well plate. The titration experiments were carried out by adding increasing equivalents (0–2.3 or 2.8) of 4 mM (**C2**) or 10 mM (**C1**) molecular cage stock solutions in DMSO to a 50 µM solution of DOXO in phosphate buffer (without NaCl) at pH 7.1. The measurements were made with maximal volumes of 250 µL and a 50 µM concentration. All experiments were performed with phosphate buffer (without NaCl) at pH 7.1 or milli-Q water with a percentage of DMSO (0–10%). All emission spectra were recorded in a 700–250 nm wavelength range, while excitation spectra were recorded in a 510–700 nm wavelength range for spectrofluorimetric measurements with an excitation wavelength of 470 nm (excitation wavelength for DOXO). The binding stoichiometry between the molecular cage (**C1** or **C2**) with DOXO was determined by Job Plot's Method (or Method of Continuous Variation) by fluorimetry titration experiments. In a 96-well plate, serial molecular cage's (**C1** or **C2**) solutions were placed in phosphate buffer (without NaCl) at pH 7.1 with decreasing concentration, to which an increasing number of equivalents of DOXO were added to have a 0–1.0 molar concentration series of both species with a 0.05 molar fraction range. The titrations were performed in quadruplicate, and the standard error was obtained for each titration point.

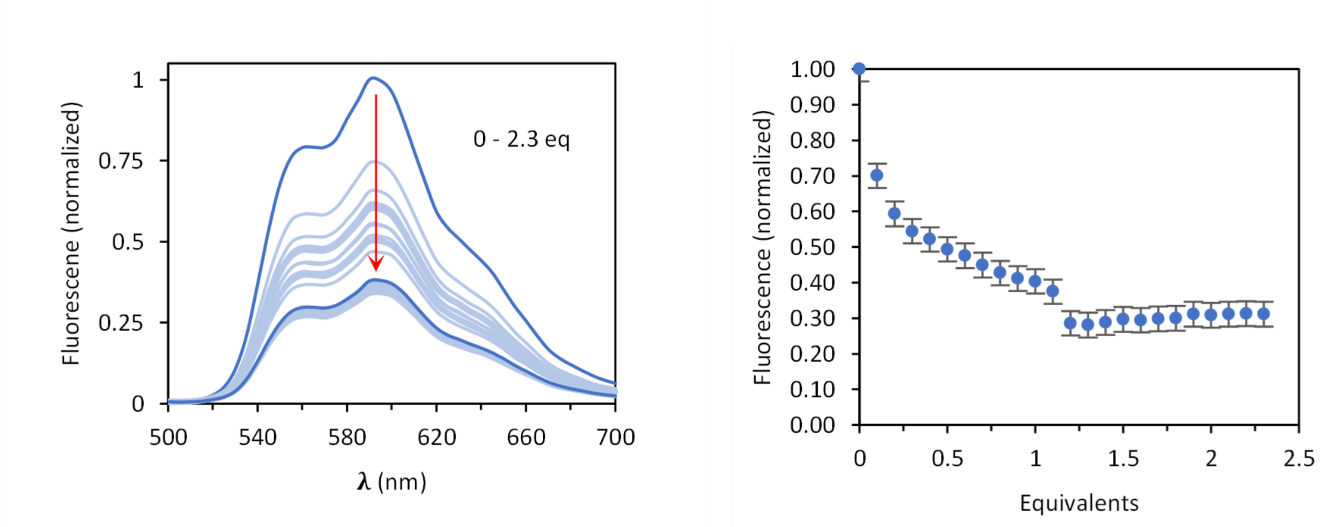

Figure S28. Fluorescence emission spectra (H_2O with 0−10% DMSO and milli-Q water, rt) of the titration of doxorubicin (50 µM) with cage **C1** (0 to 2.3 equivalents). Representative titration experiment (left) and variation of the fluorescence as the average with standard error of 4 independent titrations (right). Perkin Elmer EnSpire 2300 Multimode Plate Reader.

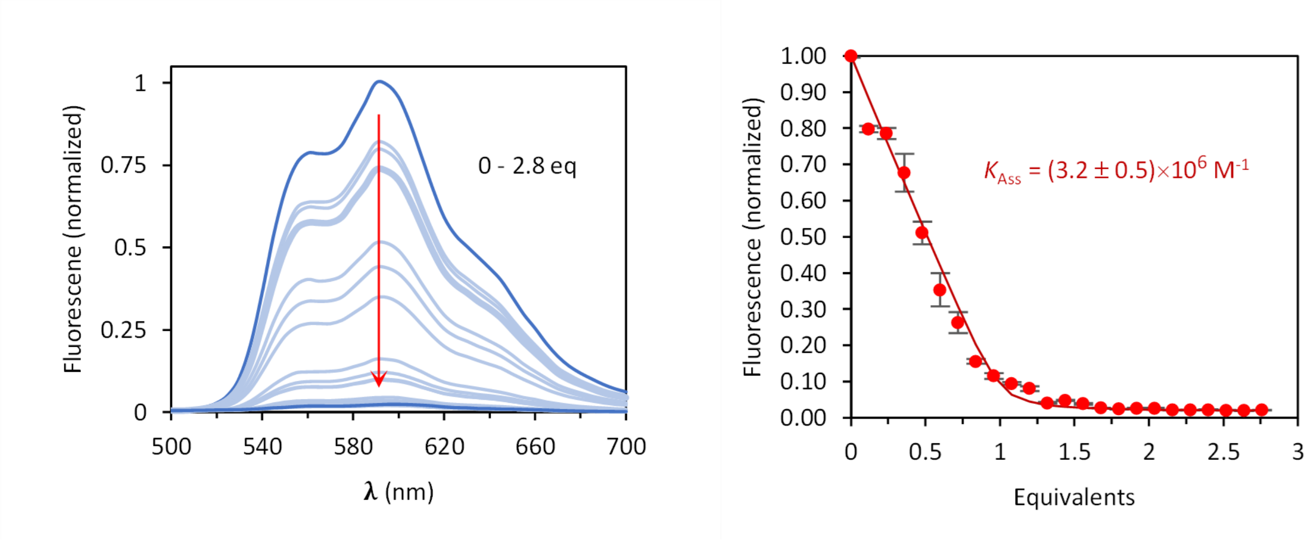

Figure S29. (a) Fluorescence emission spectra (H_2O with 0−10% DMSO and phosphate buffer 100 µM, pH 7.1, rt) of the titration of doxorubicin (50 µM) with cage **C2** (0 to 2.8 equivalents). Representative titration experiment (left) and variation of the fluorescence as the average of 4 independent titrations with the corresponding standard error for each titration pount (right). Perkin Elmer EnSpire 2300 Multimode Plate Reader.

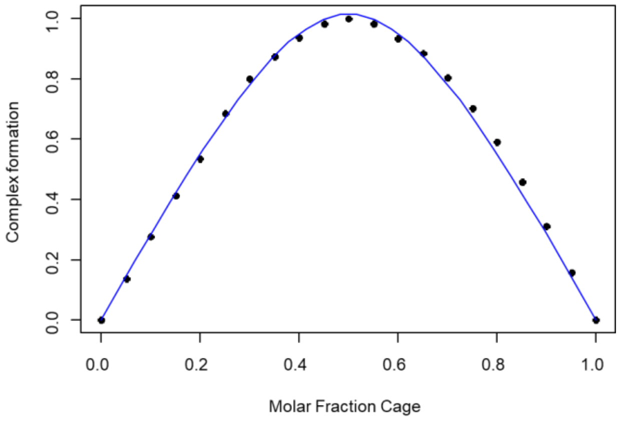

Figure S30. Job plot obtained from the fluorescence emission spectra (H_2O with 10% DMSO and phosphate buffer 1mM, pH 7.1, rt) of the titration of doxorubicin with cage **C-2** (total concentration 50 µM). The blue line represents the fitting to the 1:1 Host–Guest binding model.

A host-guest release experiment was carried out on a JASCO FP- 8300 spectrofluorometer with an excitation wavelength of 470 nm and the emission spectra were recorded in the 510–700 nm range. Two 50 µM stock solutions of DOXO were prepared with phosphate buffer (without NaCl) at pH 7.2 and 6% of

DMSO (one was used as reference). In one of the solutions, the 1.0 equivalent of **C2** was added to completely encapsulate the DOXO inside the cage's cavity. To perform the release experiment, an increasing amount of DMSO was added (6–55%) reducing the hydrophobic effect, as well as producing a dilution of the samples from 50 µM to 25 µM. The fluorescence spectra were recorded after each addition of DMSO (Figure S31).

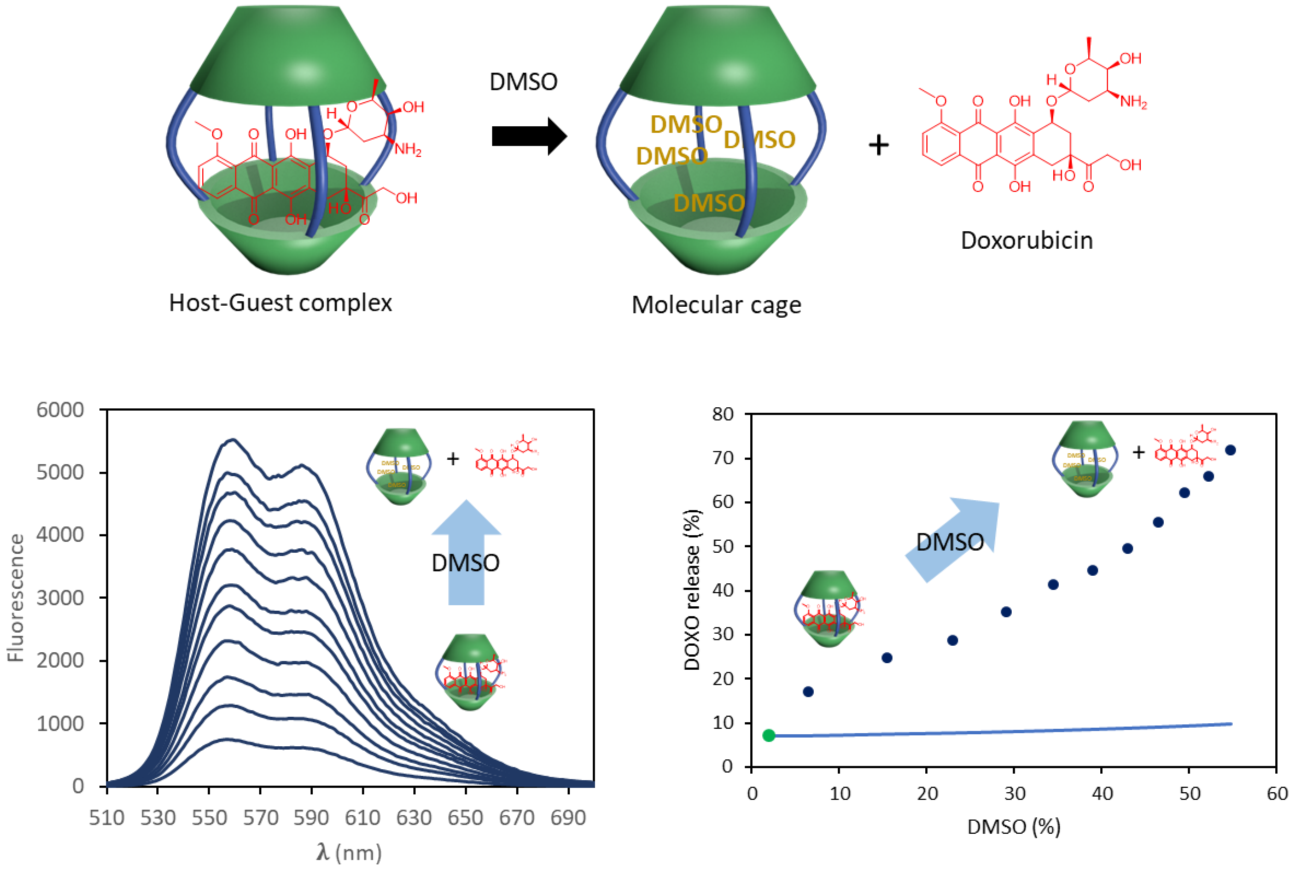

Figure S31. Doxorubicin release of cage encapsulated doxorubicin. Experiment conditions: equimolar solution containing cage (50 µM) and doxorubicin (50 µM) in phosphate buffer (1 mM, pH 7.2) with increasing amounts of DMSO from 6% to 55%. The green dot indicates the release at 2% DMSO as determined in the binding experiments. The blue line indicates the expected release changes by dilution from 50 µM to 25 µM in a solution containing 2% DMSO.

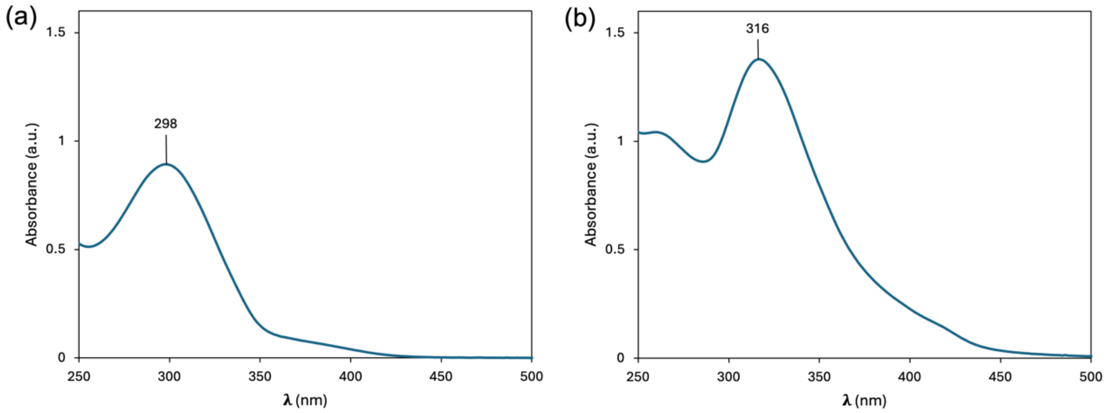

Figure S32. (a) Absorbance spectrum (H_2O with 5% DMSO and phosphate buffer 100 µM, pH 7.1, rt) of cage **C1·NO**$_3$ (20 µM). (b) Absorbance spectrum (H_2O with 5% DMSO and phosphate buffer 100 µM, pH 7.1, rt) of cage **C2** (20 µM).

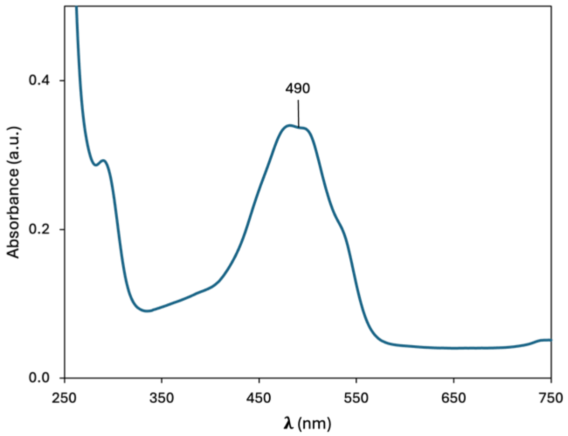

Figure S33. Absorbance spectrum (H_2O with 5% DMSO and phosphate buffer 100 µM, pH 7.1, rt) of doxorubicin (50 µM).

6. Rehm–Weller calculations

For photo-induced electron transfer between an acceptor and a donor the change in standard Gibbs energy can be approximated as described in the Rehm–Weller equation.[S17]

$$\Delta G^\circ = N_A\, e\, ([E^\circ_{(D+\bullet/D)} - E^\circ_{(A/A-\bullet)}] - SE) \qquad \text{Eq S1}$$

In Eq 1 e is the elementary charge, N_A is the Avogadro constant, $E^\circ_{(D+\bullet/D)}$ is the standard electrode potential of the donor cation radical resulting from the electron transfer, $E^\circ_{(A/A-\bullet)}$ is the standard electrode potential of the acceptor and SE is the energy difference between the fundamental electronic state and the first singlet excited state in eV.

The singlet energy excitation of doxorubicin is 2.54 eV,[S18] the reduction potential of doxorubicin is –0.45 V vs. Ag/AgCl/KCl (3 M),[S19] the oxidation potential of resorcinol is observed over a potential range of 1.00–1.50 V vs. RHE,[S 20] reduction potential of tetrakis(pyridine)palladium(II) ($[Pd(Py)_4]^{2+}$) is −1.365 V vs. Fc/Fc^+,[S21] oxidation potential of doxorubicin -0.67 V vs. Ag/AgCl.[S22]

For an estimation of the ΔG° associated to a PET process, the Rehm–Weller equation was used (Eq S1). Calculations were done using values obtained from the literature to the constituent parts of the cages and hence there is some degree of uncertainty in the calculations. Nevertheless, the high exergonicity of the calculated ΔG° is qualitatively indicative of the feasibility of a PET process.

The obtained ΔG° values for the photoinduced electron transfer were -120 kJ/mol for the PET from resorcinol to doxorubicin, and -84 kJ/mol for the PET from doxorubicin to tetrakis(pyridine)palladium(II).

7. Molecular Modelling

The structure of cage **C2** and the supramolecular complex of encapsulated doxorubicin in cage **C2** were modelling with the Spartan' 20 software using the MMFF force field and a standard optimization.[S23]. The geometries and the XYZ coordinates are provided below.

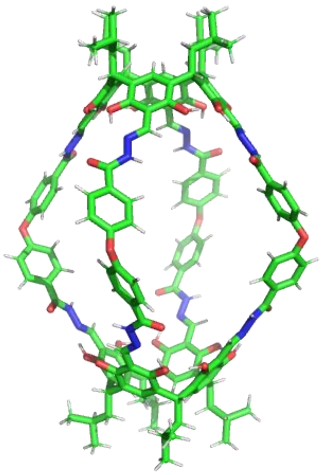

Figure S34. Optimized structure (MMFF, Wavefunction Spartan) of cage **C2**.

XYZ Coordinates of cage **C2**

C	-7.500000	5.442000	6.713000
H	-6.965000	6.344000	7.027000
C	-6.707000	4.258000	7.283000
C	-5.285000	2.067000	8.363000
C	-7.335000	3.018000	7.518000
C	-5.334000	4.362000	7.575000
C	-4.626000	3.281000	8.116000
C	-6.652000	1.921000	8.083000
H	-8.384000	2.903000	7.261000
C	-7.527000	5.403000	5.184000
C	-7.581000	5.355000	2.364000
C	-6.556000	6.088000	4.439000
C	-8.508000	4.672000	4.483000
C	-8.576000	4.657000	3.074000
C	-6.581000	6.071000	3.036000
H	-9.242000	4.106000	5.050000
C	-9.690000	3.889000	2.351000
H	-9.795000	4.365000	1.370000
C	-7.367000	0.600000	8.373000
H	-6.805000	0.065000	9.146000
C	-9.304000	2.425000	2.139000
C	-8.605000	-0.276000	1.727000
C	-8.677000	2.021000	0.951000
C	-9.548000	1.448000	3.126000
C	-9.235000	0.086000	2.933000
C	-8.332000	0.678000	0.739000
H	-9.999000	1.756000	4.065000
C	-7.363000	-0.313000	7.140000
C	-7.405000	-2.001000	4.874000
C	-8.374000	-0.207000	6.161000
C	-6.352000	-1.271000	6.934000
C	-6.372000	-2.115000	5.816000
C	-8.429000	-1.057000	5.038000
H	-9.142000	0.552000	6.279000
C	-9.560000	-0.955000	4.013000
H	-9.641000	-1.914000	3.491000
C	-10.976000	-0.795000	4.638000
H	-11.062000	0.158000	5.173000
H	-11.710000	-0.743000	3.822000
C	-8.768000	0.767000	9.030000
H	-9.196000	-0.232000	9.196000
H	-9.461000	1.274000	8.347000
C	-8.900000	5.636000	7.369000
H	-8.773000	5.650000	8.461000
H	-9.550000	4.779000	7.159000
C	-11.103000	4.094000	2.973000
H	-11.143000	3.694000	3.993000
H	-11.284000	5.174000	3.072000
O	-8.403000	2.998000	0.019000
H	-7.989000	2.564000	-0.769000
O	-7.551000	5.364000	0.994000
H	-7.915000	4.520000	0.649000
O	-8.235000	-1.572000	1.472000
H	-8.004000	-2.022000	2.313000
O	-7.450000	-2.790000	3.746000
C	9.728000	2.258000	-5.846000
H	9.254000	3.009000	-6.487000
C	11.337000	-2.756000	-0.877000
H	11.358000	-3.447000	-0.028000
C	9.779000	2.805000	-4.419000
C	9.876000	3.837000	-1.795000
C	8.876000	3.797000	-4.005000
C	10.714000	2.326000	-3.478000
C	10.803000	2.846000	-2.170000
C	8.923000	4.316000	-2.703000
H	11.394000	1.532000	-3.776000
C	11.110000	-1.360000	-0.281000
C	10.731000	1.223000	0.802000
C	11.524000	-0.204000	-0.975000
C	10.472000	-1.185000	0.961000
C	10.287000	0.093000	1.505000
C	11.370000	1.092000	-0.441000
H	11.984000	-0.313000	-1.953000
C	11.866000	2.330000	-1.191000
H	12.021000	3.134000	-0.465000
C	13.280000	2.171000	-1.820000
H	13.274000	1.410000	-2.609000
H	13.543000	3.114000	-2.321000
C	12.749000	-2.952000	-1.505000
H	13.504000	-2.639000	-0.770000
H	12.886000	-2.291000	-2.369000
C	10.595000	-3.033000	-6.178000
H	10.941000	-4.049000	-5.942000
H	11.335000	-2.359000	-5.731000
C	11.123000	2.083000	-6.514000
H	11.712000	1.318000	-5.994000
H	10.973000	1.698000	-7.533000
O	7.938000	4.218000	-4.922000
H	7.393000	4.930000	-4.500000
O	6.869000	2.309000	-6.498000
H	7.304000	3.007000	-5.961000
O	9.870000	4.372000	-0.533000
H	10.170000	3.698000	0.114000
O	10.546000	2.499000	1.284000
H	10.110000	2.433000	2.171000
O	10.009000	-2.252000	1.687000
H	9.733000	-2.972000	1.078000
O	9.098000	-4.186000	0.045000
H	8.286000	-4.719000	0.244000
O	7.004000	-4.314000	-4.271000
H	6.858000	-3.660000	-4.989000
O	6.481000	-2.462000	-6.164000
H	5.560000	-2.216000	-6.436000
C	7.959000	5.344000	-2.253000
H	8.052000	5.732000	-1.224000
C	9.592000	0.226000	2.805000
H	9.279000	-0.696000	3.326000
C	6.876000	-5.022000	-1.618000
H	6.085000	-5.318000	-2.328000
C	5.238000	0.104000	-6.685000
C	-1.889000	-0.789000	-7.584000
C	0.825000	-1.363000	-7.238000
C	-1.049000	0.153000	-6.984000
C	-1.375000	-2.002000	-8.039000
C	-0.022000	-2.295000	-7.855000
C	0.304000	-0.138000	-6.797000
H	-1.444000	1.109000	-6.646000
H	-2.028000	-2.727000	-8.520000
H	0.371000	-3.253000	-8.189000
H	0.922000	0.588000	-6.277000
C	-3.948000	-0.095000	-6.707000
C	-5.468000	0.914000	-4.589000
C	-4.793000	0.994000	-6.918000
C	-3.880000	-0.703000	-5.450000
C	-4.628000	-0.191000	-4.387000
C	-5.542000	1.507000	-5.857000
H	-4.856000	1.454000	-7.901000
H	-3.238000	-1.564000	-5.287000
H	-4.516000	-0.646000	-3.407000
H	-6.183000	2.372000	-6.019000
C	2.251000	-1.735000	-7.026000
C	-6.258000	1.515000	-3.480000
O	2.553000	-2.918000	-6.893000
O	-6.504000	2.718000	-3.495000
N	3.152000	-0.682000	-7.002000
H	2.868000	0.266000	-7.221000
N	4.459000	-0.933000	-6.722000
N	-6.672000	0.636000	-2.492000
H	-6.530000	-0.365000	-2.576000
N	-7.325000	1.123000	-1.405000
O	1.394000	10.660000	-0.906000
C	2.310000	9.780000	-1.439000
C	4.169000	8.063000	-2.625000
C	2.982000	8.836000	-0.658000
C	2.584000	9.891000	-2.802000
C	3.503000	9.024000	-3.397000
C	3.900000	7.967000	-1.252000
H	2.786000	8.761000	0.409000
H	2.071000	10.637000	-3.403000
H	3.697000	9.097000	-4.466000
H	4.367000	7.205000	-0.634000
C	0.420000	10.120000	-0.096000
C	-1.549000	9.125000	1.619000
C	0.177000	10.755000	1.121000
C	-0.336000	9.007000	-0.475000
C	-1.311000	8.499000	0.387000
C	-0.798000	10.250000	1.985000
H	0.756000	11.630000	1.406000
H	-0.162000	8.522000	-1.433000
H	-1.845000	7.601000	0.093000
H	-0.970000	10.734000	2.945000
C	5.115000	7.138000	-3.306000
C	-2.555000	8.601000	2.584000
O	4.931000	6.843000	-4.484000
O	-2.379000	8.760000	3.789000
N	9.346000	1.400000	3.299000
N	-1.158000	2.663000	9.004000
H	-0.794000	3.597000	8.850000
N	-2.484000	2.431000	8.823000
C	14.406000	1.814000	-0.818000
H	14.189000	0.839000	-0.365000
C	15.737000	1.679000	-1.562000
H	16.536000	1.374000	-0.877000
H	16.032000	2.628000	-2.024000
H	15.669000	0.922000	-2.350000
C	14.553000	2.835000	0.312000
H	15.421000	2.601000	0.938000
H	13.679000	2.834000	0.970000
H	14.685000	3.848000	-0.083000
C	13.065000	-4.402000	-1.952000
H	12.331000	-4.719000	-2.702000
C	14.441000	-4.448000	-2.623000
H	15.234000	-4.158000	-1.924000
H	14.478000	-3.770000	-3.482000
H	14.662000	-5.457000	-2.987000
C	13.024000	-5.405000	-0.798000
H	13.686000	-5.098000	0.019000
H	13.342000	-6.398000	-1.135000
H	12.012000	-5.514000	-0.397000
C	10.633000	-2.841000	-7.715000
H	10.311000	-1.822000	-7.961000
C	12.070000	-2.993000	-8.220000
H	12.733000	-2.271000	-7.731000
H	12.123000	-2.814000	-9.299000
H	12.456000	-3.999000	-8.023000
C	9.721000	-3.817000	-8.461000
H	9.948000	-4.855000	-8.194000
H	9.845000	-3.712000	-9.544000
H	8.666000	-3.629000	-8.242000
C	11.977000	3.374000	-6.594000
H	12.194000	3.726000	-5.579000
C	13.320000	3.063000	-7.261000
H	13.855000	2.280000	-6.715000
H	13.959000	3.953000	-7.278000
H	13.182000	2.726000	-8.294000
C	11.282000	4.509000	-7.348000
H	11.956000	5.365000	-7.469000
H	10.405000	4.873000	-6.805000
H	10.961000	4.188000	-8.344000
C	-8.768000	1.536000	10.375000
H	-8.363000	2.542000	10.218000
C	-10.206000	1.702000	10.876000
H	-10.228000	2.284000	11.804000
H	-10.673000	0.731000	11.073000
H	-10.816000	2.232000	10.138000
C	-7.926000	0.851000	11.453000
H	-6.863000	0.858000	11.197000
H	-8.238000	-0.189000	11.601000
H	-8.024000	1.372000	12.412000
C	-9.645000	6.925000	6.940000

H	-6.687000	-3.421000	3.780000
O	-5.313000	-1.418000	7.817000
H	-5.109000	-0.553000	8.235000
O	-4.624000	0.970000	8.869000
H	-3.683000	1.229000	9.039000
O	-4.638000	5.521000	7.345000
H	-5.024000	5.992000	6.575000
O	-5.578000	6.757000	5.141000
H	-4.984000	7.207000	4.488000
C	-7.651000	0.250000	-0.503000
H	-7.418000	-0.822000	-0.627000
C	-5.276000	-3.093000	5.636000
H	-4.496000	-3.152000	6.415000
C	-3.179000	3.434000	8.385000
H	-2.713000	4.415000	8.187000
C	-5.546000	6.771000	2.244000
H	-5.623000	6.742000	1.143000
C	9.195000	-2.825000	-5.527000
H	8.581000	-3.570000	-6.045000
C	9.142000	-3.193000	-4.039000
C	9.094000	-3.886000	-1.299000
C	10.172000	-2.797000	-3.162000
C	8.065000	-3.921000	-3.497000
C	8.040000	-4.273000	-2.141000
C	10.183000	-3.157000	-1.798000
H	10.993000	-2.201000	-3.550000
C	8.590000	-1.443000	-5.776000
C	7.471000	1.101000	-6.259000
C	7.233000	-1.310000	-6.109000
C	9.364000	-0.269000	-5.664000
C	8.837000	1.011000	-5.930000
C	6.673000	-0.047000	-6.355000
H	10.406000	-0.358000	-5.370000
H	4.853000	1.120000	-6.882000
O	0.604000	-8.682000	2.908000
C	1.766000	-8.074000	2.489000
C	4.185000	-6.906000	1.715000
C	1.807000	-7.231000	1.374000
C	2.931000	-8.360000	3.200000
C	4.137000	-7.766000	2.821000
C	3.012000	-6.635000	0.995000
H	0.903000	-7.022000	0.807000
H	2.898000	-9.028000	4.056000
H	5.041000	-7.972000	3.392000
H	3.006000	-5.940000	0.161000
C	-0.499000	-7.869000	3.053000
C	-2.806000	-6.311000	3.301000
C	-1.699000	-8.317000	2.504000
C	-0.450000	-6.660000	3.753000
C	-1.599000	-5.873000	3.865000
C	-2.849000	-7.533000	2.616000
H	-1.738000	-9.266000	1.974000
H	0.480000	-6.317000	4.200000
H	-1.521000	-4.912000	4.365000
H	-3.780000	-7.874000	2.166000
C	5.485000	-6.268000	1.370000
C	-4.046000	-5.491000	3.367000
O	6.308000	-6.054000	2.255000
O	-4.872000	-5.569000	2.462000
N	5.668000	-5.966000	0.029000
H	5.002000	-6.257000	-0.677000
N	6.791000	-5.304000	-0.354000
N	-4.175000	-4.685000	4.488000
H	-3.515000	-4.733000	5.256000
N	-5.237000	-3.843000	4.579000
O	-3.231000	-0.556000	-7.788000
N	6.167000	6.676000	-2.530000
H	6.331000	7.031000	-1.594000
N	7.022000	5.758000	-3.050000
N	-3.651000	7.964000	2.022000
H	-3.801000	7.949000	1.020000
N	-4.568000	7.382000	2.838000
O	5.222000	2.602000	9.768000
C	5.958000	2.561000	8.605000
C	7.523000	2.568000	6.289000
C	5.833000	1.524000	7.677000
C	6.882000	3.585000	8.397000
C	7.655000	3.596000	7.234000
C	6.603000	1.534000	6.512000
H	5.128000	0.713000	7.846000
H	6.989000	4.381000	9.129000
H	8.359000	4.409000	7.063000
H	6.446000	0.746000	5.781000
C	3.865000	2.405000	9.647000
C	1.112000	1.941000	9.506000
C	3.267000	1.508000	10.532000
C	3.090000	3.094000	8.710000
C	1.716000	2.853000	8.628000
C	1.894000	1.266000	10.453000
H	3.870000	0.986000	11.270000
H	3.549000	3.809000	8.032000
H	1.146000	3.360000	7.855000
H	1.433000	0.549000	11.130000
C	8.343000	2.637000	5.049000
C	-0.342000	1.629000	9.434000
O	8.683000	3.732000	4.607000
O	-0.738000	0.511000	9.755000
N	8.674000	1.418000	4.479000
H	8.457000	0.540000	4.938000
H	-9.797000	6.912000	5.854000
C	-11.032000	6.959000	7.588000
H	-10.961000	6.992000	8.680000
H	-11.614000	6.075000	7.309000
H	-11.592000	7.842000	7.258000
C	-8.882000	8.202000	7.295000
H	-7.952000	8.286000	6.725000
H	-8.635000	8.234000	8.361000
H	-9.480000	9.090000	7.060000
C	-12.269000	3.469000	2.166000
H	-12.136000	2.382000	2.123000
C	-13.594000	3.733000	2.886000
H	-13.572000	3.332000	3.905000
H	-14.425000	3.253000	2.358000
H	-13.807000	4.806000	2.946000
C	-12.355000	3.990000	0.730000
H	-11.495000	3.670000	0.135000
H	-12.402000	5.084000	0.706000
H	-13.248000	3.602000	0.229000
C	-11.395000	-1.930000	5.607000
H	-10.709000	-1.944000	6.463000
C	-11.367000	-3.316000	4.960000
H	-10.347000	-3.621000	4.709000
H	-11.967000	-3.339000	4.044000
H	-11.765000	-4.073000	5.644000
C	-12.797000	-1.650000	6.156000
H	-12.831000	-0.679000	6.661000
H	-13.088000	-2.414000	6.884000
H	-13.543000	-1.644000	5.354000

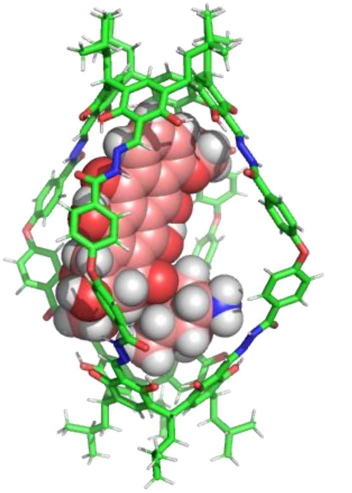

Figure S35. Optimized structure (MMFF, Wavefunction Spartan) of doxorubicin⊂**C2** complex.

XYZ Coordinates of cage doxorubicin⊂**C2** complex

C	-4.026000	1.576000	4.690000
C	-4.630000	2.825000	4.596000
C	-4.191000	3.741000	3.640000
C	-3.151000	3.412000	2.759000
O	-2.648000	4.287000	1.834000
C	-2.550000	2.138000	2.843000
C	-1.469000	1.709000	1.916000
O	-1.174000	2.345000	0.909000
C	-0.706000	0.466000	2.185000
C	0.451000	0.152000	1.439000
O	0.846000	0.961000	0.402000
C	1.221000	-1.001000	1.706000
C	2.495000	-1.336000	0.931000
O	3.287000	-0.175000	0.639000
C	3.411000	-2.254000	1.753000
C	2.699000	-3.542000	2.147000
O	3.584000	-4.319000	2.984000
C	2.418000	-4.390000	0.884000
O	1.277000	-4.565000	0.449000
C	3.643000	-4.969000	0.169000
O	3.767000	-4.495000	-1.154000
C	1.443000	-3.219000	2.975000
C	0.740000	-1.906000	2.679000
C	-0.423000	-1.589000	3.412000
O	-0.812000	-2.488000	4.376000
C	-11.035000	3.428000	4.242000
H	-10.775000	4.018000	5.130000
H	-11.559000	4.124000	3.571000
C	-9.025000	1.956000	8.710000
H	-9.856000	1.288000	8.443000
H	-9.315000	2.948000	8.345000
C	-6.812000	6.553000	9.274000
H	-6.836000	6.053000	10.253000
H	-7.731000	6.242000	8.764000
C	-8.880000	8.057000	4.857000
H	-9.208000	7.313000	5.593000
H	-8.585000	8.940000	5.442000
O	-6.609000	7.259000	1.471000
H	-6.341000	7.065000	0.536000
O	-4.901000	8.301000	3.276000
H	-5.601000	8.009000	2.653000
O	-8.550000	2.914000	0.846000
H	-8.593000	2.103000	1.398000
O	-8.494000	0.695000	2.353000
H	-8.043000	-0.140000	2.065000
O	-6.627000	-0.825000	6.501000
H	-6.181000	-0.420000	7.276000
O	-5.201000	0.334000	8.441000
H	-4.270000	0.051000	8.632000
O	-3.041000	4.593000	8.895000
C	3.968000	-5.537000	-4.376000
H	3.259000	-5.106000	-5.104000
C	5.463000	1.378000	-6.338000
H	5.545000	2.404000	-5.938000
O	-3.902000	-7.316000	-1.456000
C	-2.549000	-7.248000	-1.701000
C	0.212000	-7.194000	-2.109000
C	-1.965000	-6.164000	-2.363000
C	-1.768000	-8.324000	-1.282000
C	-0.386000	-8.292000	-1.475000
C	-0.582000	-6.127000	-2.552000
H	-2.574000	-5.332000	-2.707000
H	-2.228000	-9.172000	-0.782000
H	0.227000	-9.117000	-1.118000
H	-0.148000	-5.238000	-2.999000
C	-4.483000	-6.197000	-0.901000
C	-5.763000	-3.943000	0.143000
C	-5.685000	-5.765000	-1.458000
C	-3.931000	-5.526000	0.196000
C	-4.562000	-4.390000	0.710000
C	-6.318000	-4.631000	-0.946000
H	-6.120000	-6.297000	-2.300000
H	-3.001000	-5.869000	0.643000
H	-4.085000	-3.857000	1.527000
H	-7.246000	-4.283000	-1.397000
H	3.426000	-3.758000	6.881000
H	6.599000	-2.439000	9.483000
H	8.105000	-2.215000	7.527000
H	4.884000	-3.483000	4.940000
C	2.845000	-2.660000	9.309000
C	0.225000	-1.723000	9.075000
C	1.786000	-3.460000	9.736000
C	2.606000	-1.380000	8.799000
C	1.297000	-0.918000	8.664000
C	0.475000	-2.996000	9.608000
H	1.977000	-4.448000	10.146000
H	3.434000	-0.749000	8.484000
H	1.138000	0.054000	8.206000
H	-0.353000	-3.630000	9.919000
C	7.541000	-2.691000	4.945000
C	-1.190000	-1.282000	8.931000
O	8.462000	-1.881000	5.021000
O	-2.077000	-2.120000	8.797000
N	7.296000	-3.504000	3.849000
H	6.573000	-4.215000	3.861000
N	8.097000	-3.395000	2.758000
N	-1.398000	0.087000	8.981000
H	-0.649000	0.730000	9.214000
N	-2.654000	0.571000	8.811000
C	9.822000	-7.666000	-4.568000

C	-1.139000	-0.406000	3.175000
C	-2.358000	-0.092000	3.943000
O	-2.830000	-0.943000	4.692000
C	-2.988000	1.234000	3.815000
C	-3.381000	5.473000	1.561000
C	3.421000	0.030000	-0.773000
C	3.858000	1.468000	-1.055000
C	5.321000	1.693000	-0.681000
N	5.704000	3.064000	-1.048000
C	6.206000	0.618000	-1.351000
O	6.211000	0.803000	-2.772000
C	5.669000	-0.776000	-0.971000
O	4.299000	-0.932000	-1.353000
C	6.448000	-1.900000	-1.646000
H	-4.362000	0.872000	5.450000
H	-5.437000	3.095000	5.273000
H	-4.676000	4.712000	3.615000
H	0.203000	1.702000	0.309000
H	2.188000	-1.850000	0.011000
H	3.769000	-1.731000	2.653000
H	4.327000	-2.467000	1.190000
H	3.061000	-5.070000	3.327000
H	4.560000	-4.699000	0.699000
H	3.577000	-6.060000	0.180000
H	3.270000	-5.115000	-1.727000
H	1.732000	-3.188000	4.036000
H	0.719000	-4.039000	2.881000
H	-1.642000	-2.166000	4.800000
H	-2.936000	5.952000	0.684000
H	-4.423000	5.247000	1.326000
H	-3.306000	6.170000	2.398000
H	2.444000	-0.106000	-1.252000
H	3.723000	1.669000	-2.126000
H	3.221000	2.168000	-0.503000
H	5.434000	1.597000	0.407000
H	5.269000	3.739000	-0.426000
H	6.717000	3.194000	-1.004000
H	7.238000	0.724000	-1.003000
H	6.842000	0.170000	-3.151000
H	5.753000	-0.909000	0.114000
H	6.117000	-2.870000	-1.258000
H	6.269000	-1.926000	-2.726000
H	7.522000	-1.801000	-1.466000
C	-5.554000	6.057000	8.501000
H	-4.729000	6.420000	9.123000
C	-5.428000	4.528000	8.472000
C	-5.250000	1.710000	8.471000
C	-6.556000	3.718000	8.223000
C	-4.194000	3.885000	8.683000
C	-4.103000	2.488000	8.687000
C	-6.497000	2.311000	8.244000
H	-7.509000	4.196000	8.012000
C	-5.397000	6.648000	7.099000
C	-5.109000	7.763000	4.520000
C	-4.139000	7.056000	6.631000
C	-6.499000	6.784000	6.232000
C	-6.389000	7.369000	4.953000
C	-3.989000	7.612000	5.350000
H	-7.470000	6.435000	6.570000
C	-7.634000	7.549000	4.073000
H	-7.414000	8.388000	3.405000
C	-7.740000	1.455000	7.988000
H	-7.581000	0.467000	8.433000
C	-7.889000	6.303000	3.224000
C	-8.362000	4.007000	1.651000
C	-7.375000	6.207000	1.923000
C	-8.622000	5.210000	3.726000
C	-8.899000	4.065000	2.949000
C	-7.610000	5.069000	1.135000
H	-8.994000	5.255000	4.746000
C	-7.933000	1.221000	6.484000
C	-8.305000	0.836000	3.710000
C	-8.671000	2.137000	5.705000
C	-7.366000	0.112000	5.828000
C	-7.539000	-0.078000	4.451000
C	-8.901000	1.946000	4.328000
H	-9.088000	3.018000	6.183000
C	-9.753000	2.923000	3.517000
H	-10.162000	2.384000	2.656000
H	-3.086000	5.456000	8.428000
O	-3.066000	6.869000	7.473000
H	-2.254000	7.217000	7.024000
C	-7.024000	4.938000	-0.217000
H	-7.265000	4.037000	-0.809000
C	-6.875000	-1.229000	3.797000
H	-6.293000	-1.927000	4.424000
C	-2.779000	1.859000	8.883000
H	-1.911000	2.514000	9.077000
C	-2.656000	8.011000	4.842000
H	-2.590000	8.469000	3.840000
C	7.615000	-3.159000	-6.936000
H	6.849000	-3.244000	-7.714000
C	7.159000	-4.054000	-5.776000
C	6.371000	-5.750000	-3.660000
C	8.095000	-4.556000	-4.849000
C	5.806000	-4.396000	-5.592000
C	5.409000	-5.239000	-4.545000
C	7.729000	-5.428000	-3.806000
H	9.137000	-4.268000	-4.947000
C	7.694000	-1.695000	-6.500000
C	7.840000	1.017000	-5.730000
C	6.583000	-0.850000	-6.638000
C	8.866000	-1.156000	-5.932000
C	8.976000	0.203000	-5.571000
C	6.648000	0.498000	-6.250000
H	9.721000	-1.810000	-5.784000
C	10.291000	0.766000	-5.020000
H	10.282000	1.838000	-5.244000
C	8.776000	-5.992000	-2.844000
H	8.368000	-6.890000	-2.367000
C	10.373000	0.588000	-3.502000
C	10.551000	0.271000	-0.704000
C	9.970000	1.618000	-2.638000
C	10.835000	-0.613000	-2.929000
C	10.962000	-0.787000	-1.535000
C	10.065000	1.470000	-1.245000
H	11.109000	-1.431000	-3.589000
C	9.067000	-5.006000	-1.705000
C	9.685000	-3.224000	0.395000
C	10.073000	-4.024000	-1.842000
C	8.343000	-5.039000	-0.500000
C	8.651000	-4.161000	0.547000
C	10.416000	-3.141000	-0.800000
H	10.608000	-3.949000	-2.784000
C	11.523000	-2.098000	-0.964000
H	11.917000	-1.857000	0.029000
C	12.787000	-2.600000	-1.723000
H	12.542000	-2.862000	-2.758000
H	13.501000	-1.767000	-1.793000
C	10.059000	-6.528000	-3.547000
H	10.753000	-6.890000	-2.775000
H	10.588000	-5.718000	-4.062000
C	8.893000	-3.695000	-7.649000
H	8.732000	-4.754000	-7.898000
H	9.753000	-3.683000	-6.968000
C	11.547000	0.263000	-5.791000
H	11.672000	-0.820000	-5.669000
H	11.379000	0.420000	-6.866000
O	9.459000	2.756000	-3.218000
H	9.223000	3.398000	-2.502000
O	7.866000	2.346000	-5.398000
H	8.417000	2.477000	-4.596000
O	10.599000	0.172000	0.662000
H	10.479000	-0.765000	0.932000
O	10.011000	-2.333000	1.394000
H	9.450000	-2.539000	2.185000
O	7.314000	-5.924000	-0.302000
H	6.894000	-6.143000	-1.162000
O	6.030000	-6.567000	-2.606000
H	5.055000	-6.727000	-2.653000
O	4.832000	-3.924000	-6.432000
H	5.069000	-3.016000	-6.726000
O	5.429000	-1.403000	-7.148000
H	4.753000	-0.682000	-7.221000
C	9.613000	2.538000	-0.325000
H	9.747000	2.387000	0.760000
C	7.846000	-4.216000	1.787000
H	7.035000	-4.962000	1.854000
C	1.694000	-7.168000	-2.230000
C	-6.467000	-2.730000	0.639000
O	2.384000	-7.763000	-1.406000
O	-7.132000	-2.054000	-0.139000
N	2.209000	-6.431000	-3.286000
H	1.608000	-6.033000	-4.002000
N	3.555000	-6.263000	-3.380000
N	-6.334000	-2.468000	1.995000
H	-5.868000	-3.117000	2.620000
N	-6.978000	-1.391000	2.515000
O	-2.164000	5.143000	-7.237000
C	-1.111000	4.255000	-7.208000
C	1.003000	2.425000	-7.292000
C	-0.093000	4.320000	-6.252000
C	-1.064000	3.296000	-8.219000
C	-0.015000	2.375000	-8.255000
C	0.955000	3.396000	-6.283000
H	-0.112000	5.079000	-5.473000
H	-1.847000	3.256000	-8.972000
H	0.010000	1.617000	-9.036000
H	1.703000	3.438000	-5.496000
C	-2.785000	5.415000	-6.040000
C	-4.071000	6.087000	-3.652000
C	-3.025000	6.755000	-5.736000
C	-3.208000	4.406000	-5.170000
C	-3.837000	4.741000	-3.968000
C	-3.656000	7.092000	-4.536000
H	-2.708000	7.535000	-6.422000
H	-3.038000	3.360000	-5.413000
H	-4.104000	3.942000	-3.283000
H	-3.823000	8.139000	-4.291000
C	2.096000	1.415000	-7.370000
C	-4.717000	6.490000	-2.374000
O	1.874000	0.322000	-7.883000
O	-4.401000	7.547000	-1.837000
N	3.321000	1.814000	-6.859000
H	3.476000	2.755000	-6.515000
N	4.358000	0.937000	-6.857000
N	-5.667000	5.609000	-1.881000
H	-5.942000	4.780000	-2.397000
N	-6.219000	5.850000	-0.664000
O	5.715000	9.266000	3.375000
C	6.289000	8.358000	2.515000
C	7.504000	6.613000	0.696000
C	6.376000	6.993000	2.808000
C	6.836000	8.859000	1.335000
C	7.432000	7.987000	0.421000
C	6.971000	6.118000	1.894000
H	5.972000	6.602000	3.738000
H	6.783000	9.922000	1.115000
H	7.831000	8.379000	-0.513000
H	6.972000	5.057000	2.123000
C	4.485000	8.934000	3.899000
C	2.023000	8.323000	5.073000
C	4.319000	9.084000	5.275000
C	3.419000	8.510000	3.100000
C	2.190000	8.192000	3.687000
C	3.093000	8.765000	5.863000
H	5.144000	9.431000	5.891000
H	3.540000	8.414000	2.024000
H	1.394000	7.814000	3.053000
H	2.973000	8.860000	6.941000
C	8.094000	5.719000	-0.339000
C	0.749000	7.964000	5.752000
O	8.081000	6.067000	-1.516000
O	0.778000	7.536000	6.903000
N	8.632000	4.523000	0.117000
H	8.769000	4.342000	1.105000
N	9.055000	3.610000	-0.795000
N	-0.410000	8.162000	5.016000
H	-0.395000	8.612000	4.107000
N	-1.600000	7.802000	5.564000
O	4.118000	-3.174000	9.426000
C	4.914000	-3.090000	8.305000
C	6.614000	-2.860000	6.098000
C	4.449000	-3.419000	7.029000
C	6.234000	-2.679000	8.488000
C	7.081000	-2.555000	7.384000
C	5.291000	-3.284000	5.925000
H	9.148000	-7.312000	-5.357000
C	11.149000	-8.039000	-5.237000
H	11.871000	-8.416000	-4.505000
H	11.591000	-7.170000	-5.736000
H	10.998000	-8.815000	-5.995000
C	9.200000	-8.913000	-3.936000
H	9.791000	-9.262000	-3.083000
H	9.142000	-9.730000	-4.664000
H	8.180000	-8.720000	-3.592000
C	9.295000	-2.943000	-8.940000
H	9.480000	-1.888000	-8.704000
C	10.604000	-3.523000	-9.484000
H	11.403000	-3.447000	-8.739000
H	10.930000	-2.978000	-10.377000
H	10.489000	-4.578000	-9.755000
C	8.217000	-3.006000	-10.023000
H	8.576000	-2.553000	-10.954000
H	7.319000	-2.456000	-9.726000
H	7.931000	-4.040000	-10.239000
C	12.880000	0.942000	-5.397000
H	13.083000	0.753000	-4.336000
C	14.028000	0.320000	-6.198000
H	14.989000	0.754000	-5.903000
H	13.898000	0.487000	-7.273000
H	14.084000	-0.760000	-6.025000
C	12.866000	2.456000	-5.615000
H	13.856000	2.885000	-5.428000
H	12.170000	2.952000	-4.932000
H	12.578000	2.707000	-6.642000
C	13.507000	-3.810000	-1.081000
H	12.820000	-4.664000	-1.046000
C	14.696000	-4.219000	-1.956000
H	15.436000	-3.414000	-2.023000
H	14.367000	-4.465000	-2.971000
H	15.195000	-5.103000	-1.545000
C	13.990000	-3.530000	0.342000
H	14.610000	-2.628000	0.382000
H	14.586000	-4.367000	0.722000
H	13.150000	-3.400000	1.031000
C	-8.927000	2.027000	10.253000
H	-8.115000	2.708000	10.535000
C	-10.225000	2.607000	10.823000
H	-10.163000	2.707000	11.911000
H	-11.083000	1.967000	10.589000
H	-10.421000	3.603000	10.410000
C	-8.649000	0.667000	10.895000
H	-7.653000	0.298000	10.635000
H	-9.385000	-0.080000	10.578000
H	-8.689000	0.737000	11.987000
C	-6.885000	8.081000	9.502000
H	-6.884000	8.594000	8.532000
C	-8.201000	8.433000	10.201000
H	-8.294000	9.516000	10.335000
H	-8.265000	7.962000	11.188000
H	-9.059000	8.098000	9.607000
C	-5.710000	8.617000	10.322000
H	-4.767000	8.529000	9.776000
H	-5.609000	8.078000	11.270000
H	-5.848000	9.679000	10.551000
C	-10.102000	8.431000	3.984000
H	-10.438000	7.544000	3.433000
C	-11.259000	8.872000	4.886000
H	-10.998000	9.769000	5.457000
H	-11.524000	8.081000	5.595000
H	-12.152000	9.097000	4.291000
C	-9.796000	9.533000	2.969000
H	-9.093000	9.189000	2.204000
H	-9.368000	10.415000	3.457000
H	-10.707000	9.844000	2.446000
C	-12.029000	2.324000	4.677000
H	-11.537000	1.654000	5.391000
C	-12.535000	1.481000	3.506000
H	-11.732000	0.875000	3.074000
H	-12.954000	2.110000	2.714000
H	-13.316000	0.787000	3.835000
C	-13.219000	2.963000	5.400000
H	-12.883000	3.540000	6.268000
H	-13.914000	2.197000	5.759000
H	-13.772000	3.637000	4.736000

The cavity volume of the cages was determined from the corresponding cage structureds using the CageCavityCalc Python script,[S24] obtaining 1350 Å³ for cage **C1**, and 1500 Å³ for cage **C2**. The the volume of DOXO (498 Å³) was determined with the Wavefunciton Spartan software.

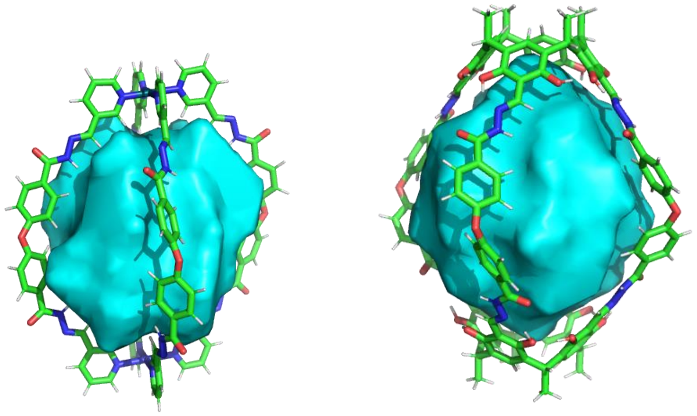

Figure S36. Cavity volume of the cages **C1** (left) and **C2** (right) determined from the corresponding cage structureds using a the CageCavityCalc Python script based on the rolling prove algorithm.

8. Cell viability studies

Cell culture

Murine 4T1 triple-negative breast cancer cells and human melanoma SK-Mel-103 cells were obtained from ATCC and cultured in DMEM medium (Sigma), supplemented with 10% FBS (Sigma). Cells were incubated in an atmosphere of 5% CO_2 at 37 ºC.

Cell viability/Cytotoxicity assays

Both cell lines (4T1 and SK-Mel-103) were plated in 96-well plates at a density of 2500 cells per well. After 24 hours, cells were treated with different concentrations of both organic and metalorganic molecular cages (50 µM, 25 µM, 12.5 µM, 6.25 µM, 3.125 µM, 1.5625 µM and 0.78125 µM). After 48 h of treatment cell proliferation reagent WST-1 (Sigma Aldrich) was added to cells according to manufacturer's instructions. After 1 h of incubation, absorbance at 595 nm was measured in a PerkinElmer Wallac 1420 Victor2 spectrophotometer.

For the assays with the components of the organic cage, SK-Mel-103 cells were plated in 96-well plates at a density of 2500 cells per well. After 24 hours, cells were treated with 50 µM of calixarene, with 100 µM of linker and with both components together. After 48 h of treatment, viability was measured using the WST-1 method.

Drug-encapsulation *in vitro* assay

Cells were plated in 96-well plates at a density of 5000 cells/well. 24 h later they were treated either with free doxorubicin at concentrations 5 µM, 2.5 µM and 1.25 µM or with the complex organic cage-doxorubicin, prepared at a fixed concentration of 25 µM of organic cage and different concentrations of doxorubicin (5, 2.5 and 1.25 µM), resulting in a 95 %, 96 % and 96 % of encapsulation of the drug, respectively. After 24 h of treatment, WST-1 was added to the cells according to manufacturer's instructions and absorbance at 595 nm was measured in Wallac 1420 Victor2 spectrophotometer.

For the assays with the components of the organic cage, SK-Mel-103 cells were plated in 96-well plates at a density of 5000 cells/well. 24 h later they were treated with 50 µM of calixarene, 100 µM of ligand and different concentrations of doxorubicin (5, 2.5 and 1.25 µM). After 24 h of treatment, viability was measured using the WST-1 method.

Internalization of the organic cage-doxorubicin complex

SK-Mel-103 cells were cultured in 6-well plates at 350,000 cells/well for 24 h. Then, cells were incubated with the nuclei marker Hoechst 33342 at a concentration of 1 µg/mL for 30 min. For the time-lapse experiment, the cell membrane marker Wheat Germ Agglutinin, Alexa Fluor™ 647 Conjugate (Invitrogen, W32466) was added at a concentration of 1 µg/mL 10 minutes before starting imaging. After the incubation with markers, cells were washed with PBS (Merck, D8537) and treated either with the organic-cage complex, formed previously with a mix of 25 µM of organic cage and 5 µM of doxorubicin (95 % of encapsulation). Similarly, the cells were treated with free doxorubicin as a control. A time-lapse up to 10

minutes since treatment was performed for every condition in a confocal Leica TCS SP8 HyVolution II microscope, equipped with CO_2 and temperature control and a resonant scanner for live-cells studies.

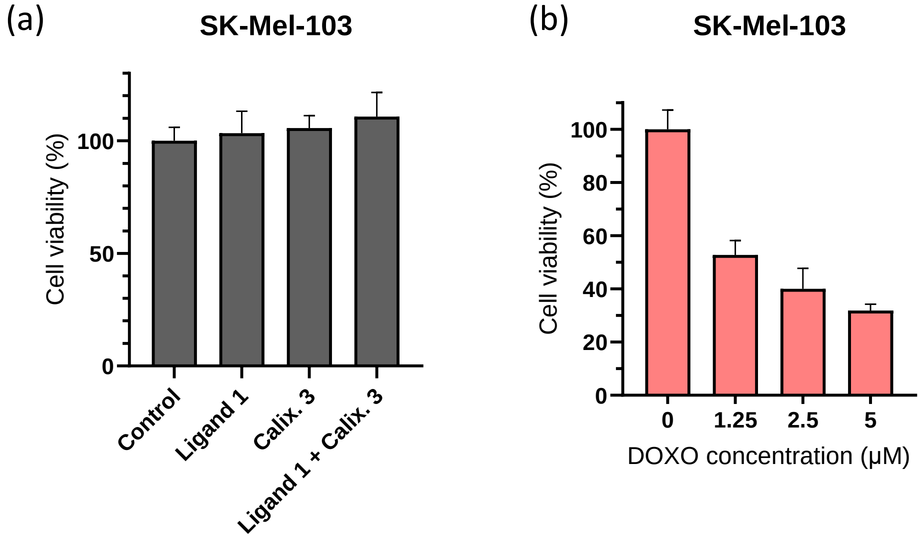

Figure S37. Control cell viability experiments measured by WST-1. Data represented as mean±SEM (n=3). (a) Toxicity assay with cage components: 100 µM Ligand **1**, 50 µM Calixarene **3**, and 100 µM Ligand **1** + 50 µM Calixarene **3**. (b) Treatment with 50 µM Calixarene **3**, 100 µM Ligand **1** and increasing concentrations of DOXO.

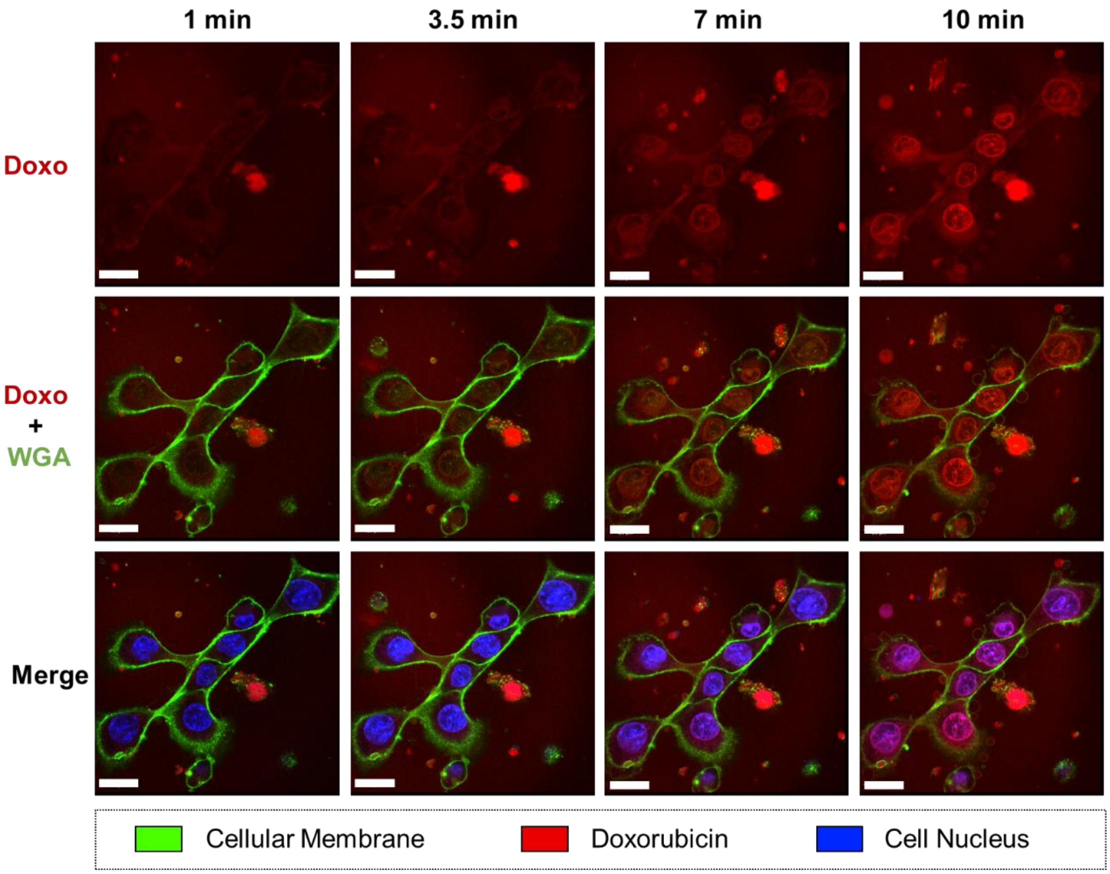

Figure S38. Time-lapse confocal images of SK-Mel-103 cells incubated with Hoechst (blue nuclei marker), WGA (green membrane marker) and treated with free DOXO (fluorescent red) at 5 µM up to 10 min. Scale bar represents 20 µm.

References

S1 L. Zhao, Y. Liu, C. He, J. Wang, C. Duan, *Dalton Trans.* **2014**, *43*, 335−343. (DOI: https://doi.org/10.1039/C3DT51900G)

S2 S. Bandi, D. K. Chand, *Chem. Eur. J.* **2016**, *22*, 10330–10335. (DOI: https://doi.org/10.1002/chem.201602039)

S3 W. D. J. Tremlett, T. Söhnel, J. D. Crowley, L. J. Wright, C. G. Hartinger, *Inorg. Chem.* **2023**, *62(8)*, 3616−3628. (DOI: https://doi.org/10.1021/acs.inorgchem.2c04399)

S4 M. Grajda, M. Wierzbicki, P. Cmoch, A. Szumna, *J. Org. Chem.* **2013**, *78(22)*, 11597–11601. (DOI: https://doi.org/10.1021/jo4019182)

S5 L. Abis, E. Dalcanale, A. Du Vosel, S. Spera, *J. Org. Chem.* **1988**, *53(23)*, 5475–5479. (DOI: https://doi.org/10.1021/jo00258a015)

S6 V. S. Yashchenko, A. A. Pap, G. V. Kalechits, A. V. Makey, V. K. Ol'khovik, *Chem. Heterocycl. Comp*. **2015**, *50*, 1471−1477. (DOI: https://doi.org/10.1007/s10593-014-1612-2)

S7 L. Zhang, H. Yi, J. Wanga, A. Lei, *Green Chem.* **2016**, *18*, 5122−5126. (DOI: https://doi.org/10.1039/C6GC01880G)

S8 N. Tada, Y. Ikebata, T. Nobuta, S. Hirashima, T. Miura, A. Itoh, *Photochem. Photobiol. Sci.* **2012**, *11*, 616−619. (DOI: https://doi.org/10.1039/c2pp05387j)

S9 O. V. Dolomanov, L. J. Bourhis, R. J. Gildea, J. A. K. Howard, H. Puschmann, *J. Appl. Cryst.* **2009**, *42*, 339−341. (DOI: https://doi.org/10.1107/S0021889808042726)

S10 G. M. Sheldrick, *Acta Cryst.* **2008**, *A64*, 112−122. (DOI: https://doi.org/10.1107/S0108767307043930)

S11 G. M. Sheldrick, *Acta Cryst.* **2015**, *C71*, 3−8. (DOI: https://doi.org/10.1107/S2053229614024218)

S12 D. P. August, G. S. Nichol, P. J. Lusby, *Angew. Chem. Int. Ed.* **2016**, *55*, 15022–15026. (DOI: https://doi.org/10.1002/anie.201608229)

S13 J. S. Mugridge, R. G. Bergman, K. N. Raymond, *J. Am. Chem. Soc.* **2011**, *133*, 11205–11212. (DOI: https://doi.org/10.1021/ja202254x)

S14 G. Montà-González, F. Sancenón, R. Martínez-Máñez, V. Martí-Centelles. *Chem. Rev.* **2022**, *122*, 13636–13708. (DOI: https://doi.org/10.1021/acs.chemrev.2c00198)

S15 J. K. Klosterman, Y. Yamauchi, M. Fujita, *Chem. Soc. Rev.* **2009**, 38, 1714–1725. (DOI: https://doi.org/10.1039/B901261N)

S16 N. Kishida, Y. Tanaka, M. Yoshizawa. *Chem. Eur. J.* **2022**, *28* (69), e202202075. (DOI: https://doi.org/10.1002/chem.202202075)

S17 D. Rehm, A. Weller, *Chemie Z. Phys. Chem.* **1970**, *69*, 183-200. (DOI: https://doi.org/10.1524/zpch.1970.69.3_4.183)

S18 G. Greco, L. Ulfo, E. Turrini, A. Marconi, P. E. Costantini, T. D. Marforio, E. J. Mattioli, M. Di Giosia, A. Danielli, C. Fimognari, M. Calvaresi, *Cells*, **2023**, *12*, 392. (DOI: https://doi.org/10.3390/cells12030392)

S19 J. Vacek, L. Havran, M. Fojta. *Collect. Czech. Chem. Commun.* **2009**, *74*, 1727-1738. (DOI: https://doi.org/10.1135/cccc2009512)

S20 T. Mikolajczyk, B. Pierozynski, L. Smoczynski, W. Wiczkowski, *Molecules*, **2018**, *23*, 1293. (DOI: https://doi.org/10.3390/molecules23061293)

S21 S. Chen, K. Li, F. Zhao, L. Zhang, M. Pan, Y.-Z. Fan, J. Guo, J. Shi, C.-Y. Su, *Nat. Commun.* **2016**, *7*, 13169. (DOI: https://doi.org/10.1038/ncomms13169)
S22 M. Ehsani, J. Soleymani, P. Mohammadalizadeh, M. Hasanzadeh, A. Jouyban, M. Khoubnasabjafari, Y. Vaez-Gharamaleki. *Microchem. J.* **2021**, *165*, 106101. (DOI: https://doi.org/10.1016/j.microc.2021.106101)

S23 Deppmeier, B. J.; Driessen, A. J.; Hehre, T. S.; Hehre, W. J.; Johnson, J. A.; Klunzinger, P. E.; Leonard, J. M.; Pham, I. N.; Pietro, W. J.;Jianguo, Y. Spartan '20, version 1.0.0 (Mar 8th 2021),Wavefunction Inc., 2011.

S24 V. Martí-Centelles, T. K. Piskorz, F.Duarte, *ChemRxiv* **2024**. (DOI: https://doi.org/10.26434/chemrxiv-2024-fmlx0)
